# Genome-wide prioritization of disease genes and identification of disease-disease associations from an integrated human functional linkage network

**DOI:** 10.1186/gb-2009-10-9-r91

**Published:** 2009-09-03

**Authors:** Bolan Linghu, Evan S Snitkin, Zhenjun Hu, Yu Xia, Charles DeLisi

**Affiliations:** 1 Bioinformatics Program, Boston University, 24 Cummington Street, Boston, MA 02215, USA; 2 Department of Chemistry, Boston University, 590 Commonwealth Avenue, Boston, MA 02215, USA

## Abstract

We integrate 16 genomic features to construct an evidence-weighted functional-linkage network comprising 21,657 human genes. The functional-linkage network is used to prioritize candidate genes for 110 diseases, and to reliably disclose hidden associations between disease pairs having dissimilar phenotypes, such as hypercholesterolemia and Alzheimer's disease. Many of these disease-disease associations are supported by epidemiology, but with no previous genetic basis. Such associations can drive novel hypotheses on molecular mechanisms of diseases and therapies.

## Abstract 2

An evidence-weighted functional-linkage network of human genes reveals associations among diseases that share no known disease genes and have dissimilar phenotypes

## Background

Recently, a number of computational approaches have been developed to predict or prioritize candidate disease genes [1-34]. Most approaches are based on the idea that genes associated with the same or related disease phenotypes tend to participate in common functional modules (such as protein complexes, metabolic pathways, developmental or organogenesis processes, and so on) [1-16]. This concept is supported by functional analysis of genes associated with diverse diseases [1-4], and by the success of various disease gene prioritization studies based on the concept [5-7,9-17,19,20,23,24,29].

Network-based approaches have also been employed to infer new candidate disease genes based upon network linkages with known disease genes [15,17-23]. These methods typically first construct a gene-gene association network based on one or more types of genomic and proteomic data, and subsequently rank candidate genes based on network proximity to known disease associated genes. Although some of these methods perform well using just one specific type of evidence for functional association, such as protein-protein physical-interaction data or co-expression data, the restriction to only one type of functional association potentially limits their predictive ability [17,20-23]. To address this issue, Franke *et al*. [15] constructed a functional linkage network (FLN) by integrating multiple types of data, and utilized the FLN for disease gene prioritization. However, their results indicate that the performance was highly dependent on Gene Ontology (GO) annotations, in addition to functional associations from curated databases such as the Kyoto Encyclopaedia of Genes and Genomes (KEGG) and Reactome [15,35,36]. As a result, the predictions tend to be biased towards well-characterized genes, and thus limit potential inferences. In a second study, Kohler *et al*. [19] constructed an FLN from heterogeneous data sources, and used a random walk algorithm for disease gene prioritization. However, their network did not incorporate linkage weight to differentiate confidences in functional associations among genes. Therefore, the FLN-based disease gene prioritization still needs to be further explored.

In addition to identifying genes associated with different diseases, other work has explored relationships among human diseases [1-4,37]. Recent studies indicate that human diseases tend to form an interrelated landscape, whereby different diseases are linked together based on perturbing the same biological processes [1-4]. Perhaps unsurprising is the finding that diseases with similar phenotypes tend to be caused by dysfunctions of the same genes [1-4]. Less anticipated was the finding that diseases with dissimilar phenotypes can also be related at the molecular level [1,2]. To study disease-disease relationships, some previous methods used the similarity of phenotype descriptions or examined the hospital diagnosis records to quantify the disease-disease associations [3,37]. However, because these approaches characterize disease-disease associations entirely at the phenotypic level, they have the potential limitation of missing those disease-disease associations that can be easily detected at the molecular level but not at the phenotypic level.

Recently, Goh *et al*. [4] proposed a method to identify disease-disease associations at the molecular level based on shared disease genes, which therefore may capture associations missed by the phenotype-based approaches. However, the breadth of this method is limited by the relative paucity of knowledge of disease causing genes. A potential solution to this problem is the use of functional linkages to identify associations between genes involved in different diseases. This can result in the identification of relationships between diseases that while they may not be associated with the same genes, are associated with functionally related sets of genes.

**Figure 1:**
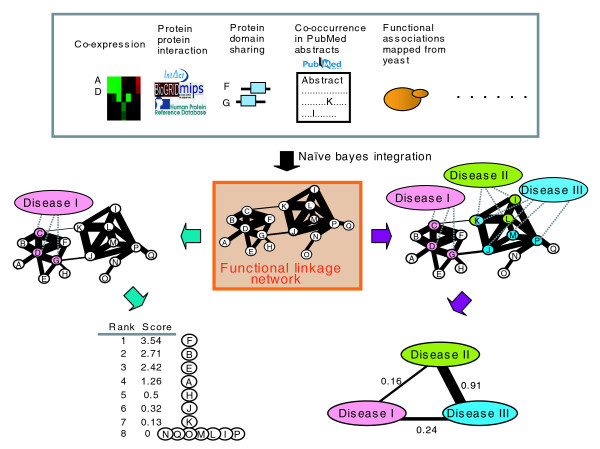
Construction of an integrated functional linkage network (FLN) with applications in prioritizing candidate disease genes and quantifying the disease-disease associations. Functional associations between genes are retrieved from diverse data sources (Table 1). These functional associations are then integrated into one single FLN using a naïve Bayes classifier, in which the nodes represent individual genes and the weighted edges represent the degree of their overall functional association upon combining all contributing data sources. Green arrows represent the two steps of using of the FLN for candidate disease gene prioritization: step 1, given a particular disease (Disease I), label genes known to be associated with this disease as seeds (pink colored nodes); step 2, prioritize all other genes in terms of their association with the disease based on the sum of the weights of their network links to the seed genes. The purple arrows represent the two steps of using the FLN to quantify the disease-disease associations: step 1, label genes known to be associated with different diseases with different colors (gene K is labeled with two colors since it is associated with two diseases); step 2, quantify the associations between any two diseases based on the degree of association between the two corresponding disease gene sets within the FLN.

Here, we construct an integrated FLN in human for two purposes: to prioritize new (not previously recognized) genes that are potentially associated with a given disease; and to explore the inter-relationships between diverse diseases revealed by considering functional associations between genes associated with different diseases (Figure 1). We use a naïve Bayes classifier [38,39] to integrate 16 functional genomics features assembled from 32 sub-features. The result of this integration is a genome-scale FLN (composed of 21,657 genes and 22,388,609 links), in which nodes represent genes, and edge weights the likelihood that the linked nodes participate in a common biological process. Our integrated FLN has a higher coverage and increased accuracy compared to networks based on individual data sources.

Next, we use this FLN to predict new candidate disease genes for 110 diverse diseases from the Online Mendelian Inheritance in Man Database (OMIM) database [40]. For each disease, we quantify the degree of association between each gene and the disease by considering how tightly the candidate gene is connected to known disease genes in the FLN. This then allows us to rank the probabilities of all genes being involved in a particular disease, based upon their degree of functional relatedness to genes known to be associated with a given disease.

Finally, using the FLN, we identify disease-disease associations based on functional correlations between disease-related genes. Specifically, our approach considers not only whether diseases share associated genes, but also whether gene sets from different diseases are tightly linked in the FLN. We show that the FLN can be used to identify associations between phenotypically diverse diseases, and to reveal associations even in the absence of common known disease genes or common pathological symptoms. With knowledge of such disease-disease associations, prior knowledge gained from one disease can shed light on the underlying molecular mechanisms and relevant therapies of related diseases.

## Results

### A genome-scale human functional linkage network built through data integration

Our goals are to exploit the functional coherence of genes involved in a given disease to identify genes that underlie diverse disorders, and to find previously unknown links between phenotypically dissimilar human diseases. We pursue these goals by first integrating genomic features from disparate data sources to establish quantitative functional links among human genes. Since each data source usually characterizes only one type of functional association between genes, and covers a relatively limited set of genes, functional associations from various sources need to be combined to attain maximal coverage and accuracy. We systematically assemble a set of 16 genomic features, which incorporates 32 sub-features. These genomic features include diverse functional genomics data in human, as well as functional associations mapped through orthology from five model organisms (yeast, worm, fly, mouse, and rat; Table 1).

**Table 1:** Data sources for FLN construction

**Data sources**	**Description**	**Number of unique gene pairs**	**Number of unique genes**
Curated PPI	Curated human PPI from HPRD, BIND, BIOGRID, INTACT, MIPS, DIPS, and MINT [45-51]	90,352	10,281
Y2H	PPI from high-throughput yeast two-hybrid experiments [52]	2,611	1,522
Masspec	PPI from large-scale mass spectrometry experiments [53]	2,046	1,159
DDI	Protein pairs containing interacting protein domains [38,39]	6,933,469	13,454
Co-exp	Expression correlation from multiple large-scale expression datasets [56,88-90]	5,110,798	16,287
DS	Proteins pairs sharing same protein domains [91]	2,064,262	17,328
PG	Gene pairs having correlated phylogenetic profiles [56]	18,086	2,607
GN	Gene pairs located close to each other along the chromosome [56]	10,070	1,365
Fusion	Protein pairs fused into one single protein in other species [56]	361	361
Yeast	Functional associations mapped from seven types of functional genomics data in yeast through gene orthology [92]	123,380	3,809
Worm	Functional associations mapped from four types of functional genomics data in worm through gene orthology [41]	96,911	5,737
Fly	Functional associations mapped from three types of functional genomics data in fly through gene orthology [56]	139,984	5,966
Mouse-rat	Functional associations mapped from three types of functional genomics data in mouse and rat through gene orthology [56]	254,477	11,789
TexM	Co-occurrence in PubMed abstracts [56]	518,716	12,286
MF	Gene pairs sharing same molecular function terms in GO [93]	6,937,725	7,863
CC	Gene pairs sharing same cellular component terms in GO [93]	5,591,796	12,503

See Additional data file 5 for detailed descriptions of data sources for FLN construction. CC, cellular component; Co-exp, co-expressed; DDI, domain-domain interaction; DS, protein domain sharing; GN, gene neighbor; HPRD, Human Protein Reference Database; Masspec, mass spectrometry; MF, molecular function; MIPS, Munich Information Center for Protein Sequences; PG, phylogenetic profiles; PPI, protein-protein interaction; TexM, text mining; Y2H, yeast two hybrid experiments.

**Figure 2:**
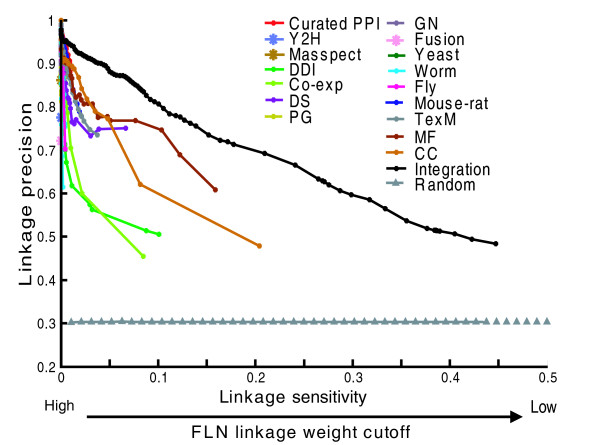
Data integration outperforms individual data sources in terms of quantifying functional links between human genes. The x-axis represents linkage sensitivity, defined as the fraction of the gold standard positive (GSP) gene pairs that are linked at different linkage weight cutoffs (see Materials and methods). The y-axis represents linkage precision, defined as the fraction of the linked gold standard gene pairs that belong to the GSP set (see Materials and methods). GSPs are defined as gene pairs sharing the same biological process term in Gene Ontology (GO). Gold-standard negatives (GSNs) are defined as gene pairs annotated with GO biological process terms that do not share any term. To generate the random control curve, we randomize the class labels in the gold standard datasets and then perform the same evaluation. In Figure S1 of Additional data file 5, we provide the same plot with the x-axis in log scale to show details for individual data sources. CC, cellular component; Co-exp, co-expressed; DDI, domain-domain interaction; DS, protein domain sharing; GN, gene neighbor; Masspect, mass spectrometry; MF, molecular function; PG, phylogenetic profiles; PPI, protein-protein interaction; TexM, text mining; Y2H, yeast two hybrid experiments. The descriptions of the 16 individual data sources are listed in Table 1.

We then use a naïve Bayes classifier to compute functional links between human genes by integrating these genomic features. Each functional link is weighted by a log likelihood ratio (LR) score, which reflects the probability of the linked gene pair sharing the same biological process after summing over evidence from all available data sources (see Materials and methods). Such extensive data integration outperforms individual data sources in terms of inferring functional linkages (Figure 2), demonstrating the importance of data integration.

After data integration, we choose a permissive linkage weight cutoff (LR score is higher than 1; Equation 1 in the Materials and methods) such that two genes are linked if the overall evidence supports the functional linkage. This threshold is very intuitive, as it retains edges with more evidence for functional association than against it, and removes edges with more evidence against functional association than for it. In addition, the same cutoff has also been successfully used by Lee *et al*. [41] to predict perturbation phenotypes of genes based on an integrated FLN in worm. The resulting genome-scale FLN network consists of 21,657 genes (covering approximately 85% of RefSeq annotated human genes [42]) and 22,388,609 weighted links (Additional data file 1). Despite its high coverage, our network retains its accuracy because each link is weighted and the linkage weight is proportional to the linkage precision (Figure 2). The average number of linked neighbours per gene is around 2,000. Such high linkage density together with linkage weighting allows a quantification of functional associations between thousands of genes. Such high coverage is critical to the successful utilization of the FLN for both disease gene prioritization and mapping the disease-disease associations at the molecular level.

### Identifying candidate disease-associated genes

Given a network of functionally linked genes, our first goal is to use the information in this network to identify genes most likely to be associated with a particular disease. The motivation for using the FLN to identify potentially disease-related-genes is the hypothesis that genes whose dysfunction contributes to a disease phenotype tend to be functionally related [1-16]. Our approach exploits this concept by using genes known to be associated with a particular disease as network 'seeds', and identifying those genes whose connectivity with the seeds indicates a strong functional relation. In particular, for a given disease, each gene in the network is prioritized according to the sum of the weights of its network links to the known disease (seed) genes (Equation 4; see Materials and methods) [41,43]. This prioritization rule is referred to as neighbourhood weighting.

### Validation of identified candidate disease-associated genes

To test this approach, we first extract from the OMIM database [40] 1,025 known disease genes, and assemble them into 110 seed gene sets, representing 110 disorders covering a wide spectrum of human disease phenotypes (Additional data file 2). Each seed set contains at least 5 genes, and the average seed count is 11 (with some seed genes associated with more than one disease). Next we use the FLN to identify new candidate disease genes for each of the 110 diseases, based on the neighbourhood weighting rule [41,43,44]. As a result, on average, nearly half of the genome is prioritized for each disease. In Additional data file 3, we list the top 100 ranked new candidate disease genes for each disease. To help investigators estimate the prediction precision at a particular rank cutoff, for each disease we provide a plot of precision estimate versus different rank cutoffs (see Materials and methods; Additional data file 4). We assess the performance of our FLN-based disease gene prediction method using leave-one-out cross validation, with so-called disease-centric [41,43] and gene-centric approaches [19,23] (see Materials and methods).

#### Disease centric assessment

**Figure 3:**
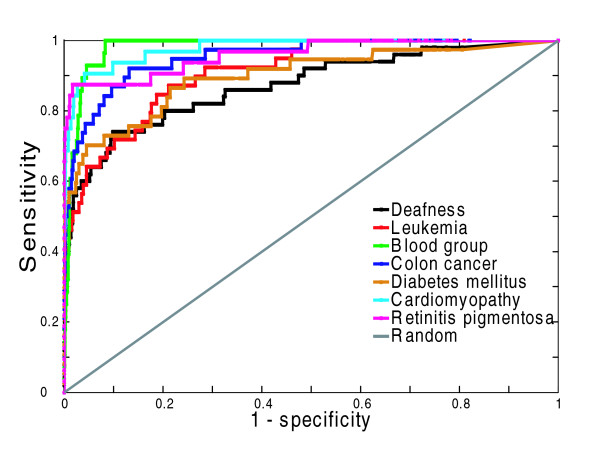
Predictability of seven example diseases evaluated by ROC curves in disease-centric assessment. Prediction performance for individual diseases is measured by the true positive rate (sensitivity) versus false positive rate (1 - specificity). In particular, for each given disease, each gene in the network is ranked based on the disease association score (*S*_*i*_; Equation 4). The *S*_*i *_for each known disease (seed) gene is computed using leave-one-out cross-validation, based on its connectivity to other seeds. Next the performance for each disease is assessed by calculating the sensitivity (True positives/(True positives + False negatives)) and 1 - specificity (False positives/(True negatives + False positives)) at different *S*_*i *_cutoffs. Here True positives is the number of seed genes above the *S*_*i *_cutoff, False positives is the number of non-seed genes above the cutoff, True negatives is the number of non-seed genes below the cutoff, and False negatives is the number of seed genes below the cutoff. Random prediction performance is indicated by the diagonal.

The disease-centric evaluation approach first ranks each gene based on the neighborhood weighting rule for a particular disease, and then for each disease computes the area under the receiver operating characteristic (ROC) curve (AUC), which is obtained by varying the rank cutoff (see Materials and methods) [43]. The AUC is an indication of how highly in the ranked list the known disease genes are, where the AUC will be 1 if all disease genes are at the top of the list and 0.5 if the disease genes are randomly distributed in the list. Examples of ROC curves for seven diseases are provided in Figure 3. Additionally, we also provide the same plot using just the extreme left side of the ROC curve, which represents the top ranking predictions (Figure S2 of Additional data file 5).

**Figure 4:**
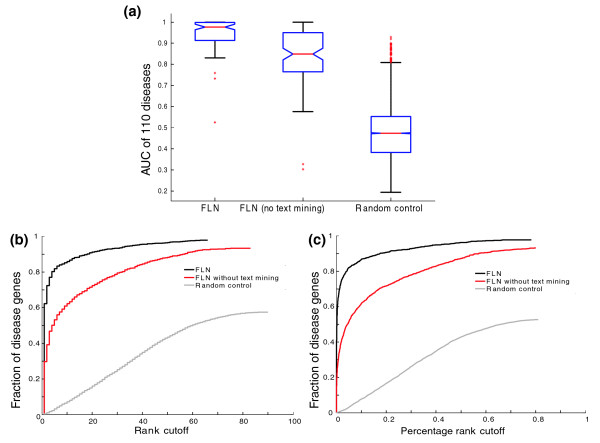
FLN-based disease gene prioritization significantly outperforms random control. Performances are compared between FLN (inclusion or exclusion of text mining data) based disease-gene prioritization and the random control. The random control is generated using the FLN to prioritize randomly assembled disease gene sets (see Materials and methods). **(a) **Box plots of AUCs of disease gene prioritization performances for 110 diseases, based on disease-centric assessment (see Materials and methods). For each box plot, the bottom, middle, and top lines of the box represent the first quartile, the median, and the third quartile, respectively; whiskers represent 1.5 times the inter-quartile range; red plus signs represent outliers. **(b) **Disease gene prioritization performance based on gene-centric assessment using the artificial chromosome region background (see Materials and methods). Gene-centric assessment treats each known gene-disease association as a test case. For each test case, the task is to assess how well the known disease (seed) gene ranks relative to a background gene set according to the disease-association score (*S*_*i*_; Equation 4). The *S*_*i *_for each gene in each test case is calculated in leave-one-out setting based on the connectivity to the remaining seed genes. The background gene set used is referred to as the artificial chromosomal region, which is composed of a collection of 100 nearest genes flanking the tested disease gene physically on the chromosome. Finally, after the rank of each tested disease gene for each test case is determined, all the test cases are pooled together and the overall performance is assessed by evaluating the fraction of the tested disease genes ranked above various rank cutoffs. **(c) **Same evaluation as (b) using the background gene set composed of all the genes represented in the FLN, as opposed to just those proximate on the chromosome.

Disease-centric evaluation shows that FLN-based disease gene prioritization has an extremely high median AUC of 0.98 for the 110 diseases tested, indicating a high predictive capacity across a large number of diseases (Figure 4a). In light of this very high performance, we next consider an issue that may potentially inflate our performance. Specifically, one of the data sources used to construct our FLN is text mining of PubMed abstracts. The potential issue with this text mining feature is the possibility that some of the gene-disease associations in the OMIM database, which we use to evaluate our method, could be originally derived from the same literature references that text mining is based on [19,24]. To assess the impact of this potential bias, we create a FLN excluding text mining, and find that the resulting AUCs have a median value of 0.85, lower than full FLN, but still far superior to the random expectation of 0.5. In particular, when we exclude text mining data from the FLN, 80%, 65%, and 39% of the diseases still have an AUC of over 0.75, 0.8, and 0.9, respectively. Additionally, we have also performed the disease centric analysis using only the area of the extreme left side of the ROC curve, which represents the top ranking predictions (see Additional data file 5 for the ROC-50 analysis). The results are consistent with those using the whole ROC curve (Figure S3 of Additional data file 5). Therefore, our FLN is capable of predicting candidate genes for diverse diseases, even in the absence of text mining data.

#### Gene-centric assessment

This evaluation treats each known gene-disease association as a test case, and assesses how well each known disease gene ranks relative to a background set of genes not known to be associated with the particular disease (see Materials and methods). Then, all test cases are pooled together, and the overall performance is evaluated by calculating the fraction of tested disease genes that are ranked above various rank cutoffs. We use two background sets to define the background pool of candidate genes from which we pick out disease-associated genes. One set is a collection of 100 nearest genes flanking the test disease gene physically on the chromosome. This background is referred to as the artificial chromosome region background, and is intended to mimic the common scenario in which a chromosomal region is known to be associated with a disease through genetic association studies, but the specific disease-causing genes are unknown. The other background set contains all genes in the network and is intended to mimic the common scenario where the set of potential candidate genes cannot be narrowed down. This set is referred to as the genome background.

With the artificial chromosome region background, 85% (with text mining) or 62% (without text mining) of disease genes are ranked in the top 10 out of 100 (Figure 4b). Moreover, we calculate the fold enrichment score, defined as the average rank of a gene before prioritization divided by the rank after prioritization (see Materials and methods). The average fold enrichments are 35.5 (with text mining) or 20.5 (without text mining). Finally, we also carry out gene-centric evaluation using the genome background, and the results are similar (Figure 4c).

### Monogeneic versus polygeneic disorders

Next, we investigate the difference in prioritization performance between monogenic diseases and complex diseases. It is potentially important to distinguish these two classes of diseases, as complex diseases tend to be caused by dysfunctions in multiple biological processes, and this lack of functional coherence may reduce the utility of the FLN in predicting novel disease genes. Kohler *et al*. [19] published a gene-disease association benchmark dataset that explicitly separates monogenic diseases (83 diseases), polygenic diseases (12 diseases) and cancers (12 diseases). We adopt their categorization so that we can evaluate the three disease groups separately. In particular, using the gene-centric evaluation (see Materials and methods), we evaluate how well each known disease gene is ranked relative to a background gene set in a leave-one-out cross-validation (see Materials and methods). The background gene set is composed of the 100 nearest genes flanking the test disease gene physically on the chromosome. As expected, the results are best for monogeneic diseases (Figure S4 in Additional data file 5). The lower performance for complex diseases and cancers is not surprising, since disease gene prediction methods are based on the assumption of functional coherence among genes contributing to the same disease, while as mentioned above, complex diseases tend to perturb multiple biological processes, making the contributing genes less functionally coherent. Despite the lower performance for complex disorders, the results are still far better than random control; for example, 65% of tested disease genes ranked in the top 20 among the background gene set composed of 100 genes.

### The importance of data integration for gene prioritization

Disease genes have previously been prioritized using network-based strategies that used only protein-protein interactions (PPI) [20,21,23]. Here we have integrated multiple data sources with the expectation that such integration will improve performance. To assess whether this is in fact the case, we compare disease-prioritization performances between our FLN and a PPI network that combines human PPI links from seven major curated PPI databases [45-51], along with high-throughput PPI data from yeast two-hybrid and mass spectrometry [52-54], and interactions mapped from PPI of other model organisms [55]. To avoid bias, interactions from different sources in the PPI network are weighted using the same procedure as FLN construction (Equation S1 in Additional data file 5). In the end, a total of 105,361 interactions among 11,886 genes are included in the PPI network.

**Figure 5:**
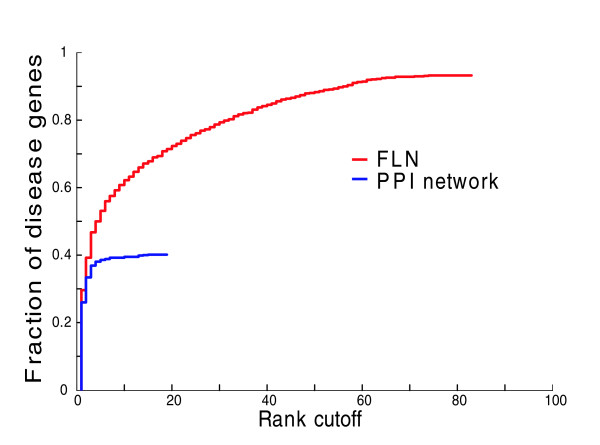
FLN-based disease gene prioritization shows improvement over PPI network. Gene-centric assessment (as described in the legend of Figure 4b) is used to compare the disease-gene prioritization performances between the integrated FLN and a representative PPI network. The PPI network is composed of curated PPI databases [45-51], along with high-throughput PPI data from yeast two-hybrid and mass spectrometry [52-54], and interactions mapped from PPI of other model organisms. The artificial chromosomal region composed of a collection of 100 nearest genes flanking each tested disease gene physically on the chromosome is used as the background gene set. Here, we exclude text mining data from the FLN.

As expected, data integration does improve performance (Figure 5). Using the gene-centric evaluation with the artificial chromosome region background, 62% of disease genes rank in the top 10 among 100 using the integrated FLN (excluding text miming), in contrast to 40% in the PPI network. Similar results were also found using the disease-centric assessment (Figure S5a in Additional data file 5; see Materials and methods for the description of disease-centric assessment). Further support for using an FLN-based approach is the increased gene coverage. In the PPI network only 40% of disease genes are connected to seed genes, and thus only 40% can be prioritized. In contrast, in the integrated network more than 92% of disease genes are linked to seeds and can, therefore, be prioritized. Finally, the benefit of data integration is also evident when we evaluate the prioritization performance of the FLN at different linkage weight cutoffs. After the application of the permissive linkage weight cutoff (LR > 1; Equation 1), we explore other higher cutoffs but find no improvement in the prioritization performance (Figure S5b, c in Additional data file 5). This further demonstrates that functional links are assigned proper weights after data integration, and that the neighbourhood weighting decision rule (Equation 4) allows links with lower weights to contribute to performance.

### Evaluation of new predictions using recently identified disease genes

The performance evaluations described above are based on leave-one-out cross-validation. Here we evaluate the predictive performance for unknown disease genes by simulating the search for new disease genes. We first manually check the date of the landmark reference for each gene-disease association recorded in the OMIM database. Next, disease genes with references published after January 2007 are set aside for testing, while all other disease genes with reference dates before 2007 are used for seed genes. For the purpose of this evaluation we included text mining from the STRING database, which was curated before January 2007 [56].

Within the FLN, there are a total of 61 disease genes associated with 31 diseases that are published after January 2007. These are used for evaluating the FLN-based predictions. Among them, 45 disease genes associated with 24 diseases are also present in the representative PPI network. These genes are used for evaluating PPI-based predictions. These recently identified disease genes and their landmark references are listed in Additional data file 6.

**Figure 6:**
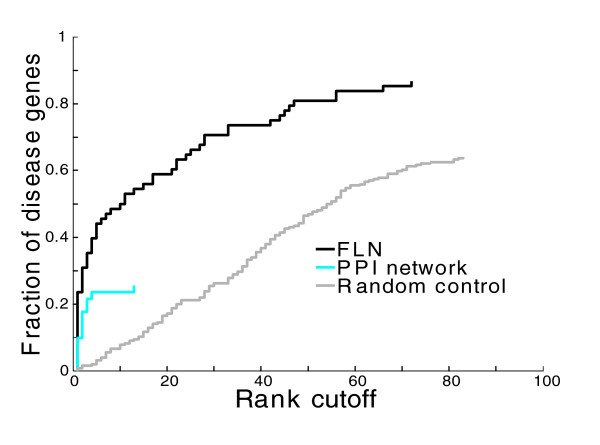
FLN shows improvement over PPI network for predicting 'new' disease genes. Disease genes whose disease-association landmark references were published before January 2007 are considered 'known' and are used as seed genes, and disease genes that were published after January 2007 are considered 'new' and are used as test genes. Performances are compared among FLN, PPI network, and the random control of the FLN. Gene-centric assessment is used to evaluate the performance using the artificial chromosome region background composed of 100 genes, as described in the legend of Figure 4b.

Again, the FLN shows improvement over the PPI network (Figure 6). For instance, using the gene-centric evaluation with the artificial chromosome region background, 45% of disease genes are ranked in the top 5 in the FLN, in contrast to fewer than 25% in the PPI network. It is noteworthy that there is a drop in performance for both PPI and FLN relative to the cross-validation analysis presented above. In particular, the fold enrichment drops from 16 to 8.2 for PPI and from 35.5 to 16.5 for FLN. This indicates that cross-validation tends to overestimate performance, and it is important to consider this when interpreting cross-validation results.

### Obesity: a case study

Obesity is a polygenic disorder involving genes from various processes, such as nutrient catabolism and appetite control [57]. Our FLN includes 24 obesity-associated genes in the OMIM database, and 334 additional obesity-associated genes collected from the literature by Hancock *et al*. [58]. We will subsequently refer to this set of 334 genes as 'ObesHancock' genes. There is no overlap between the 24 OMIM obesity genes and the 334 ObesHancock genes. Here, we rank obesity-related genes using the 24 OMIM genes as seeds, then evaluate the utility of our ranking using the non-overlapping set of ObesHancock genes. Since ObesHancock genes are collected from the literature, we exclude the text mining data source from FLN construction.

We find that the ObesHanecok set is overrepresented in the top scoring FLN genes, with 22 of them occurring in the top 100 (*P *< 1.0 × 10^-13^; see Additional data file 5 for *P*-value calculation). The list of the 22 ObesHancock genes and their supporting evidence are provided in Additional data file 7. Detailed analysis of the subset of the top 100 ranked obesity genes that does not overlap with the ObesHancock set reveals additional genes with potential roles in obesity. For instance, *NR1H3*, ranked 24th, is predominantly expressed in adipose tissue and plays an important role in cholesterol, lipid, and carbohydrate metabolism [59-63]. Recently, Dahlman *et al*. [64] also found that one *NR1H3 *single nucleotide polymorphism (SNP), rs2279238, is associated with the obesity phenotype. Similarly, *NMUR2*, ranked 35th, is exclusively expressed in the central nervous system as a receptor for neuromedin U, a neuropeptide regulating feeding behavior and body weight [65]. Additionally, Schmolz *et al*. [66] found that a *NMUR2 *variant potentially related to obesity in a mouse model.

### FLN-based identification of disease-disease associations at the molecular level

As described in the Introduction, human diseases tend to form an interrelated landscape. We hypothesize that the basis for these relationships stems from multiple diseases resulting from dysfunctions in the same genes, and more broadly, multiple diseases resulting from dysfunction of the same or related biological processes [1-4]. Associations between diseases potentially stemming from common causal genes were previously reported by Goh *et al*. [4]. Here we focus on quantifying associations between diseases based on perturbation in common biological processes by developing the concept of 'mutual predictability' (see Materials and methods). The mutual predictability between two diseases measures the extent to which genes known to be associated with either member of a disease pair can be used to identify genes known to be associated with the other member (see Materials and methods). We hypothesize that disease pairs with high mutual predictability will be closely related to each other, as a high mutual predictability should be indicative of high connectivity in the FLN between the two gene sets associated with two diseases, and hence should quantify the functional relatedness between diseases.

**Figure 7:**
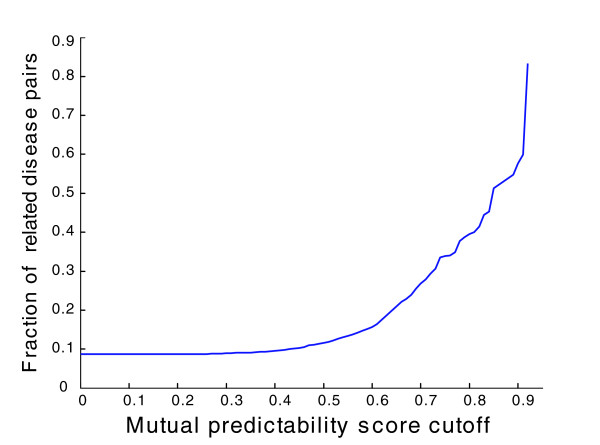
Fraction of related disease pairs increases as mutual predictability cutoffs increase. Disease pairs are considered to be related if they belong to the same disease class based on Goh *et al*.'s manual classification [4].

We validate our mutual-predictability-based disease-disease associations at the molecular and gene network level, using disease-disease associations based on the classification in Goh *et al*. [4], where the diseases in OMIM were manually partitioned into 22 classes based on physiological system-level phenotypic observations. After calculating the mutual predictability (Equation 7) between every possible disease pair (all mutual predictability scores are provided in Additional data file 8), we threshold pair selection with increasing score cutoffs. At each cutoff, we examine the fraction of disease pairs belonging to the same disease class (excluding 'unclassified class' and 'multiple class'; the former has insufficient information for disease class assignment, and the latter lacks physiological system specificity). As seen in Figure 7, the fraction of pairs placed in the categories defined by Goh *et al*. increases rapidly with increasing score cutoff. This demonstrates that FLN-based mutual predictability can capture disease-disease association in a quantitative way.

### The FLN discloses hidden associations between diseases sharing no known disease genes and having dissimilar phenotypes

**Figure 8:**
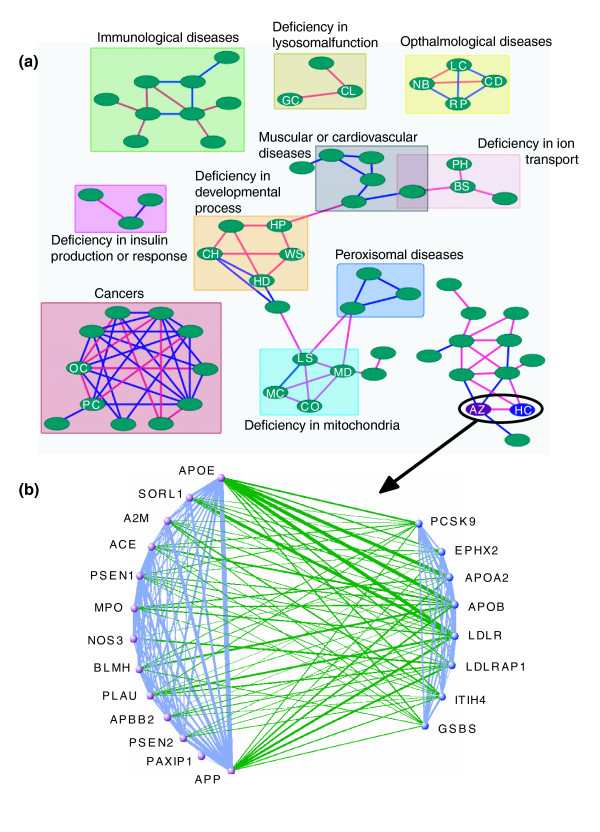
FLN-based disease-disease association network. **(a) **An example network of 66 diseases (nodes) linked by the top 100 edges with the highest mutual predictability scores. Many of these edges cannot be identified using the simple disease-gene sharing method, and these are colored red. The rest of the edges are colored blue, which link disease pairs with one or more overlapping disease genes. This disease-disease association network has a modular topology, and diseases tend to form clusters (rectangles). The disease clusters are labeled based on enriched disease class or underlying molecular mechanisms. The following disease nodes mentioned in the text are labeled: ovarian cancer (OC), prostate cancer (PC), Leber congenital amaurosis (LC), cone or cone-rod dystrophy (CD), retinitis pigmentosa (RP), night blindness (NB), Leigh syndrome (LS), mitochondrial DNA depletion syndrome (MD), combined oxidative phosphorylation deficiency (CO), mitochondrial complex I/II/III deficiency (MC), Alzheimer's disease (AZ), hypercholesterolemia (HC), pseudohypoaldosteronism (PH), Bartter syndrome (BS), holoprosencephaly (HP), Waardenburg syndrome (WS), central hypoventilation syndrome (CH), Hirschsprung disease (HD), ceroid-lipofuscinosis (CL), and glaucoma (GC). **(b) **Functional links among Alzheimer's disease genes (purple nodes) and hypercholesterolemia disease genes (blue nodes) in the FLN. Green edges represent functional links between Alzheimer's disease genes and hypercholesterolemia genes. Light blue edges represent functional links connecting genes associated with the same disease. Edge thickness is proportional to linkage weight. Both networks are generated by Visant [76].

To visualize disease-disease association estimated by mutual predictability, we create a network of disease associations, in which the nodes represent individual diseases and the weighted edges represent mutual predictability. Figure 8a shows a high confidence subset of the disease network obtained by selecting the top 100 pairs (out of 5,995, that is, the top 1.7%; Figure S9 of Additional data file 5), corresponding to a mutual predictability cutoff of approximately 0.85. These 100 pairs cover a total of 66 diseases. At this cutoff, the disease pairs are four times more likely to share the same disease class than expected at random (Figure 7). Moreover, 97 of the 100 disease pairs are supported by various types of evidence, such as the classification scheme of Goh *et al*. (within the same disease class) or other literature evidence (Additional data file 9). These results suggest that disease pairs with high mutual predictability tend to be related.

We compare our method with another available disease-disease association identification method proposed by Goh *et al*., which identifies the associations between two diseases by counting their overlapping disease genes [4]. However, Goh *et al*.'s method could only identify the associations between diseases with known overlapping disease genes. In contrast, our method is able to identify additional associations for those diseases sharing no known disease genes but having dense functional links between their corresponding disease gene sets by taking advantage of the FLN.

Among the 97 potentially related disease pairs with literature support, 48 pairs share known disease genes and are identified by both methods (pairs connected by blue links in Figure 8a). However, the remaining 49 pairs share no known disease genes, and their associations are identified solely based upon functional links among associated genes (pairs connected by red links in Figure 8a). An example of a non-trivial disease-disease linkage in the latter group is the association between Alzheimer's disease, a neurological disorder, and hypercholesterolemia, a metabolic disorder. Since the two diseases share no disease genes in OMIM, their predicted association is based entirely on the strong and dense functional links between the corresponding disease gene sets (Figure 8b). Importantly, associations between diseases identified using the FLN provide immediate insight into the molecular mechanisms underlying different diseases, and thus generate novel hypotheses for therapeutic strategies. For instance, based on the association of hypercholesterolemia and Alzheimer's diseases, we propose that high cholesterol may play an important role in the development of Alzheimer's disease and that modulation of cholesterol levels might help to reduce or delay the risk of Alzheimer's disease, which is indeed supported by recent literature [67-69]. Besides Alzheimer's disease and hypercholesterolemia, there are diverse disease-disease associations that are identified only by the FLN but not by the disease gene sharing method. These include night blindness/Leber's congenital amaurosis, which are both ophthalmological; pseudohypoaldosteronism/Bartter syndrome - both involved in ion transport deficiency); and holoprosencephaly/Waardenburg syndrome - both involved in developmental deficiencies (Additional data file 9). We provide a more quantitative comparison between our mutual predictability method and disease gene sharing method in Additional data file 5.

Since our disease-disease association identified at the molecular level correlates with disease-disease associations based on phenotypic level classification (Figure 7), it is not surprising that some diseases in the same disease class are found to be connected in our disease network. For example, prostate cancer and ovarian cancer both belong to the cancer class, and are connected in our network. Potentially of more interest is the observation that among the 97 potentially associated disease pairs identified by high mutual predictability and supported by the literature, 54 disease pairs belong to different disease classes, as defined by Goh *et al*. [4]. This finding suggests that diseases with different phenotypes can share common etiology at the molecular level [1,2]. Examples of such disease pairs include Alzheimer's disease (neurological class) and hypercholesterolemia (metabolic class) described above, central hypoventilation syndrome (respiratory class) and Hirschsprung disease (gastrointestinal class), both of which are potentially caused by a defective developmental process [2], and ceroid-lipofuscinosis (neurological class) and glaucoma (ophthamological class), both of which are involved in abnormalities in lysosomal function [70,71] (Additional data file 9). These results suggest that standard disease classifications may be much less informative than currently thought.

In addition to validating individual links, we visualize the topology of our disease-disease association network based on the network connectivity. We find that some network modules (such as those defined by rectangles in Figure 8a) can be readily identified through visual inspection. Specifically, diseases within a module tend to be more connected than diseases between different network modules. These disease modules tend to be enriched with homogeneous diseases belonging to the same or related classes, or sharing similar molecular mechanisms (Figure 8a, homogeneous modules are colored correspondingly; cluster details are listed in Additional data file 10 and Figure S9 in Additional data file 5). Examples are the ophthalmological disease clique of night blindness/Leber's congenital amaurosis/retinitis pigmentosa/cone or cone-rod dystrophy, and the mitochondrion deficiency clique of mitochondrial complex I/II/III deficiency/combined oxidative phosphorylation deficiency/Leigh syndrome/mitochondrial DNA depletion syndrome. These results confirm that the FLN-based mutual predictability measure can be used to identify biologically meaningful disease-disease associations.

## Discussion

### Comparison with other disease gene prioritization methods

We have demonstrated that our FLN-based disease gene prioritization approach successfully predicts new candidate disease genes for diverse diseases. The method compares favorably to previous methods, providing 50% more coverage than [19] with accuracy comparable to [19,24,72]. (Detailed descriptions of the performance comparisons are provided in Additional data file 5.)

A potential strategy for further improving on our predictive performance is to consider not only functional linkages between neighbors as was done here, but to also incorporate global network topology into disease gene identification [19]. It should be noted, however, that the use of a global propagation scheme should be done with extra care. A global propagation scheme may more comprehensively exploit the information contained in the FLN by considering non-adjacent nodes, but at the same time it can be more susceptible to noise. Such issues are exemplified in recent work by Wu *et al*. [23], where they compared a direct interaction based local rule and a shortest path rule, based on their abilities to prioritize disease genes. Surprisingly, the shortest path rule, which uses global network topological information, performs worse than the direct interaction rule [23]. A likely reason is that although, in principle, rules utilizing global network topological information are preferred, they are more sensitive to the false positive and false negative links in the network. In practice, however, a properly implemented local rule can perform well, when combined with a comprehensive and weighted FLN.

### Further considerations for disease gene prioritization

Among the data sources used for constructing the integrated FLN, literature-based text mining, GO annotation (GO annotations in the molecular function and cellular component categories are included in the integration), and curated PPI might be biased towards well characterized genes (Table 1). Similarly, text mining and curated PPI data mapped from mouse and rat (organisms close to human in the evolutionary tree) via orthology may also have the same bias. Consequently, predictions based on these sources tend to be biased towards well characterized genes. On the other hand, the remaining data sources are relatively unbiased, and include sequence data, high-throughput experiments, and functional associations mapped from yeast, worm, and fly (organisms distant to human in the evolutionary tree). Predictions from these latter sources can reveal new disease genes that are not well characterized. To test the extent to which our predictions depend on the relatively biased data sources, we remove them and use the remaining unbiased sources for FLN construction and disease gene prioritization. The prediction performance remains far better than random control (Figure S6 in Additional data file 5). Using the gene-centric assessment, over 40% of disease genes are still ranked within the top 10 positions in the artificial chromosome region background composed of 100 genes. The disease-centric assessment also shows similar results. Therefore, our predictions do not solely rely on the potentially biased text mining, molecular function and cellular component annotations of GO, and curated PPI data sources; other relatively unbiased sources also make significant contributions to the disease gene prediction and biological insights that go beyond well characterized genes.

Our current framework assumes that known disease genes ('seed genes') contribute equally to the disease, but in reality some seed genes may contribute more than others, especially for complex diseases. An example is homozygosity in the *APOE4 *allele, which confers a 40% chance of late-onset Alzheimer's disease [73]. Incorporation of this notion of 'seed strength' into our framework would be desirable. In addition, our framework requires the knowledge of a number of known disease genes as seed genes in order to predict new candidate genes. A further improvement might be to borrow seed genes from closely related diseases when the disease under study does not have enough seeds, adjusting the strength in accordance with how closely related the two diseases are.

Additionally, FLN has been made available in VisANT [74], a web-based platform for the integrative visualization and analysis of biological networks and pathways [75-80]. Users can interactively query the FLN for genes of interest by choosing FLN as the current database in the VisANT toolbar; compare the FLN with other interaction data by updating gene interactions stored in the Predictome database; and filter the FLN with preferred weights and visualize the weight using edge color, edge thickness or both. In addition, we are developing a series of functions to integrate the methods of FLN-based prediction of new disease genes and disease-disease associations into VisANT, including making predictions for the new seed sets provided by users. Each disease will be represented as a metanode [78] - a special type of node that contains associated sub-nodes (disease genes), which allows direct integration of disease information and the FLN. These new functions will extend the applications developed here to other areas, such as the prediction of new proteins for protein complexes, cross-talk between pathways and so on.

### Further considerations for FLN-based identification of disease-disease associations

Here, we carry out a proof-of-principle study with our mutual predictability measure to demonstrate that the functional links provided by the FLN can be used to quantify disease-disease associations at the molecular level as illustrated in the Results section. Further studies are needed to improve our measures as well as to explore related measures of disease-disease associations based on the FLN. Our approach for assessing disease-disease associations requires the knowledge of a few known disease genes for each disease, and cannot be applied to diseases with no known disease genes. In these cases, phenotype-based methods such as [3] are good alternatives if there is enough disease phenotype information available, bearing in mind that it is possible for two diseases to share related molecular pathology mechanisms but be less similar at the phenotypic level [1,2]. In general, it is likely that the identification of disease-disease associations can be further improved by combing our FLN-based approach with phenotype-based approaches.

## Conclusions

In this work, we build a comprehensive and high quality weighted FLN by integrating 16 functional genomics features assembled from 32 sub-features from 6 model organisms. To our knowledge, our integrated human FLN is the most comprehensive to date.

We show that genome-wide disease gene prioritization for diverse diseases can be obtained using our integrated FLN. Top ranking candidate disease genes can be used to guide the design of new genetic association studies. Conversely, in the future, results from genetic association studies can be further integrated into our framework for better disease gene predictions.

We also show that the integrated FLN can be used to identify disease-disease associations, even among diseases that share no known disease genes or have dissimilar phenotypes. Thus, we show that by considering functional linkages between genes involved in different diseases, relationships between diseases that are based on underlying molecular mechanisms can be revealed. Such associations can potentially be used to generate novel hypotheses on the molecular mechanisms of human diseases, and can in turn guide the development of relevant therapy as illustrated in the case of Alzheimer's disease and hypercholesterolemia.

## Materials and methods

### Human dataset

All methods are applied to a common set of 25,564 protein encoding genes (downloaded from RefSeq in November 2007 [42]) with the Entrez gene ID as the unique identifier [81]. For data sources using different gene or protein identifiers, the original identifiers are all mapped to Entrez gene IDs using the cross-reference files from Entrez Gene [81], the International Protein Index (IPI) database [82], or Biomart [83].

### FLN construction

#### Gold standard

We construct gold standard datasets for gene-gene functional association in the following way. Gold-standard positives (GSPs) are defined as gene pairs sharing the same biological process term in GO. Gold-standard negatives (GSNs) are defined as gene pairs annotated by GO biological process terms but that do not share any term. As a result, 1,456,585 pairs are included in GSP, and 3,345,661 pairs are included in GSN. These gene pairs are composed of 5,955 human genes. Details are provided in Additional data files 5 and 11.

#### Naïve Bayes classifier for FLN construction

We use a naïve Bayes classifier [84] to predict gene pairs that participate in the same GO biological process. The inputs to the classifier include diverse genomic features assembled from various data sources (Table 1). Similar to other studies [29,85], we use a log likelihood ratio (LR) score to quantify the degree of functional association between two genes. The associated gene pairs are then assembled into a functional linkage network with individual genes as nodes and the LR score as the linkage weight.

There are two primary reasons that we choose naïve Bayes as the integration approach. First, it allows for the direct integration of heterogeneous data sources in an easily interpretable model. Second, it calculates the probability that two genes participate in the same biological process given the input features. Naïve Bayes classifiers have been used to successfully combine heterogeneous data in human [29,38,39]. It is possible, and perhaps likely, that other multivariate methods would perform equally as well [86].

In the Bayesian framework, we use the LR score to quantify the degree of functional association between a gene pair:

(1)

where *I *is a binary variable representing the existence of functional association (GSP pairs), ~*I *represents the absence of functional association (GSN pairs), *f*_1 _through *f*_*n *_are the genomic features, and LR is the likelihood ratio.

In naïve Bayes, we assume that features are conditionally independent. As a result, *LR*(*f*_1_,..., *f*_*n*_) can be further written as:

(2)

Equation 2 is equivalent to the following, where LLR represents log likelihood ratios:

(3)

Naïve Bayes assumes conditional independence among features, but the naïve Bayes classifier can still be applied even when this assumption is not strictly satisfied [29,85,87]. In our work, we minimize conditional dependence among features by combining related datasets or features into single features before final integration (Additional data file 5). Indeed, no strong correlations exist among our final features after this procedure (Additional data file 12).

As listed in Table 1, a total of 16 features assembled from 32 sub-features are utilized for the final integration (see Additional data file 5 for details).

#### FLN quality assessment

After the naïve Bayes integration, each gene pair is assigned a probability for sharing a GO biological process. The quality of this FLN is evaluated by plotting linkage precision versus linkage sensitivity at various linkage weight cutoffs. Given a pre-specified linkage weight cutoff, 'linkage precision' is defined as the fraction of the linked gold-standard gene pairs that belong to the GSP set, and 'linkage sensitivity' is defined as the fraction of the GSP pairs that are linked. We perform threefold cross-validation by randomly partitioning the gold standard datasets into three equal segments, two serving as training sets and the third as the test set. This procedure is repeated three times such that each segment serves once as the test set. Next, the three test sets are combined and the performance is assessed by plotting linkage precision versus linkage sensitivity. Finally the whole gold standard dataset is used for training to generate one final FLN, which is then used for disease gene prioritization and prediction of disease-disease associations.

### FLN-based disease gene prioritization

For a given disease, the known disease associated genes are used as seeds. New candidate genes can then be ranked based on their association with these seed genes in the FLN.

#### Seed genes

The seed disease genes are obtained from the OMIM database, a compendium of human disease genes and phenotypes [40]. The disease-gene associations are downloaded from the Morbid Map (April 2008) [40]. To obtain reliable test statistics, we include in the analysis only diseases with at least five seed genes in the FLN. This results in 110 unique diseases with a total of 1,025 seed genes (see Additional data files 2 and 5 for details).

#### Neighborhood-weighting decision rule

Given a particular disease and its seed gene set, we quantify the association of each candidate gene *i *with the disease using the following disease association score *S*_*i*_:

(4)

where *w*_*ij *_is the linkage weight connecting gene *i *and seed *j *[41,43]. This score is 0 for genes not connected to any seed. The justifications of the rule selection are described in Additional data file 5.

#### Performance assessment for disease gene prioritization

##### Disease-centric assessment

Disease-centric assessment evaluates an overall prediction performance for each individual disease [41,43]. In particular, for a given disease, first each gene is ranked based on the disease association score (*S*_*i*_; Equation 4). The *S*_*i *_for each seed gene is computed using leave-one-out cross-validation, based on its connectivity to other seeds. Next a ROC curve is generated to assess how well known disease (seed) genes are ranked relative to non-seed genes in the ranked gene list. The ROC curve plots true positive rate (sensitivity) versus false positive rate (1 - specificity) at different *S*_*i *_cutoffs:

(5)

(6)

where TP (true positive) is the number of seed genes above the *S*_*i *_cutoff, FP (false positive) is the number of non-seed genes above the cutoff, TN (true negative) is the number of non-seed genes below the cutoff, and FN (false negative) is the number of seed genes below the cutoff.

Finally, an AUC is calculated for each ROC curve to represent the predictability of a disease. AUC scores range between 0 and 1, with 0.5 and 1.0 indicating random and perfect predictive performance, respectively. Here we treat non-seed genes as non-disease genes, but some of these non-seed genes can be new disease-associated genes. Hence, our performance evaluation is likely an underestimate.

##### Gene-centric assessment

Gene-centric assessment treats each known gene-disease association as a test case [19,23]. For each test case, the task is to assess how well the known disease (seed) gene ranks relative to a background gene set. Gene centric assessment is also carried out using leave-one-out cross-validation. For each test case, the tested seed gene is ranked according to its connectivity to the remaining seed genes (Equation 4), and compared against a background gene set.

To assess the overall performance of a method, we first determine the rank of each tested disease gene among the background gene set within each test case, and then pool together all test cases and compute the fraction of the tested disease genes ranked above various cutoffs. A second commonly used measure is fold enrichment, defined as the average rank of a gene before prioritization divided by the rank after prioritization [19]. For instance, to rank a test disease gene with a background gene set of 100 genes, the average rank before prioritization is 50. If the test disease gene is ranked top 1, then its fold enrichment is 50/1 = 50.

#### Random control

Similar to McGary *et al*. [43], we assemble 100 random gene sets from the FLN for each given disease. Each gene set has the same number of genes as the seed gene set for the given disease. Next we perform the same FLN-based disease gene prioritization and evaluation using these randomly assembled seed sets.

#### Estimate precision for disease gene predictions

To estimate the prediction for disease gene predictions, we first rank the predicted candidate disease genes by their disease association scores (*S*_*i*_; Equation 4). Precision is then estimated at different rank cutoffs.

For each particular disease, each known disease gene (seed) is assigned a disease association score (*S*_*i*_) based on its functional linkage to other seeds. We then rank the seed genes together with other non-seed genes based on their *S*_*i *_scores. At each rank cutoff, we calculate the precision as the fraction of genes above the cutoff that are seed genes. This is likely an underestimate, since some non-seed genes can be new disease-related genes.

For each of the 110 diseases, we provide the precision estimate for its top 100 predicted new candidate genes by plotting precision versus rank cutoff.

### Mutual predictability as an estimate for disease-disease association at the molecular level

Given two diseases, I and II, each of which is associated with a set of known disease genes, we define a mutual predictability score that reflects the degree of association between the two diseases (Figure S7 in Additional data file 5). The mutual predictability score accounts for both the number of genes in common between the two disease gene sets, and the functional links between the two sets in the FLN. In essence, the mutual predictability score evaluates the degree to which genes associated with disease I can be used as seed genes to predict genes associated with disease II (predictability _I-II_), and vice versa (predictability _II-I_). High mutual predictability implies close association between the two disease gene sets in the FLN, suggesting related molecular mechanisms.

To quantify predictability _I-II_, we first use genes associated with disease I as seeds, and rank all other genes based on the *S*_*i *_score (Equation 4). In addition, disease II genes that overlap with the seed genes from disease I are given a *S*_*i *_score of infinity and ranked on the top. Next, we plot a ROC curve of sensitivity versus 1 - specificity on how well the disease II genes are ranked in the sorted gene list. Sensitivity and (1 - specificity) are defined in Equations 5 and 6, where TP is the number of disease II genes above a particular *S*_*i *_cutoff, TN is the number of genes associated with neither disease below the cutoff, FP is the number of genes associated with neither disease above the cutoff, and FN is the number of disease II genes below the cutoff. Finally, we calculate the AUC of the ROC curve (AUC_I-II_) as a measure for predictability _I-II_.

Similarly, we can calculate AUC_II-I _as a measure of predictability_II-I_. The mutual predictability between disease I and II is defined as the geometric mean of AUC_I-II _and AUC_II-I_:

(7)

Since AUC ranges from 0 to 1, the mutual predictability also ranges from 0 to 1.

## Abbreviations

AUC: area under receiver operating characteristic curve; CC: cellular component; DDI: domain-domain interaction; DS: protein domain sharing; FLN: functional linkage network; GO: Gene Ontology; GS: gold standard; GSP: gold standard positive; GSN: gold standard negative; GN: gene neighbour; HPRD: Human Protein Reference Database; KEGG: Kyoto Encyclopaedia of Genes and Genomes; LR: likelihood ratio; LLR: log likelihood ratio; Masspec: Mass Spectrometry; MF: molecular function; MIPs: The Munich Information Center for Protein Sequences; OMIM: Online Mendelian Inheritance in Man Database; PG: phylogenetic profiles; PPI: protein-protein interaction; RefSeq: Reference Sequence Database; ROC: receiver operating characteristic; TexM: text mining; Y2H: yeast two hybrid experiments.

## Authors' contributions

BL designed and implemented the whole computation framework. ESS provided constructive discussions and revised the manuscript. ZH provided constructive discussions. YX monitored the whole framework. CD directed the whole project and is Principal Investigator on the NIH grant that funded the project. All the authors have read and agreed to the manuscript.

## Additional data files

The following additional data are available with the online version of this paper: a text file listing edges in the integrated human functional linkage network (Additional data file 1); a table listing known (seed) disease genes associated with each of the 110 diseases (Additional data file 2); a list of the top 100 predicted candidate disease genes for each of the 110 diseases (Additional data file 3); a plot of precision versus rank cutoff for the top 100 predicted candidate disease genes for each of the 110 diseases (Additional data file 4); supplementary text and figures (Additional data file 5); a list of recently identified disease genes and landmark references dated after January 2007 (Additional data file 6); a list of the 22 predicted obesity genes among the top 100 predicted gene list that overlap with the obesity genes collected from literature by Hanock et al. [58] (Additional data file 7); a list of mutual predictability scores for all the possible disease pairs between the 110 diseases (Additional data file 8); a list of the top 100 disease pairs with the highest mutual predictability scores and the supporting evidence for the association (Additional data file 9); a list of the disease clusters, their disease members, and evidence supporting the associations in Figure S9 of Additional data file 5 and Figure 8a (Additional data file 10); a list of the 53 informative GO terms used to define the gold standard sets for the naïve Bayes integration (Additional data file 11); a list of Pearson correlation coefficients between the 16 features used for the naïve Bayes integration (Additional data file 12).

## Supplementary Material

Additional data file 1 Column 1, Entrez Gene ID for gene A; column 2, Entrez Gene ID for gene B; column 3, functional linkage weight (log likelihood ratio of the naïve Bayes integration). Click here for file

Additional data file 2 Known (seed) disease genes associated with each of the 110 diseases. Click here for file

Additional data file 3 A WinRAR archive composed of a readme.txt file and 110 prediction files for the 110 diseases. For each disease, we list the top 100 ranked new candidate disease genes not included in the disease seed gene set. The description of the prediction files is provided in the readme.txt file. Click here for file

Additional data file 4 Plot of precision versus rank cutoff for the top 100 predicted candidate disease genes for each of the 110 diseases. Click here for file

Additional data file 5 Supplemental methods, supplementary results, supplementary Figures S1 to S9, and supplementary Table S1 to S4. Click here for file

Additional data file 6 Recently identified disease genes and landmark references dated after January 2007. Click here for file

Additional data file 7 The 22 predicted obesity genes among the top 100 predicted gene list that overlap with the obesity genes collected from literature by Hanock *et al*. [58]. Click here for file

Additional data file 8 Mutual predictability scores for all the possible disease pairs between the 110 diseases. Click here for file

Additional data file 9 The top 100 disease pairs with the highest mutual predictability scores and the supporting evidence for the association. Click here for file

Additional data file 10 Disease clusters, their disease members, and evidence supporting the associations in Figure S9 of Additional data file 5 and Figure 8a. Click here for file

Additional data file 11 The 53 informative GO terms used to define the gold standard sets for the naïve Bayes integration. Click here for file

Additional data file 12 Pearson correlation coefficients between the 16 features used for the naïve Bayes integration. Click here for file
