# Supplementary material for: Genome-wide prioritization of disease genes and identification of disease-disease associations from an integrated human functional linkage network
Source: Genome Biol. 2009 Sep 3;10(9):R91. doi: 10.1186/gb-2009-10-9-r91 (PMC2768980; doi:10.1186/gb-2009-10-9-r91)
Supplement: Additional data file 4 — Plot of precision versus rank cutoff for the top 100 predicted candidate disease genes for each of the 110 diseases. [file gb-2009-10-9-r91-S4.pdf]

Precision estimation for the novel predicted candidate disease genes

The procedures for precision estimation of the novel predicted disease genes are provided in Methods section of the main manuscript. Below we provide the plot of precision versus rank cutoff for the top 100 predicted candidate disease genes for each of the 110 diseases. The X axis denotes the rank cutoff; The Y axis denotes the prediction precision, which is calculated as the fraction of the known (seed) disease genes among all the predicted genes above a particular rank cutoff. The 110 diseases are arranged in the alphabetical order.

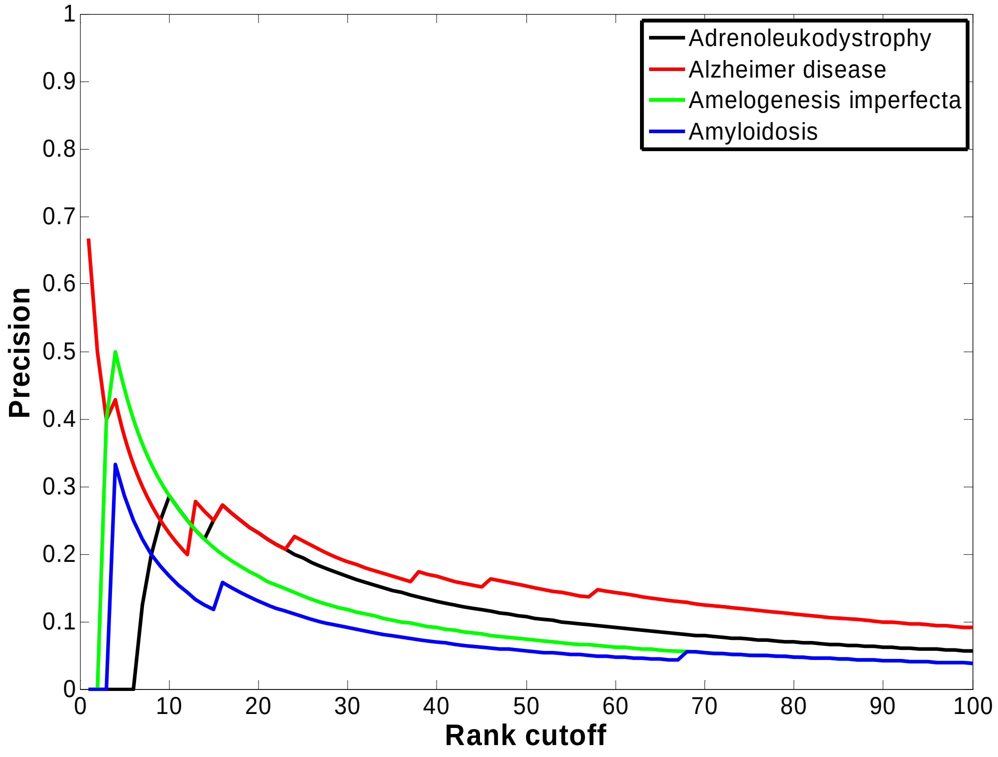

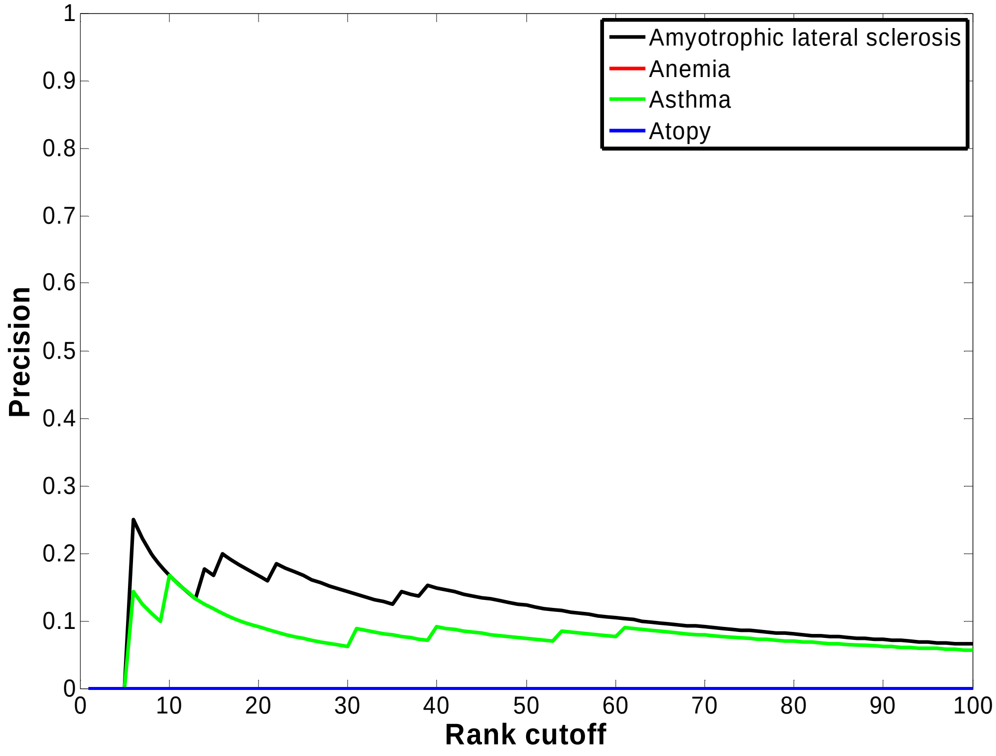
Amyotrophic lateral sclerosis
Anemia
Asthma
Atopy
Precision
Rank cutoff
0
0.1
0.2
0.3
0.4
0.5
0.6
0.7
0.8
0.9
1
0
10
20
30
40
50
60
70
80
90
100

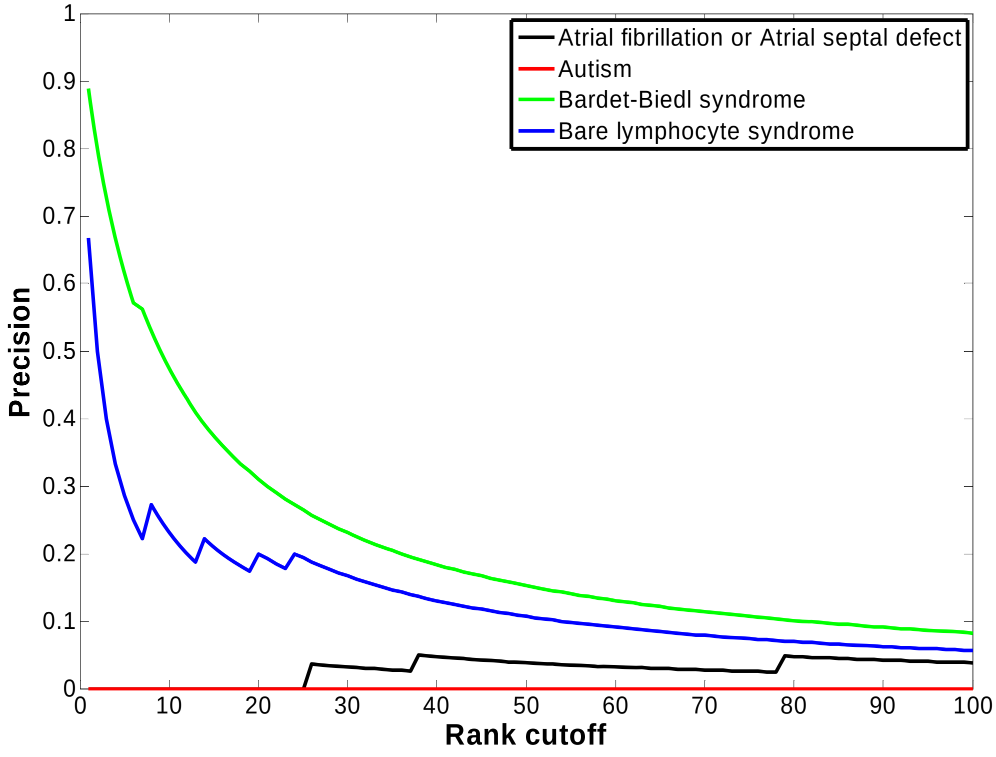
Atrial fibrillation or Atrial septal defect
Autism
Bardet-Biedl syndrome
Bare lymphocyte syndrome
Precision
Rank cutoff
0
0.1
0.2
0.3
0.4
0.5
0.6
0.7
0.8
0.9
1
0
10
20
30
40
50
60
70
80
90
100

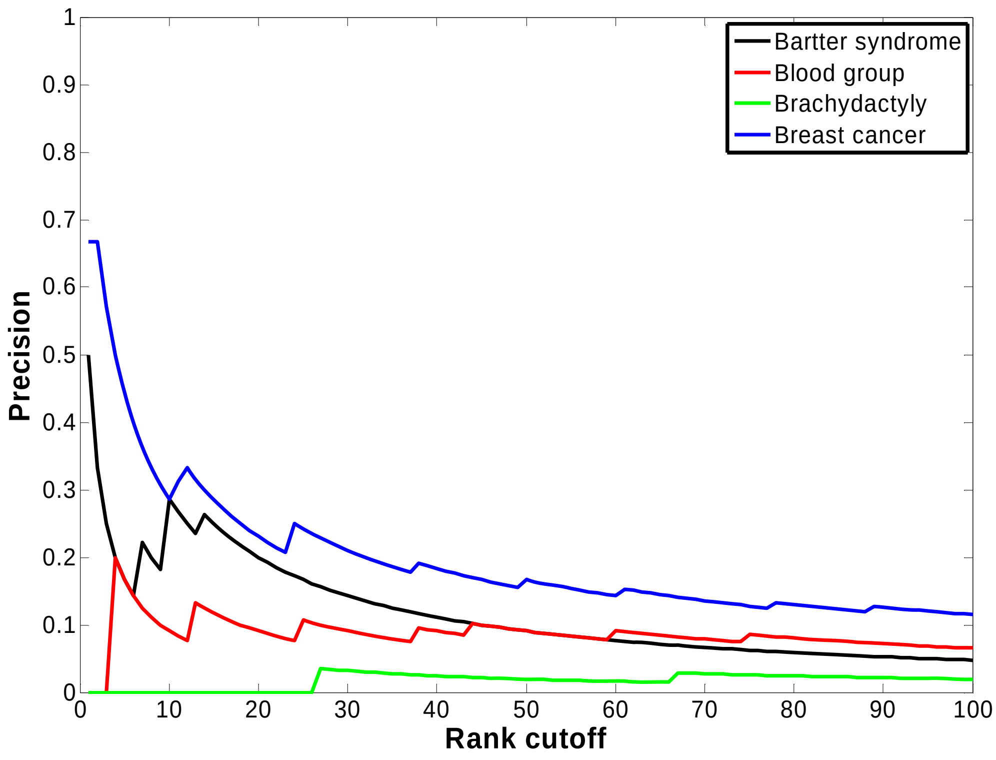

Bartter syndrome
Blood group
Brachydactyly
Breast cancer
Precision
Rank cutoff
0
0.1
0.2
0.3
0.4
0.5
0.6
0.7
0.8
0.9
1
0
10
20
30
40
50
60
70
80
90
100

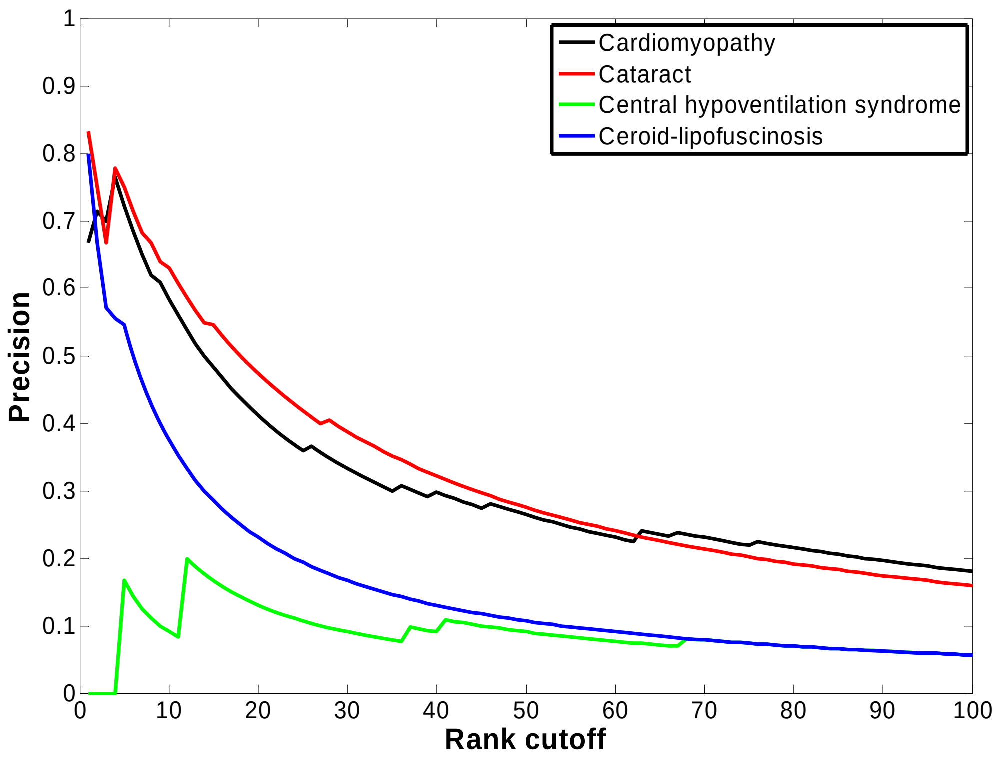

Cardiomyopathy
Cataract
Central hypoventilation syndrome
Ceroid-lipofuscinosis
Precision
Rank cutoff
0
0.1
0.2
0.3
0.4
0.5
0.6
0.7
0.8
0.9
1
0
10
20
30
40
50
60
70
80
90
100

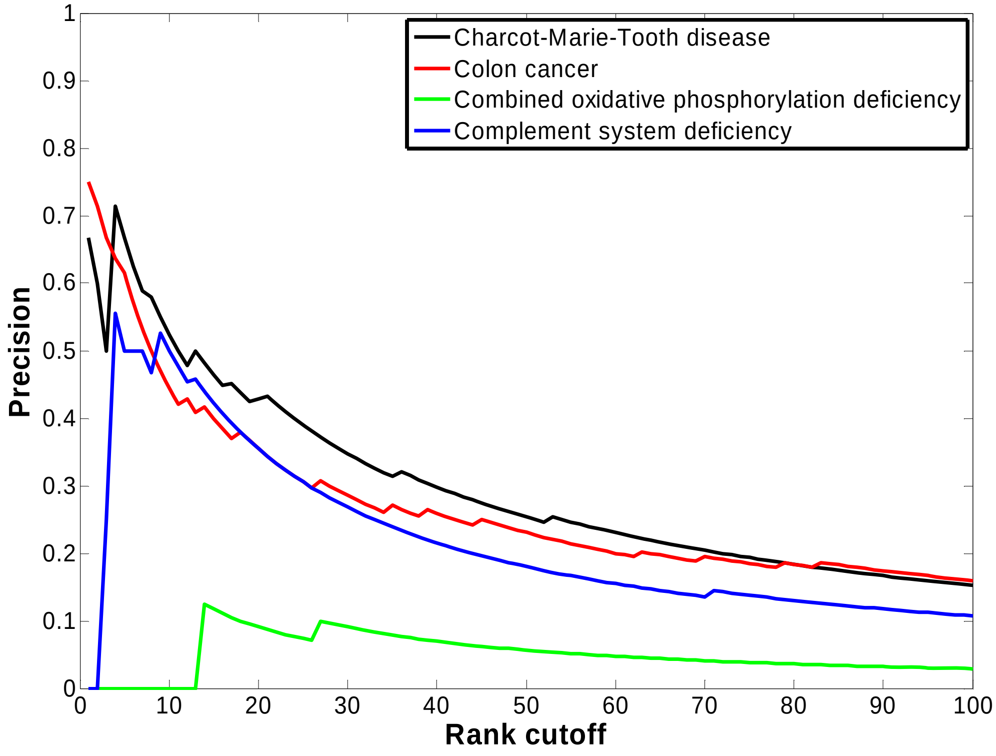
Charcot-Marie-Tooth disease
Colon cancer
Combined oxidative phosphorylation deficiency
Complement system deficiency
Precision
Rank cutoff
0
0.1
0.2
0.3
0.4
0.5
0.6
0.7
0.8
0.9
1
10
20
30
40
50
60
70
80
90
100

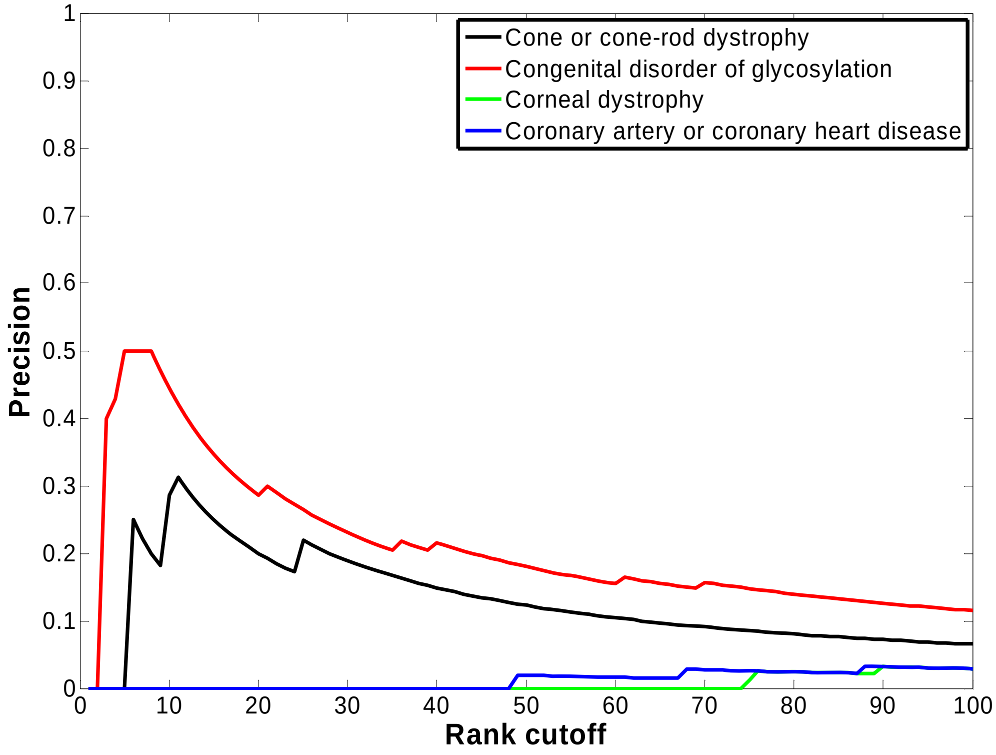
Cone or cone-rod dystrophy
Congenital disorder of glycosylation
Corneal dystrophy
Coronary artery or coronary heart disease
Precision
Rank cutoff
0
0.1
0.2
0.3
0.4
0.5
0.6
0.7
0.8
0.9
1
10
20
30
40
50
60
70
80
90
100

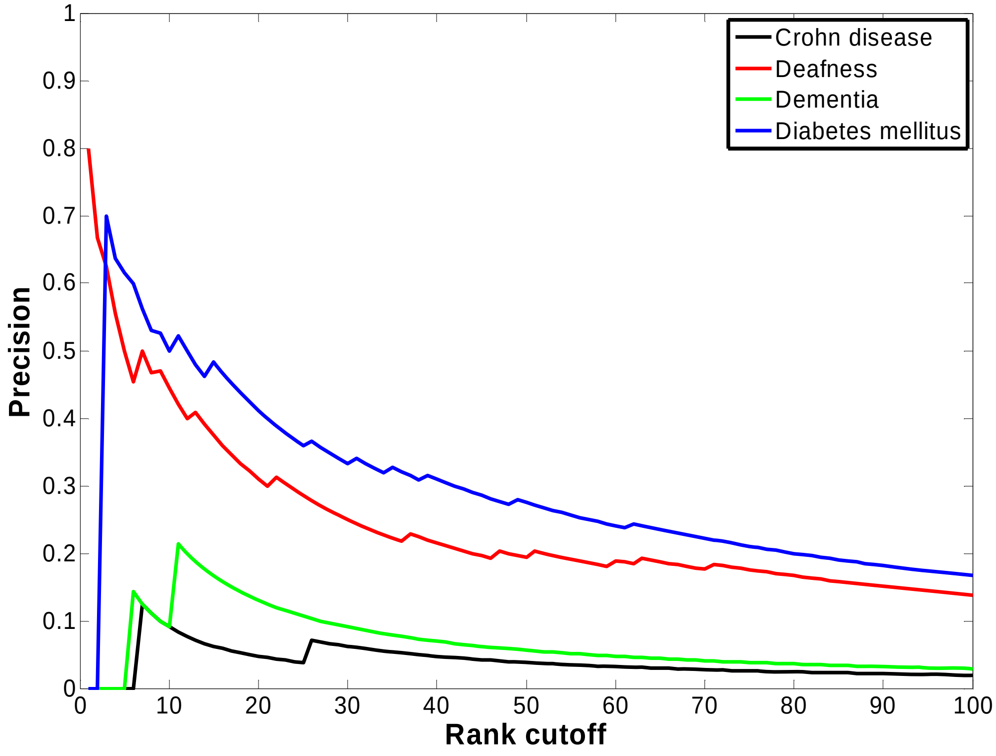
Crohn disease
Deafness
Dementia
Diabetes mellitus
Precision
Rank cutoff
0
0.1
0.2
0.3
0.4
0.5
0.6
0.7
0.8
0.9
1
0
10
20
30
40
50
60
70
80
90
100

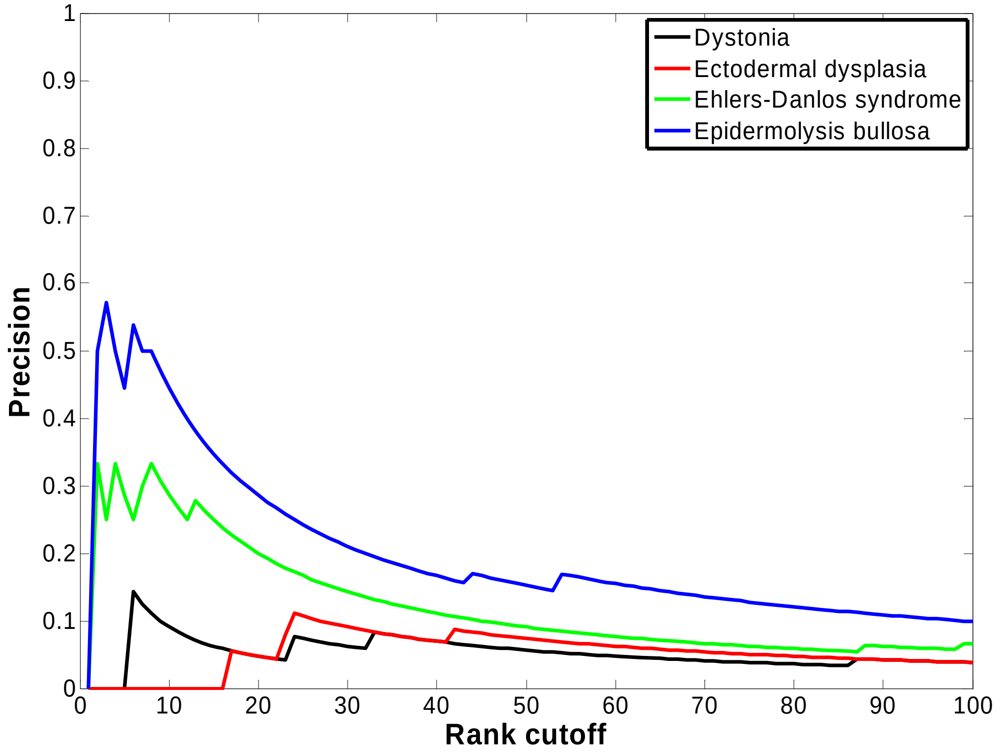
Dystonia
Ectodermal dysplasia
Ehlers-Danlos syndrome
Epidermolysis bullosa
Precision
Rank cutoff
0
0.1
0.2
0.3
0.4
0.5
0.6
0.7
0.8
0.9
1
0
10
20
30
40
50
60
70
80
90
100

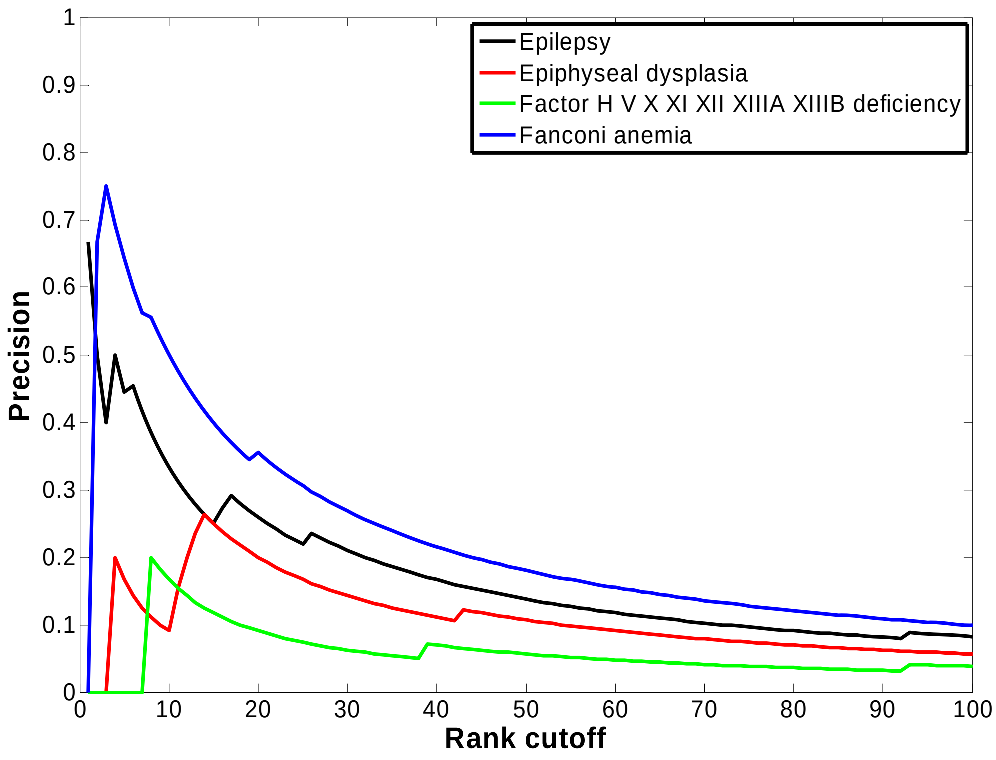
Epilepsy
Epiphyseal dysplasia
Factor H V X XI XII XIIIA XIIIB deficiency
Fanconi anemia
Precision
Rank cutoff
0
0.1
0.2
0.3
0.4
0.5
0.6
0.7
0.8
0.9
1
0
10
20
30
40
50
60
70
80
90
100

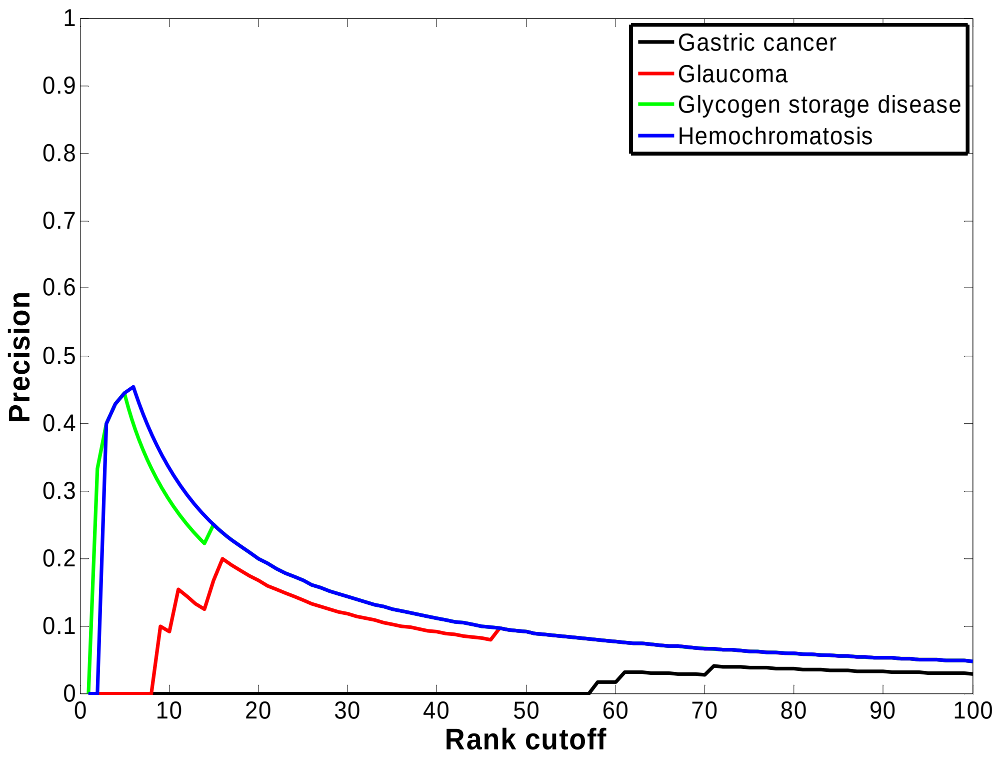
Gastric cancer
Glaucoma
Glycogen storage disease
Hemochromatosis
Precision
Rank cutoff
0
0.1
0.2
0.3
0.4
0.5
0.6
0.7
0.8
0.9
1
0
10
20
30
40
50
60
70
80
90
100

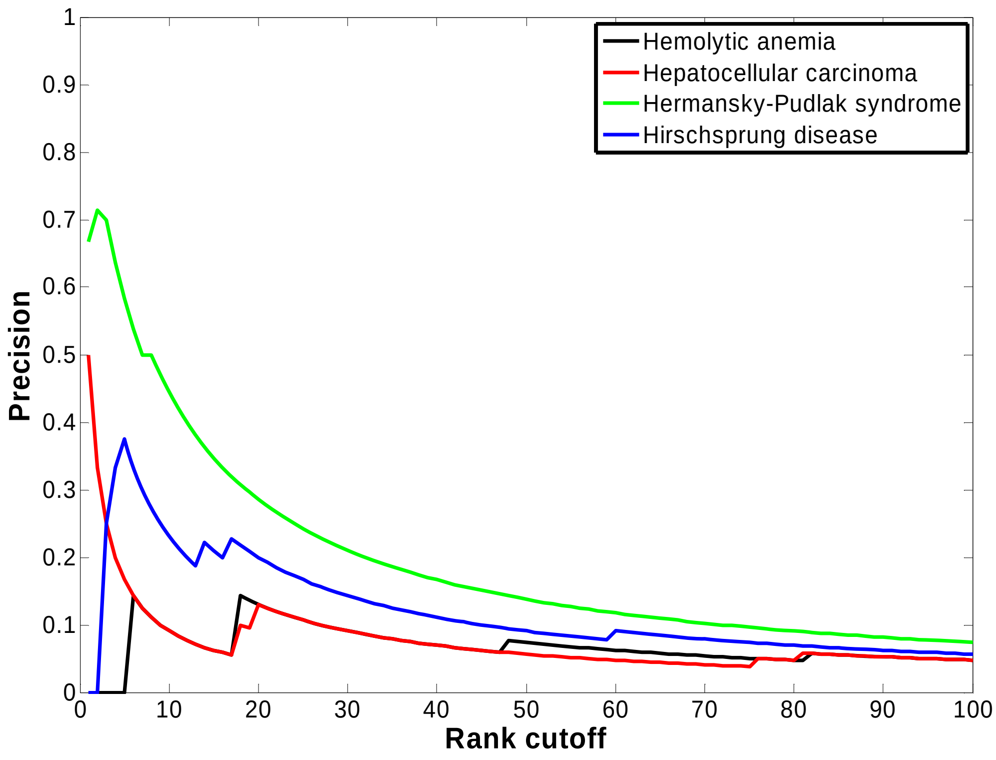
Hemolytic anemia
Hepatocellular carcinoma
Hermansky-Pudlak syndrome
Hirschsprung disease
Precision
Rank cutoff
0
0.1
0.2
0.3
0.4
0.5
0.6
0.7
0.8
0.9
1
0
10
20
30
40
50
60
70
80
90
100

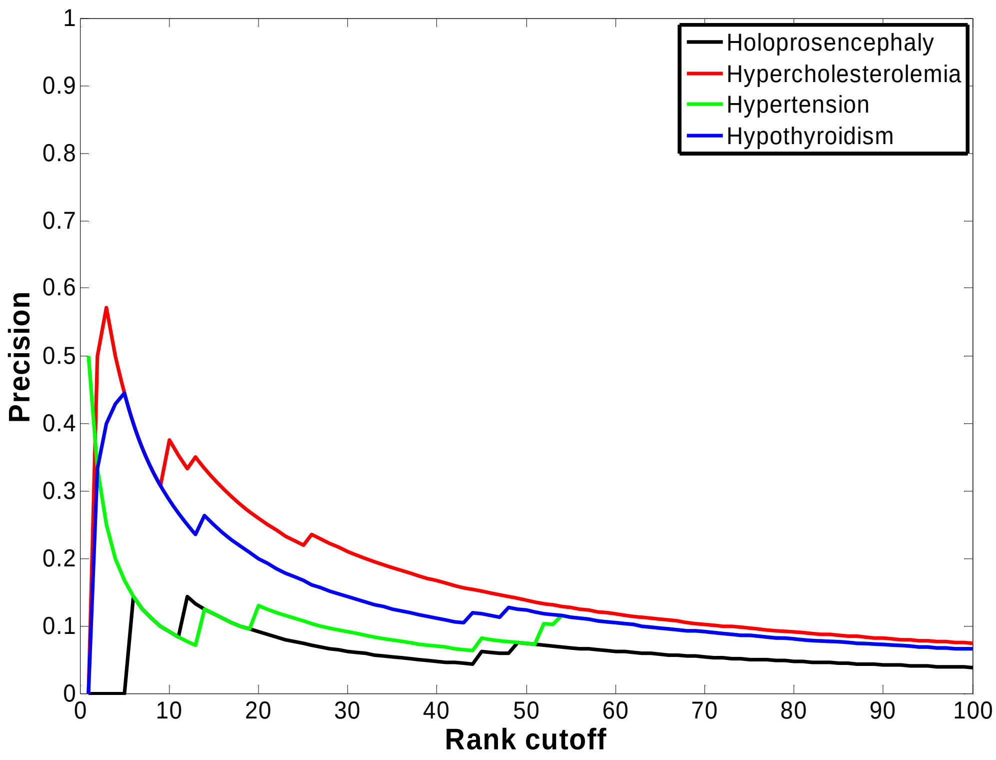
Holoprosencephaly
Hypercholesterolemia
Hypertension
Hypothyroidism
Precision
Rank cutoff
0
0.1
0.2
0.3
0.4
0.5
0.6
0.7
0.8
0.9
1
0
10
20
30
40
50
60
70
80
90
100

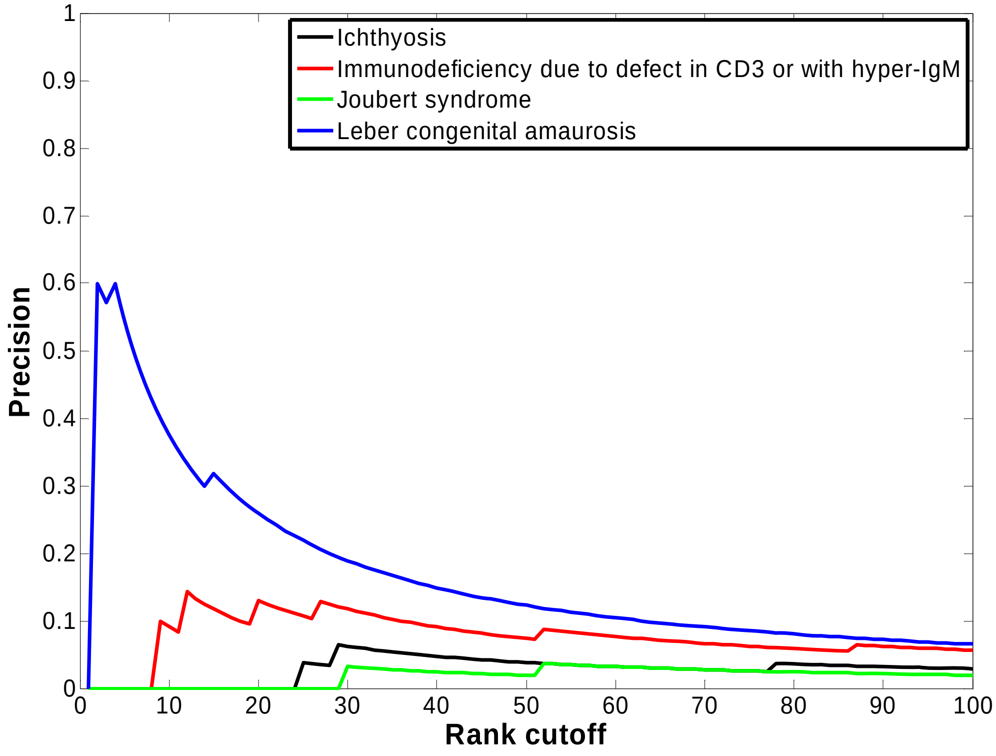
Ichthyosis
Immunodeficiency due to defect in CD3 or with hyper-IgM
Joubert syndrome
Leber congenital amaurosis
Precision
Rank cutoff

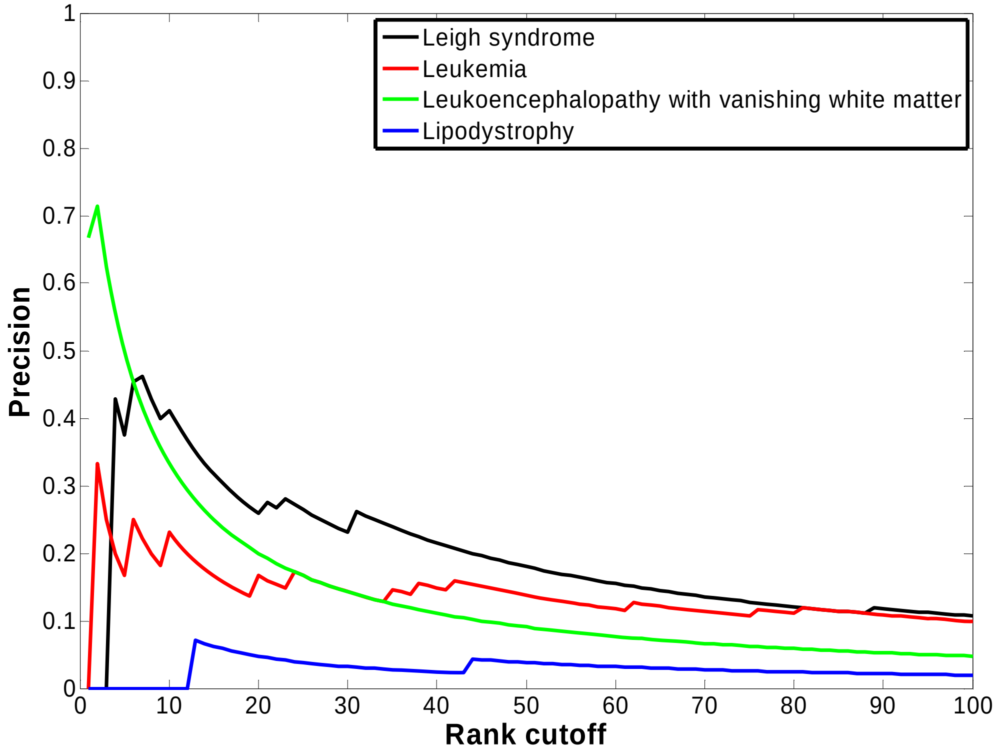
Leigh syndrome
Leukemia
Leukoencephalopathy with vanishing white matter
Lipodystrophy
Precision
Rank cutoff

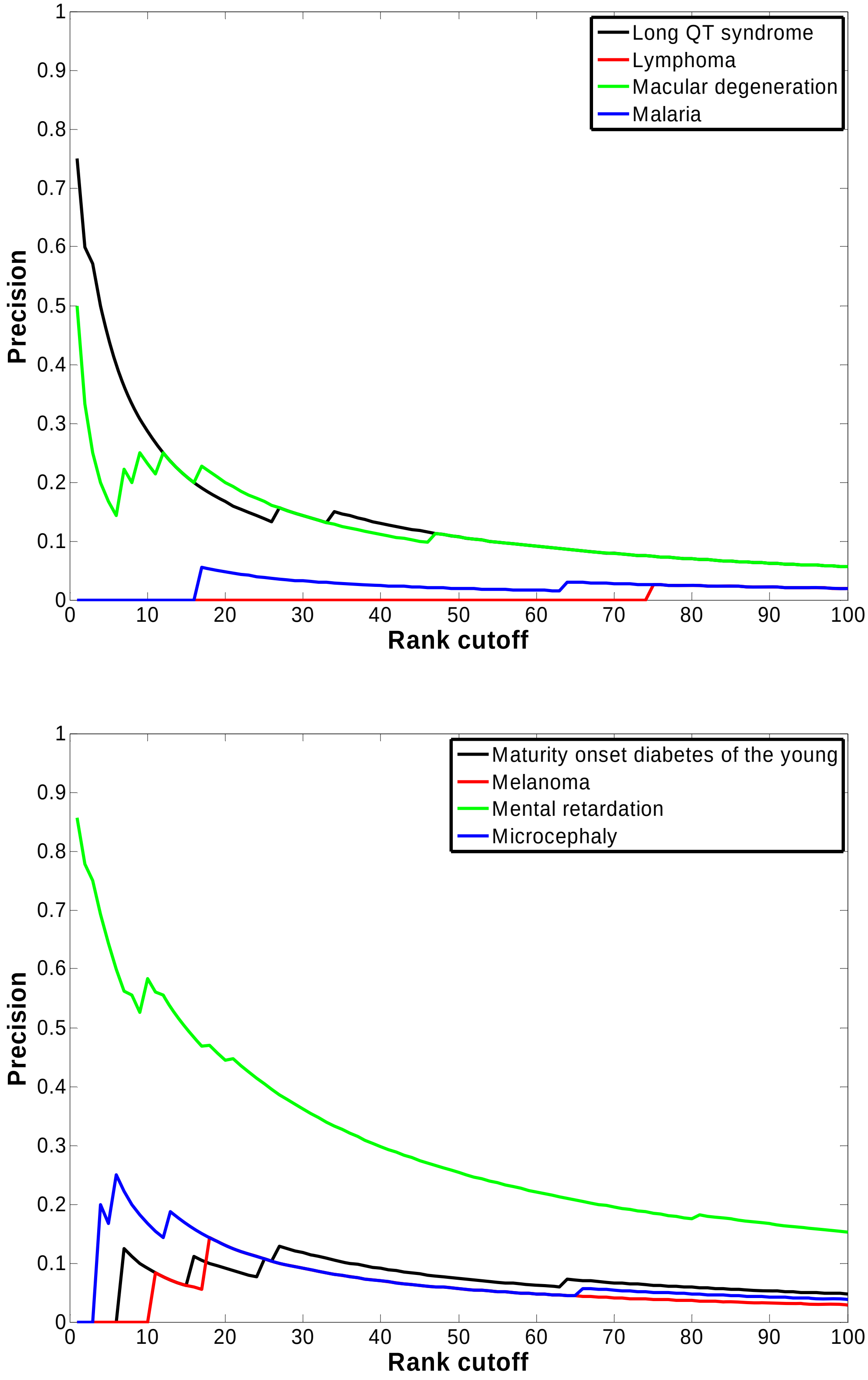
Long QT syndrome
Lymphoma
Macular degeneration
Malaria
Precision
Rank cutoff
Maturity onset diabetes of the young
Melanoma
Mental retardation
Microcephaly
Precision
Rank cutoff

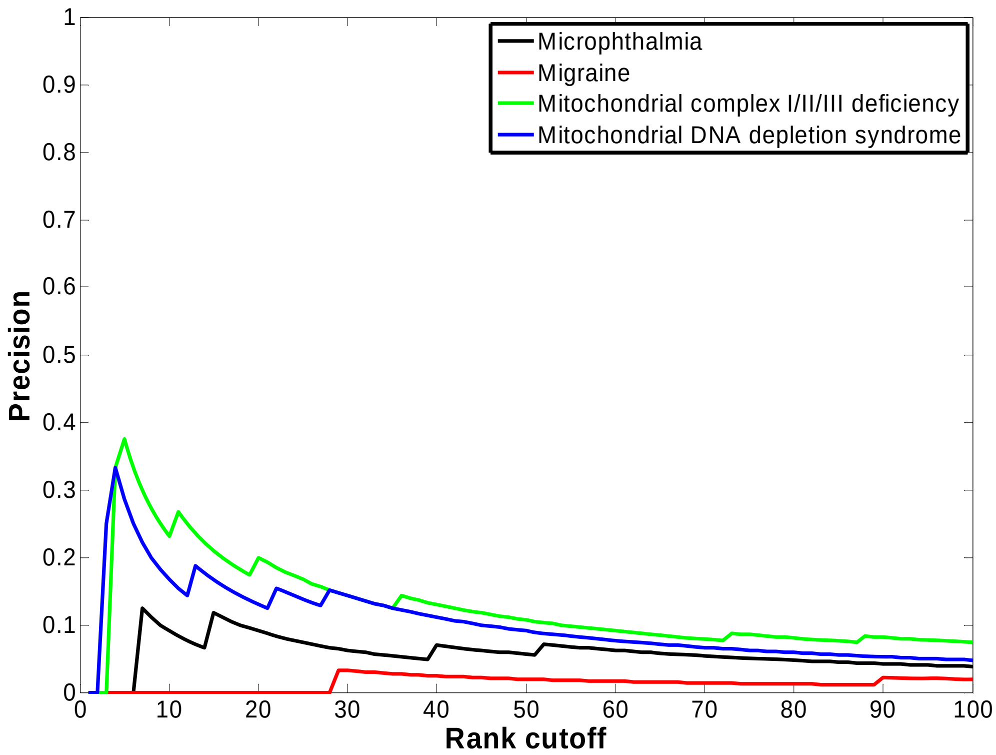
Microphthalmia
Migraine
Mitochondrial complex I/II/III deficiency
Mitochondrial DNA depletion syndrome
Precision
Rank cutoff
0
0.1
0.2
0.3
0.4
0.5
0.6
0.7
0.8
0.9
1
10
20
30
40
50
60
70
80
90
100

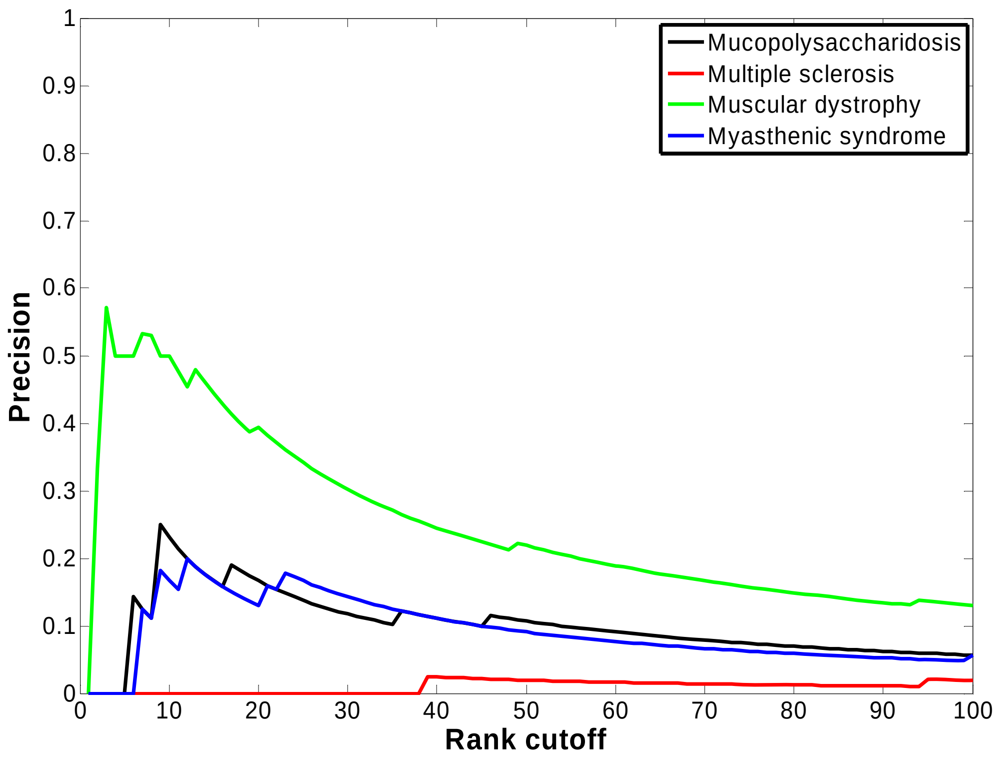
Mucopolysaccharidosis
Multiple sclerosis
Muscular dystrophy
Myasthenic syndrome
Precision
Rank cutoff
0
0.1
0.2
0.3
0.4
0.5
0.6
0.7
0.8
0.9
1
10
20
30
40
50
60
70
80
90
100

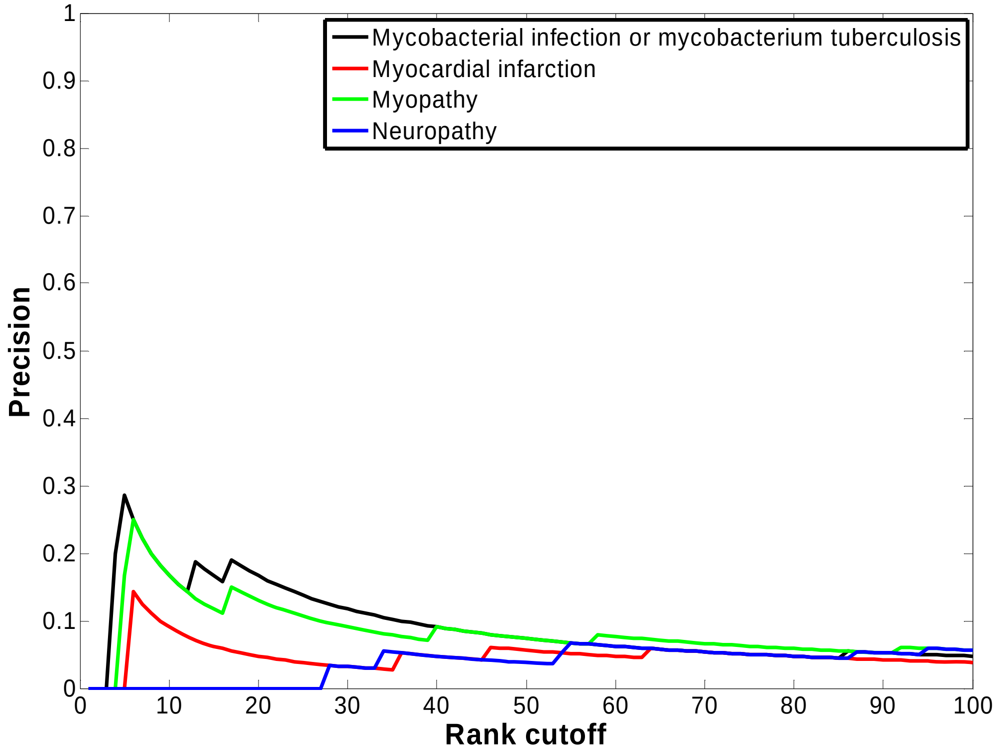

Mycobacterial infection or mycobacterium tuberculosis
Myocardial infarction
Myopathy
Neuropathy
Precision
Rank cutoff
0
0.1
0.2
0.3
0.4
0.5
0.6
0.7
0.8
0.9
1
10
20
30
40
50
60
70
80
90
100

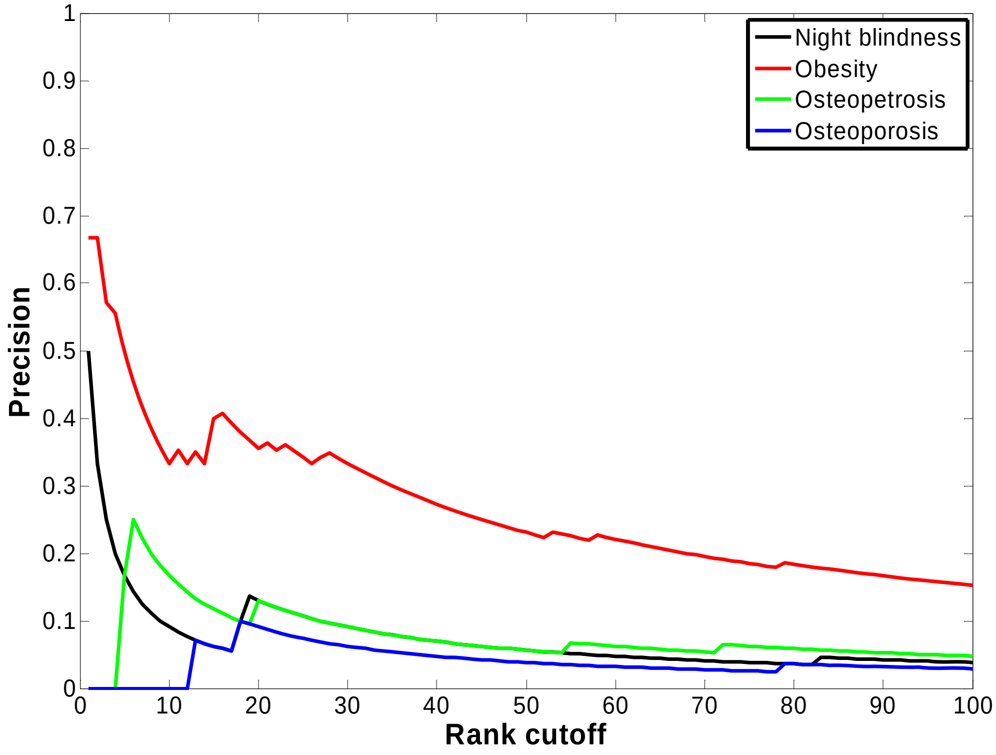

Night blindness
Obesity
Osteopetrosis
Osteoporosis
Precision
Rank cutoff
0
0.1
0.2
0.3
0.4
0.5
0.6
0.7
0.8
0.9
1
10
20
30
40
50
60
70
80
90
100

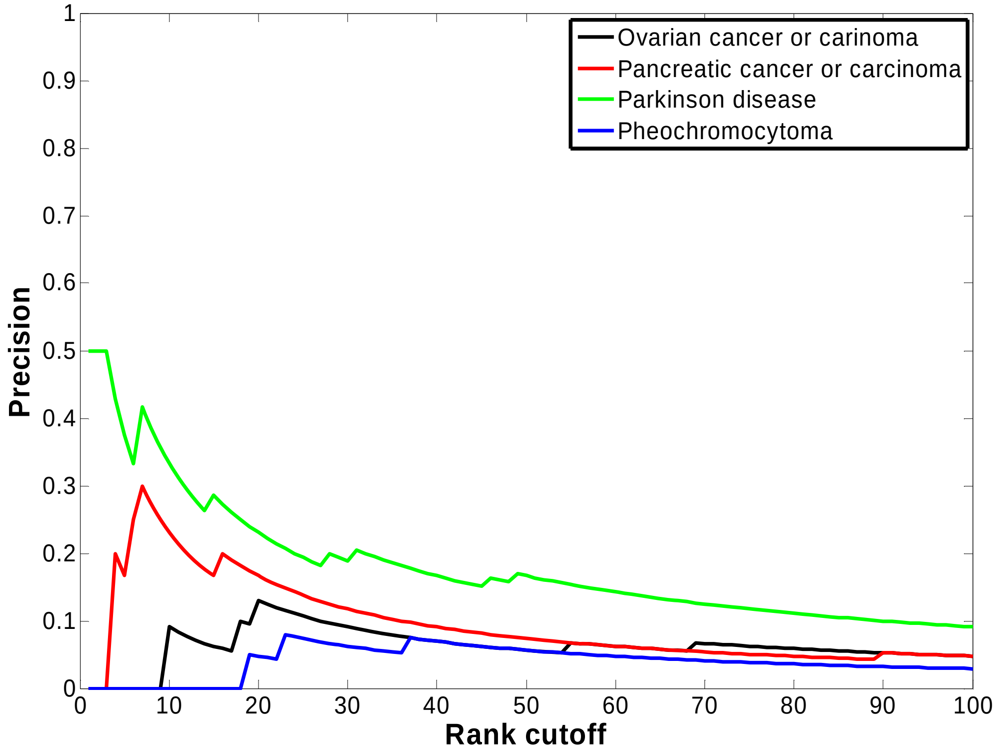
Ovarian cancer or carinoma
Pancreatic cancer or carcinoma
Parkinson disease
Pheochromocytoma
Precision
Rank cutoff
0
0.1
0.2
0.3
0.4
0.5
0.6
0.7
0.8
0.9
1
0
10
20
30
40
50
60
70
80
90
100

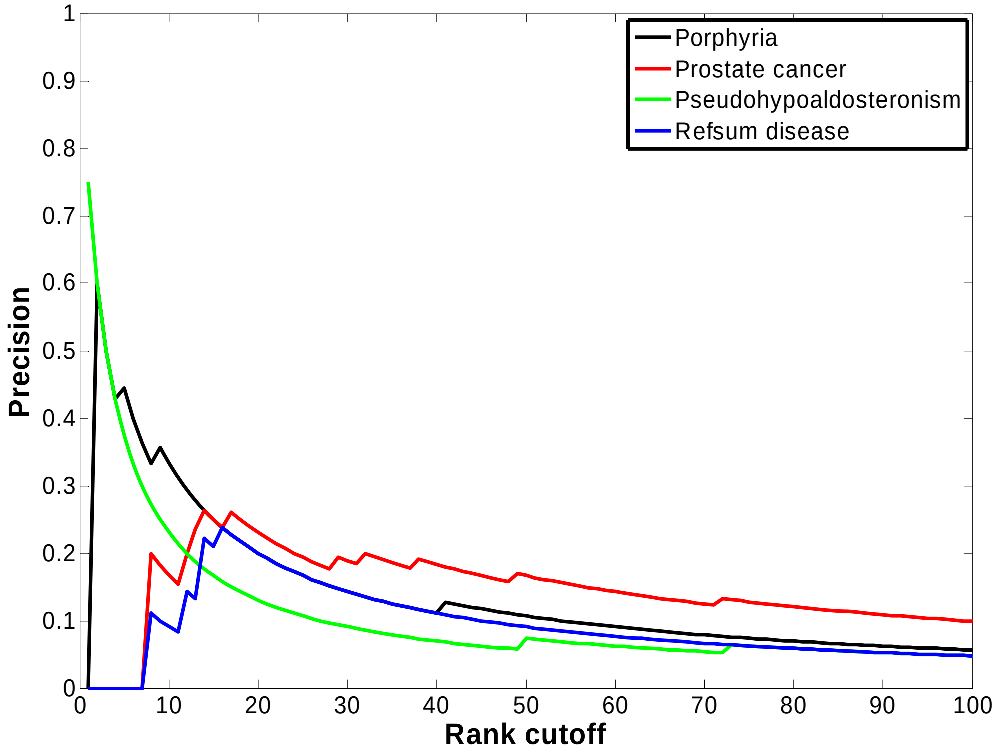
Porphyria
Prostate cancer
Pseudohypoaldosteronism
Refsum disease
Precision
Rank cutoff
0
0.1
0.2
0.3
0.4
0.5
0.6
0.7
0.8
0.9
1
0
10
20
30
40
50
60
70
80
90
100

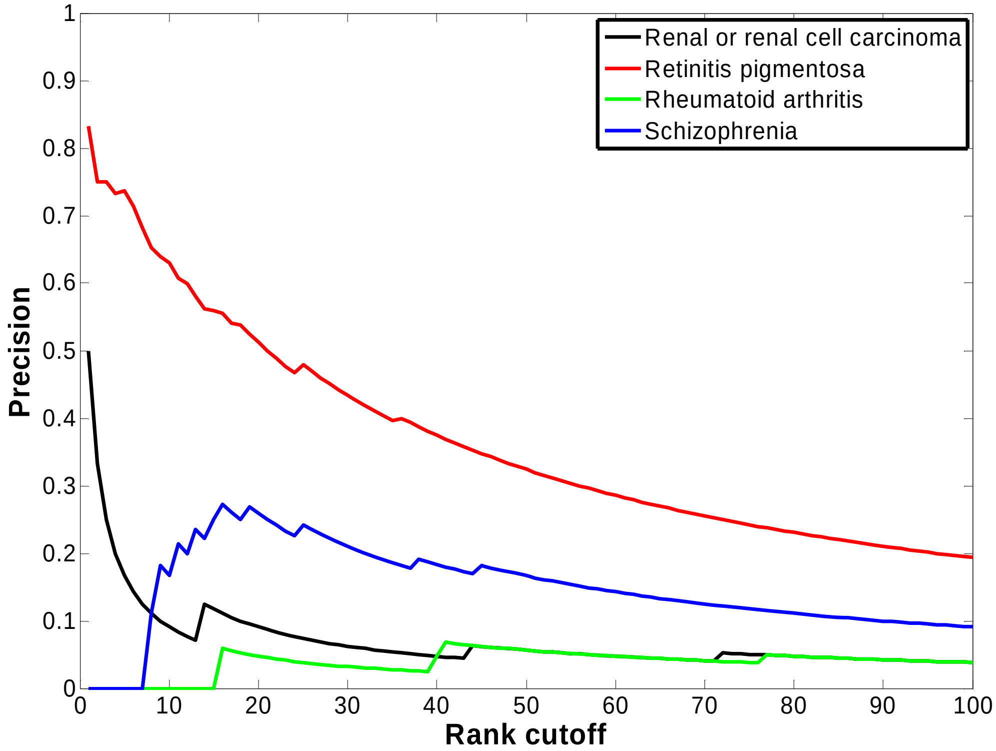
Renal or renal cell carcinoma
Retinitis pigmentosa
Rheumatoid arthritis
Schizophrenia
Precision
Rank cutoff
0
0.1
0.2
0.3
0.4
0.5
0.6
0.7
0.8
0.9
1
10
20
30
40
50
60
70
80
90
100

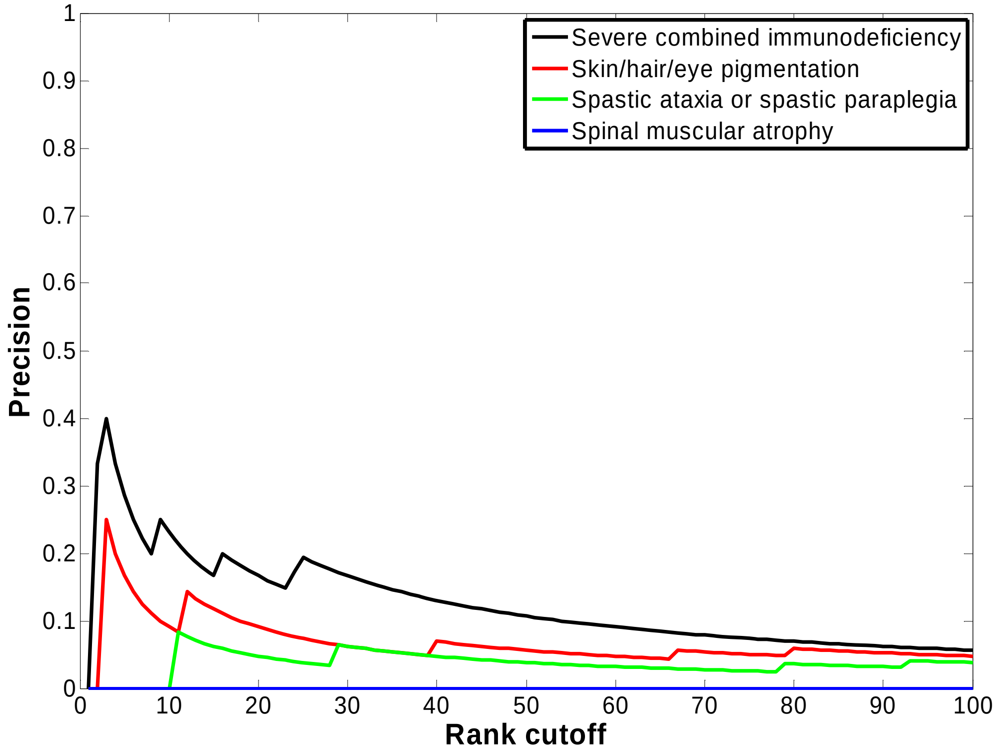
Severe combined immunodeficiency
Skin/hair/eye pigmentation
Spastic ataxia or spastic paraplegia
Spinal muscular atrophy
Precision
Rank cutoff
0
0.1
0.2
0.3
0.4
0.5
0.6
0.7
0.8
0.9
1
10
20
30
40
50
60
70
80
90
100

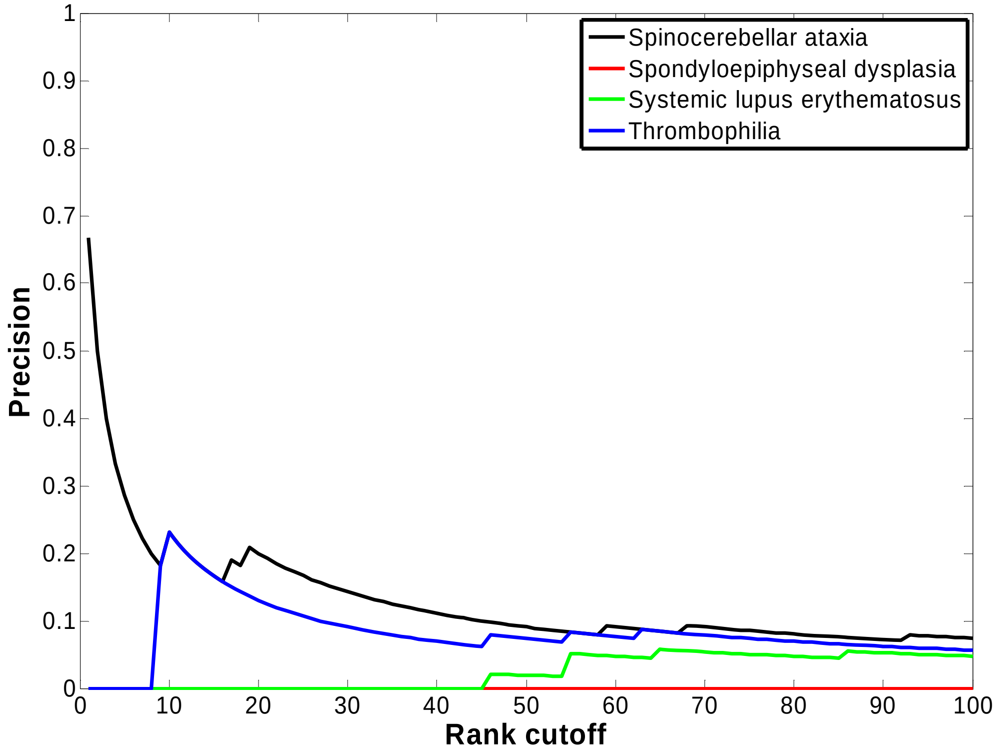

Spinocerebellar ataxia
Spondyloepiphyseal dysplasia
Systemic lupus erythematosus
Thrombophilia
Precision
Rank cutoff

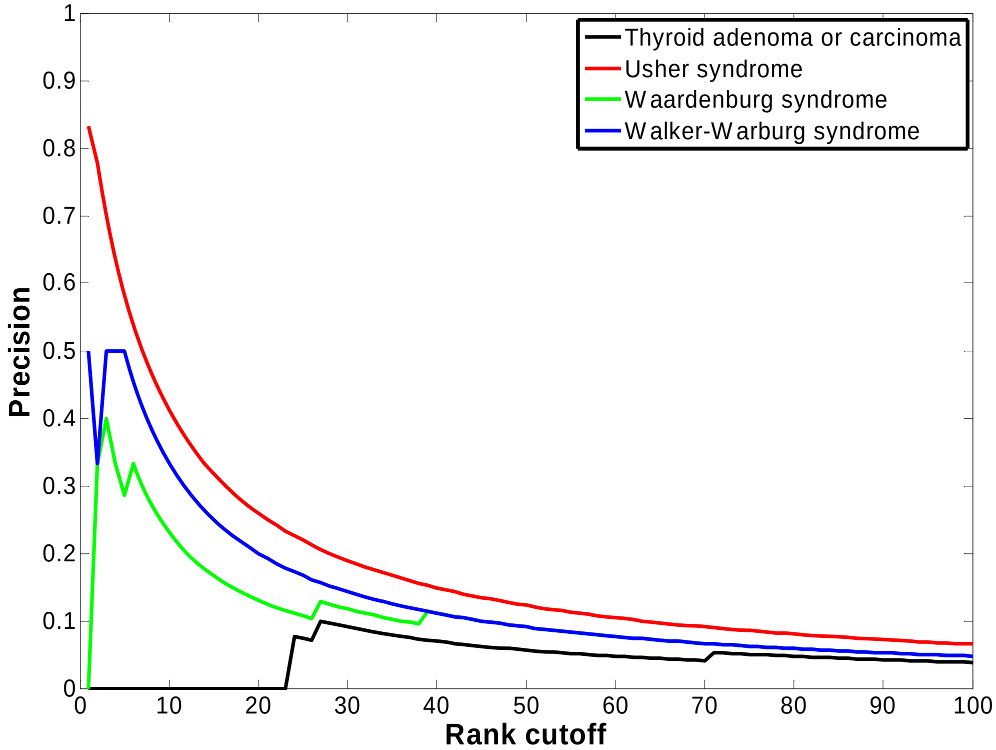

Thyroid adenoma or carcinoma
Usher syndrome
Waardenburg syndrome
Walker-Warburg syndrome
Precision
Rank cutoff

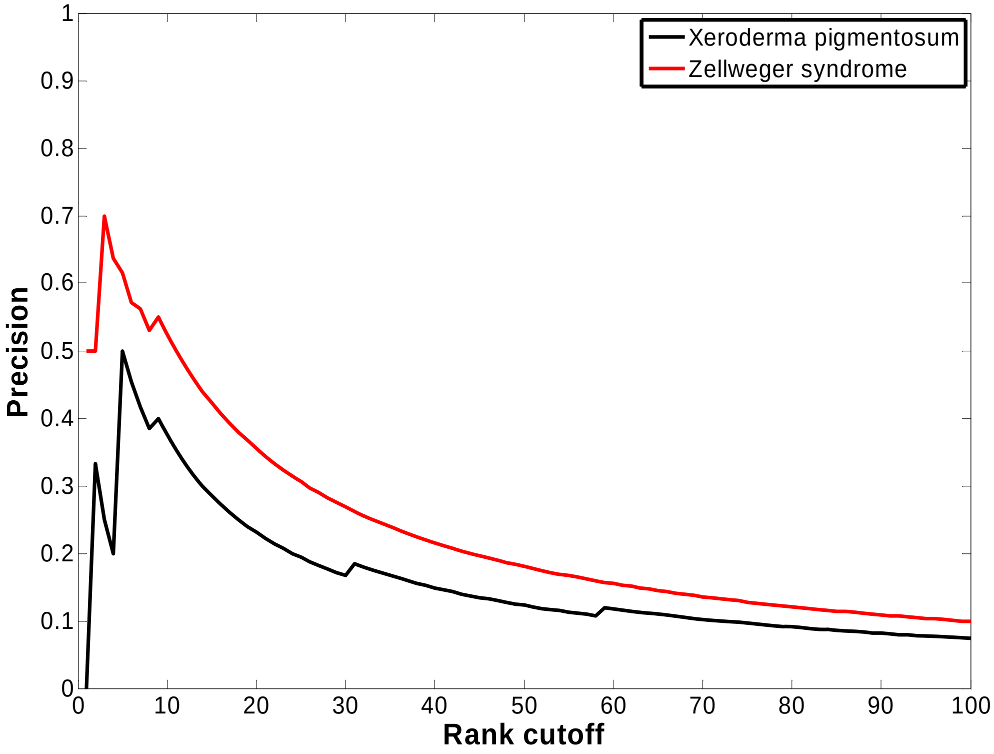

Xeroderma pigmentosum
Zellweger syndrome
Precision
Rank cutoff
0
0.1
0.2
0.3
0.4
0.5
0.6
0.7
0.8
0.9
1
0
10
20
30
40
50
60
70
80
90
100
