# Supplementary material for: Genome-wide prioritization of disease genes and identification of disease-disease associations from an integrated human functional linkage network
Source: Genome Biol. 2009 Sep 3;10(9):R91. doi: 10.1186/gb-2009-10-9-r91 (PMC2768980; doi:10.1186/gb-2009-10-9-r91)
Supplement: Additional data file 5 — Supplemental methods, supplementary results, supplementary Figures S1 to S9, and supplementary Table S1 to S4. [file gb-2009-10-9-r91-S5.pdf]

Supplemental text and figures

1. Details for naïve Bayes integration

1.1. Gold standard datasets

We define gene pairs sharing the same biological process term in Gene Ontology (GO) as being functionally associated. Since GO terms are organized hierarchically, according to different levels of specificity, we need to choose a specificity level. Similar to other research groups [1-3], we count the total number of genes annotated with a GO term to measure that term's specificity, with low counts corresponding to high specificity GO terms and high counts corresponding to low specificity terms [1-3]. To select GO terms with the appropriate level of specificity, we adopt the definition of an informative GO term defined by Zhou *et al*. [1, 3]. Specifically, informative GO terms need to satisfy two criteria: (i) at least 175 genes are annotated with the term, and (ii) none of its descendant terms are used to annotate over 175 genes. The files for mapping genes to GO terms were downloaded from Entrez Gene [4], and we only consider annotations with non-IEA evidence code [5]. Based on the above criteria, a total of 54 informative GO terms are selected.

We define gene pairs sharing one or more informative biological-process GO terms as the *gold standard positive (GSP)* set. To minimize bias, the most populous informative GO term "GO:0048513" ("organ development") is excluded from the GSP set (it is associated with ~14% of the GSP if included, compared to <6% of the GSP for any of the other 53 informative GO terms [6]). A list of the 53 GO terms that we include is provided in Additional File 13.

Our *gold standard negative (GSN)* set is defined as the collection of gene pairs that are annotated by the 53 GO informative terms but do not share common GO biological-process terms except for the root term.

In addition, we also construct the gold standard set using KEGG pathways instead of GO terms. The integration performance using KEGG is similar to that using GO.

1.2 Features and scoring

(1) Curated protein-protein interaction (curated PPI)

Curated human protein-protein interaction are downloaded from HPRD, BIND, BIOGRID, INACT, MIPS, DIPS, and MINT [7-13]. To differentiate with high-throughput PPI, large-scale yeast two-hybrid data and mass spectrometry data are excluded. We use a binary score to denote the presence or absence of an interaction. All these databases retrieve their data entries from published literature. To minimize redundancy, we combine these 7 datasets as one feature as described in Section 1.4.

(2) High-throughput Yeast two-hybrid (Y2H)

We download Y2H data from Rual *et al*'s datasets [14]. We also use a binary score to denote the presence or absence of an interaction.

(3) Large-scale mass spectrometry (MassSpec)

Our mass spectrometry data are from Ewing et al [15]. We also use a binary score to denote whether two proteins occur in the same protein complex. Note: The high-throughput Y2H experiment and the large-scale mass spectrometry are performed by independent groups, so we treat them as two separate features.

(4) PPI inferred from domain-domain interaction (DDI)

Protein-protein physical interactions (PPI) involve interactions between protein domains. Therefore new PPI's could be predicted by identifying pairs of protein domains enriched in known interacted protein pairs [16, 17]. Two studies have identified enriched domain pairs by mining the HPRD database and the identified domain pairs were measured by confidence scores [16, 17].

We download these domain pairs from both studies and then retrieve the protein pairs containing these domain pairs using InterPro database [18]. Each retrieved protein pair is given the same confidence score as the corresponding domain pair. Since both studies identified the interacted domain pairs by mining the HPRD database, to minimize redundancy we combine the two resulting datasets as one feature as described in Section 1.4. In addition, we also exclude protein pairs having been incorporated in the curated PPI since their interactions have been counted.

(5) Text mining (TexM)

Text mining is also an important source to retrieve functionally associated gene pairs by searching for co-ocurrence of gene names in PubMed abstracts [19]. We download human text mining data from String database with each gene pair associated with a corresponding text mining score [19].

(6) Correlated gene expression (Co-exp)

Correlated gene expression from gene expression microarray experiments is also included as a feature to predict functionally associated gene pairs. We include three expression-correlation studies [19-22]. Each of them incorporated multiple microarray datasets and identified gene pairs with correlated gene expression profiles. We refer to these three studies as Tmm [20], Canada [21], and String [19, 22], respectively.

For Tmm and String, we directly download the co-expressed gene pairs and their co-expression scores. For Canada, we use the high-confidence human expression set and treat the data set as binary, 1 for presence of high-confidence correlation and 0 for absence of high-confidence correlation.

To minimize redundancy, the above three co-expression datasets are combined as one feature as described in Section 1.4.

(7) Phylogenetic profile (PG)

The presence and absence of a protein across a set of genomes can be represented by a binary string, a phylogenetic profile [19, 23, 24]. Genes with sufficiently similar profiles tend to be functionally related [19, 23, 24]. Gene pairs with correlated phylogenetic profiles and their associated correlation scores are downloaded from String database [19, 23, 24].

(8) Gene neighbor (GN)

If two genes are found to be chromosomal neighbors in several different genomes, a functional linkage can be inferred between the proteins they encode [19, 23, 24]. Gene pairs identified by gene neighbor method and their associated interaction scores are downloaded from string database [19, 23, 24].

(9) Gene fusion (Fusion)

Some protein pairs with similar functions fuse into different domains of one single protein in other species [19, 23, 24]. Gene pairs with domain fusions and their scores are downloaded from String database [19, 23, 24].

(10) Functional associations inferred from model organisms (Yeast, Worm, Fly, and Mouse-rat)

Previous studies indicated that gene-gene functional associations in worm can be predicted by mapping the functional associations in other model organisms via gene orthology [6]. Such conserved functional associations are defined as 'associalogs' [6]. Similarly, we retrieve functionally associated gene pairs from yeast [25], worm [6], fly [19], mouse [19] and rat [19] and then map them into their human associalogs using INPARANOID databases [26].

For yeast, the human associalogs are mapped from yeast functional genomic data, including co-citation, co-expression, genetic interaction, co-complex, curated PPI, interaction inferred from protein tertiary structure, and high-throughput yeast 2 hybrid, which are all downloaded from [25].

For worm, the human associalogs are mapped from worm functional genomic data, including co-citation, co-expression, genetic interaction, and worm protein interactome, which are all downloaded from [6].

For each of fly, mouse and rat, the corresponding human associalogs are mapped from corresponding organism's functional genomic data, including co-expression, protein-protein interaction, text mining, which are downloaded from String database [19].

Associalogs from each model organism encode one single feature. We evaluate the correlation of associalogs mapped from any two model organisms. Not surprisingly, we find strong correlation (Pearson correlation coefficient = 0.645) between mouse and rat, since they are very close in the evolutionary tree. To minimize redundancy, we combine associalogs from mouse and rat into one feature as described in Section 1.4. These associalog features are referred as "Yeast", "Worm", "Fly", and "Mouse-rat", respectively.

(11) Sharing molecular-function terms in Gene Ontology (MF)

Two genes sharing the same molecular function GO term [27] are more likely to belong to the same biological process (functionally linked) [28]. Furthermore, genes sharing more specific molecular function terms should be more likely to belong to the same biological process than genes sharing general terms. We download human molecular function annotations from Entrez Gene (Nov. 2007) [4]. As a measure of functional association for two genes with one or more shared GO terms, every shared GO term is examined for the count of the genes annotated with that term and the smallest count is used as the score [16]. A lower score corresponds to higher degree of functional association.

(12) Sharing cellular-component GO terms (CC)

Cellular localization information can be described by GO cellular-component terms [27]. Genes sharing cellular localizations tend to functionally associated. We downloaded human cellular component annotations from Entrez Gene (Nov. 2007) [4]. The scoring scheme is the same as the feature of "Sharing molecular-function GO terms".

(13) Protein domain sharing (DS)

Proteins containing the same protein domains tend to have similar function [29, 30]. To retrieve protein domain information, we use InterPro database, which is a database of protein families, domains, repeats, and sites [18]. We refer these protein families, domains, repeats, and sites as InterPro terms. Similar to GO terms, there are also hierarchical organizations among these InterPro terms. Therefore we use the same scoring scheme as the feature of "Sharing molecular-function GO terms".

1.3 Overlap among Y2H, MassSpect , and curated PPI

Three PPI sources were included as input features for naïve Bayes integration: (i) Rual et al. Y2H dataset, (ii) Ewing et al. MassSpect interaction dataset, and (iii) the union of seven curated PPI databases (HPRD, BIND, BIOGRID, INTACT, MIPS, DIPS, and MINT). All seven curated PPI databases have a PubMed ID field describing the literature source of a PPI record. By examining the PubMed ID field of these databases, we found that the Rual et al. Y2H dataset and the Ewing et al. APMS interaction dataset are indeed included in some of the curated databases. To fix this problem of redundancy, we removed Rual et al. Y2H dataset and Ewing et al. APMS interaction dataset from the curated databases, based on the corresponding PubMed ID fields.

We compared the overlap between the curated PPI, Rual et al. Y2H, and Ewing et al. APMS datasets. The comparison results reveal small overlaps among the three PPI sources (see table S1, S2, and S3). It is still possible for a protein-protein interaction to be included in more than one PPI source; however, this is not

because the same interaction from the same literature reference is counted multiple times, but rather because the same interaction has been detected in multiple independent experiments from different literature references. These cases can be properly handled by naïve Bayes integration.eing redundant and treat them as 3 features.

1.4 Calculation of likelihood ratios (LRs)

(1) Features with binary scores

$$LR(f_i = 1) = \frac{P(f_i = 1 \mid I)}{P(f_i = 1 \mid \sim I)} \qquad \text{(S1)}$$

$$LR(f_i = 0) = \frac{P(f_i = 0 \mid I)}{P(f_i = 0 \mid \sim I)} \qquad \text{(S2)}$$

Where I and $\sim I$ denote belonging to gold standard positive set and gold standard negative set, respectively; and f_i denotes feature i.

(2) Features with non-binary scores

For a feature i (f_i) with continuous values, we bin the scores into consecutive intervals and then calculate a LR for each individual bin. We use $start_{ij}$ and end_{ij} to denote the start value and end value for interval j of feature f_i ($start_{ij} < end_{ij}$), respectively.

$$LR(f_i\_bin_j) = \frac{P(start_{ij} \le f_i < end_{ij} \mid I)}{P(start_{ij} \le f_i < end_{ij} \mid \sim I)} \qquad \text{(S3)}$$

Where I and $\sim I$ denote belonging to gold standard positive set and gold standard negative set, respectively; and f_i denotes feature i; $f_i\_bin_j$ denotes the *jth* bin for f_i.

Gene pairs with missing values are put into a separate bin for LR calculation.

(3) LR calculation for associalogs mapped from model organisms' functional associations is similar to those used by Zhong *et al* and Lee *et al* [6, 31]. Below we use yeast as an example to show the calculation procedures.

(i) For each type of functional genomics data in yeast, identify the human orthologs according to INPARANOID database and give them the same association scores as in yeast [26].

(ii) Treat each type of functional genomics data as a single feature and calculate their LR as described above based on the human gold standard set.

(iii) Perform naïve Bayes integration for these yeast based features by multiplying their LRs (equation 2 of the main manuscript). The resulting integrated LR is used as one overall feature of yeast associalogs for the final integration.

1.5 Combination of two or more dependent datasets or features as one single feature

First, for each dataset or each feature, an individual LR is calculated independently. Next, the maximum LR among these individual LRs is used as the final LR for each gene pair [16, 31].

1.6 Integration procedures

Step 1) Calculate LRs for the associalogs of yeast, worm, fly, mouse and rat. The mouse associalogs and rat associalogs are combined as one feature. Each of the rest organisms is treated as a single associalog feature.

Step 2) Calculate LR for each of the rest non-associalog features listed in Section 1.2.

Step 3) After Step 2, a total of 16 features are generated (Table 1 of the main manuscript) and the LR for each feature is calculated. Perform the final naïve Bayes integration using these 16 features (Equation 3 of the main manuscript).

2. Seed genes for FLN-based disease gene prioritization

The seed disease genes are obtained from the Online Mendelian Inheritance in Man (OMIM) database, a compendium of human disease genes and phenotypes [32]. The disease-gene associations are downloaded from the Morbid Map (April 2008) [32]. To obtain highly reliable seeds, we select only entries with the "(3)" tag, which indicates "mapping of a wildtype gene combined with demonstration of a mutation in that gene in association with the disorder" [32, 33]. Next the subtypes of a single disease are merged into one unique disorder entry according to the merging files provided by Gol *et al.* (Supporting Information Table 1 in Goh et al's paper [33]). For example, "Alport syndrome, 301050 (3)" and "Alport syndrome, autosomal recessive, 203780 (3)" are merged as "Alport syndrome". Each disease is assigned a disease ID.

3. Selection of the Neighborhood weighting rule

We perform network based disease gene prioritization in two steps: (i) build a functional linkage gene network as input network by integrating multiple data sources, and (ii) apply an appropriate decision rule to the input gene network to rank candidate genes based on their connection to the seed disease genes. The choice of the optimal decision rule is determined by the nature of the input gene network.

Our choice of the neighborhood weighting (NW) rule is based on the unique properties of our FLN. Derived from extensive data integration (16 genomic features and over 30 sub-features), our FLN is a very dense and high-coverage gene network (over 21,000 genes and over 22,000,000 edges), and each gene has over 2,000 neighbors on average. Edges in our FLN are properly weighted by naïve Bayes, and the linkage weight is proportional to the strength of the functional association between a linked gene pair (Figure 2). The NW rule, which ranks each gene in the network according to the sum of the weights of its links to the known disease (seed) genes (Equation 4, see Methods), takes advantage of linkage weights and the availability of direct measures of functional associations between each seed gene and thousands of its neighboring genes to successfully rank about 12,000 genes for each disease.

The NW rule is simple, yet already very effective. Our framework (construction of an integrated and weighted FLN plus the application of the NW rule) compares favorably with exiting methods (see below). We choose the NW rule due to its simplicity and effectiveness, and leave to future work the exploration of other more complex, diffusion-type rules.

Finally, the combination of the NW rule with weighted FLN is a common practice in the field. For example, Lee *et al* and McGary *et al* have successfully applied the same NW rule to integrated FLNs in worm and yeast to predict perturbation phenotypes of genes [6, 34].

NW rule is a local decision rule, and it uses only immediate network neighbors for new disease gene prediction. Other non-local decision rules have been proposed for disease gene predictions, such as the shortest path rule and the random walk rule [35, 36]. These rules make use of indirect connections between non-adjacent nodes for predictions. These rules are usually applied to networks that are much sparser than our FLN. For example, in Wu *et al.*'s study [35] where the shortest path rule is used, the input network is an unweighted protein-protein physical interaction network (14,433 nodes, 72,431 edges, and about 10 edges per node). In Kohler *et al.*' study [36] where a random walk rule is used, the input network is an unweighted functional linkage network (13,726 nodes, 258,314 edges, and about 38 edges per node). Because the average number of seed genes of the OMIM diseases is about 10, a local rule using only the direct neighbors of seeds can only rank hundreds of neighboring genes in both of these networks, and non-local rules are more desirable in these networks. In contrast, our weighted FLN is much denser (over 21,000 genes, over 22,000,000 edges, and over 2,000 edges per node). For each disease, our NW rule can already rank about 12,000 genes based on their weighted links to the seed genes. Thus our NW rule is most suited for our dense, weighted FLN.

4. ROC-50 analysis as the disease centric evaluation of the FLN based disease gene prioritization.

We have also performed the disease centric evaluations using only the area of the extreme left side of the ROC curve, which represents the top ranking predictions.

We plot the true positive rate (sensitivity) versus the false positive rate (1-specificity) and show the ROC curve with up to the 50th false positives (ROC-50) (Figure S2). For a particular disease, the true positive rate is defined as the fraction of seed genes above a S_i cutoff, and the false positive rate is defined as the fraction of non-seed genes above a S_i cut-off (see Methods). Figure S2 shows that our disease gene predictions are significantly better than random for all seven diseases.

To further use ROC-50 to evaluate all 110 diseases, we calculated the area under the ROC-50 (AUC-50) for each of the 110 diseases, and normalized it to [0, 1]. Figure S3 shows the box plots for FLN based predictions versus random controls (see Methods for random control generations). Again, our FLN based predictions (median AUC-50 of about 0.4) significantly outperforms random controls (median AUC-50 of 0). This trend still holds even when text mining data is excluded from the FLN.

5. Comparison with other disease gene prioritization methods

We compare our FLN-based disease gene prioritization with another network based disease gene prioritization approach, which was recently proposed by Kohler et al [36]. In particular, Kohler et al. constructed a functional linkage network composed of heterogeneous PPI and functional links imported from STRING database [19]. Next they applied a random walk algorithm for disease gene prioritization.

To compare different methods, the same benchmark set should be used for comparison. So we use the same disease seed gene sets provided by Kohler *et al.* for performance comparison. These seed sets represent 110 diseases. Particularly, in leave-one-out cross validation, the performance is compared by the mean fold enrichment, with the artificial chromosomal region containing 100 genes as the background set (see the main

manuscript). A similar performance criteria has also been used by other groups [35, 37, 38]. The mean fold enrichment of our approach is 41.8 (with text mining) and 25.3 (without text mining), slightly lower than Kohler et al.'s method (44 with text mining and 27 without text mining, computed by Kohler et al. [36]), but comparable, especially considering that our FLN has a higher gene coverage and thus allows scanning more genes for prioritization (21,657 genes in our FLN versus less than 14,000 genes in Kohler *et al*'s gene network).

Additionally, with text mining data included, Kohler *et al* utilized 7 recently identified disease genes not included in the seed sets to assess the novel-prediction performance and compared their performance with other approaches. The disease-association landmark references of these 7 genes were after the publication of the STRING database where Kohler et al. retrieved their text mining data [19], therefore relatively minimal literature bias was expected. Since our text mining data are from the same STRING database, with the text mining data included, we also evaluate our method using these 7 disease genes. The result indicates that our method (mean fold enrichment of 30.7) even slightly outperforms Kohler et al's approach (mean fold enrichment of 25.9) (Table S4). The other two methods in comparison, ENDEAVOUR, which also utilizes data integration [30], and PROSPECTOR, which is based on sequence data [29], also have lower mean fold enrichment than ours (mean fold enrichment of 18.4 and 10.9, respectively, calculated by Kohler et al. [36]).

Both our approach and Kohler's could be summarized as a common two-step framework composed of (i) building an FLN using multiple data sources and (ii) applying a decision rule to rank candidate genes based on their direct or indirect connection to the seed genes.

In the first step, Kohler et al. constructed a less optimal FLN than ours as illustrated below. Instead of data integration, they simply took the union of a heterogeneous set of PPI links and functional links from STRING database [19]. It is of note that no linkage weighting was utilized, despite the fact that links vary in confidence levels. For instance, othology mapped PPI from other species has higher error rates than curated PPI derived

from the HPRD database [7]. As for the functional links imported from String database [19], instead of using linkage weights calculated by the String database, a confidence cutoff was applied prior to network construction, where links above the cutoff were included without weighting, and links below the cut-off were simply discarded. Our results suggest that some information could be lost by removing entirely these low confidence links, as demonstrated by Figure S5B and S5C.

6. P-value calculation for the obesity case study

There are a total of 21,657 genes in FLN, among which 24 are obesity seed genes based on OMIM database, and the rest 21,633 are non-seed genes. Among these 21,633 non-seed genes, 334 are included in the obesity candidate genes collected from literature by Hancock *et al.* (referred as “ObesHancock genes”) [39]. If one selects 100 genes at random from the non-seed genes of the FLN, its overlap with the “ObesHancock gene” set follows a binomial distribution, with an average overlap of 334/21,633×100= 1.54.

Among the top 100 predicted candidate obesity genes by the FLN (excluding text mining), 22 genes overlap with the “ObesHancock genes”. The *p*-value for the observed overlap can be estimated based on the above-mentioned binomial distribution, and is less than 10^{-13}.

7. Comparison of FLN based mutual predictability method with Goh et al’s disease gene sharing method for identification of disease-disease associations

We perform a quantitative assessment for comparing our FLN based mutual predictability method with Goh et al’s disease gene sharing method, which identified the associations between two diseases by counting their overlapping disease genes [33]. Since both our method and Goh et al’s method identify the disease-disease association from the aspect of underlining molecular mechanisms, we need a disease-disease association benchmark dataset based on related underlining molecular mechanisms for

quantitative comparison. Unfortunately, there is no such benchmark available since our understanding for molecular mechanisms manifesting individual diseases is still limited and remains to be further explored.

Goh et al divided the OMIM diseases into 20 disease classes based on the clinical phenotypes. Since diseases with similar phenotypes tend to have related molecular mechanisms [4], we use this phenotype-based classification to generate a disease-disease association benchmark. Specifically, disease pairs with both disease members belonging to the same disease class (co-class disease pairs) are considered to be associated. It should be noted that this phenotype based disease classification benchmark is only an approximation: diseases with the same phenotypes can have different molecular mechanisms, and diseases with different phenotypes can share related molecular mechanisms [40, 41]. Therefore, the comparison results based on this approximate phenotypic benchmark should be interpreted in a semi-quantitative way.

Next we compare the two methods at different disease-disease association score cutoffs (using mutual predictability scores for our method and number of overlapping disease genes for Goh et al's method) using the sensitivity versus precision plot (Figure S8), where sensitivity is defined as the fraction of co-class disease pairs above the threshold among all the co-class disease pairs, and precision is defined as the fraction of co-class disease pairs among the disease pairs above the threshold.

The comparison results are shown for two scenarios: (i) identification of co-class disease pairs for those disease pairs that share one or more known disease genes; (ii) identification of co-class disease pairs for those disease pairs that share no known disease genes. For scenario (i), we find that the two methods have comparable performances (Figure S8A). However, for scenario (ii), the disease-gene sharing method fails completely with random performance, as these disease pairs share no known disease genes and all have the same association score of 0 (Figure S8B). On the contrary, our mutual predictability method can still take advantage of the FLN to quantify the association for these disease pairs, and our method clearly outperforms the disease-gene

sharing method (Figure S8B). It should be noted the disease pairs sharing disease genes only cover 15% of all the co-class disease pairs (maximum sensitivity of ~0.15 in Figure S8A), and 85% of co-class disease pairs are missed by disease-gene sharing method. On the contrary, our mutual predictability method can identify additional 20% co-class pairs at the precision cutoff of 0.2, which is more than twice of the precision of the disease-gene sharing method (Figure S8B).

Therefore, the above quantitative comparison confirms that our FLN based mutual predictability method is able to identify additional disease-disease associations sharing no known disease genes, which traditional disease-gene sharing method fails to identify.

Figure S1

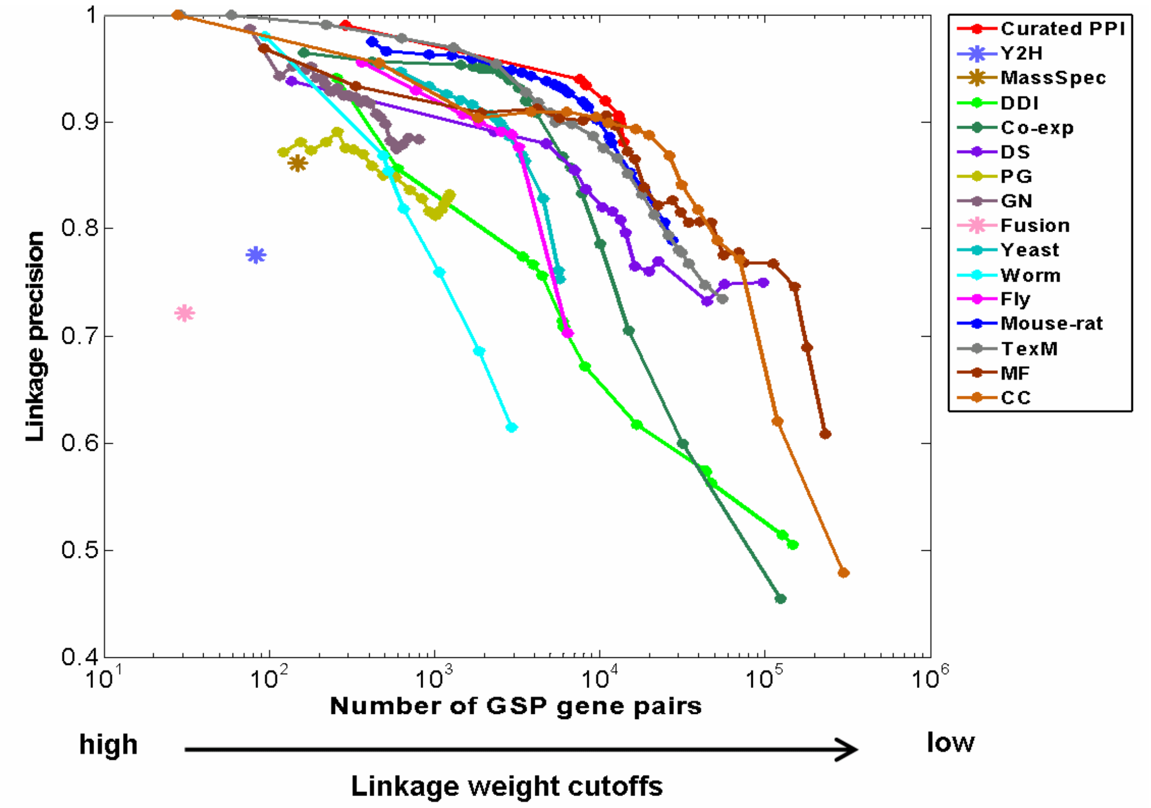

Figure S1: Individual data sources for inferring functional links. The x-axis denotes the number of GSP gene pairs that are linked at different linkage weight cutoffs, this count represents the linkage sensitivity (see Methods of the main manuscript). Note the x-axis is on a log scale. The Y axis denotes corresponding linkage precisions (see Methods of the main manuscript). The individual data sources are described in Table 1 of the main manuscript.

Figure S2

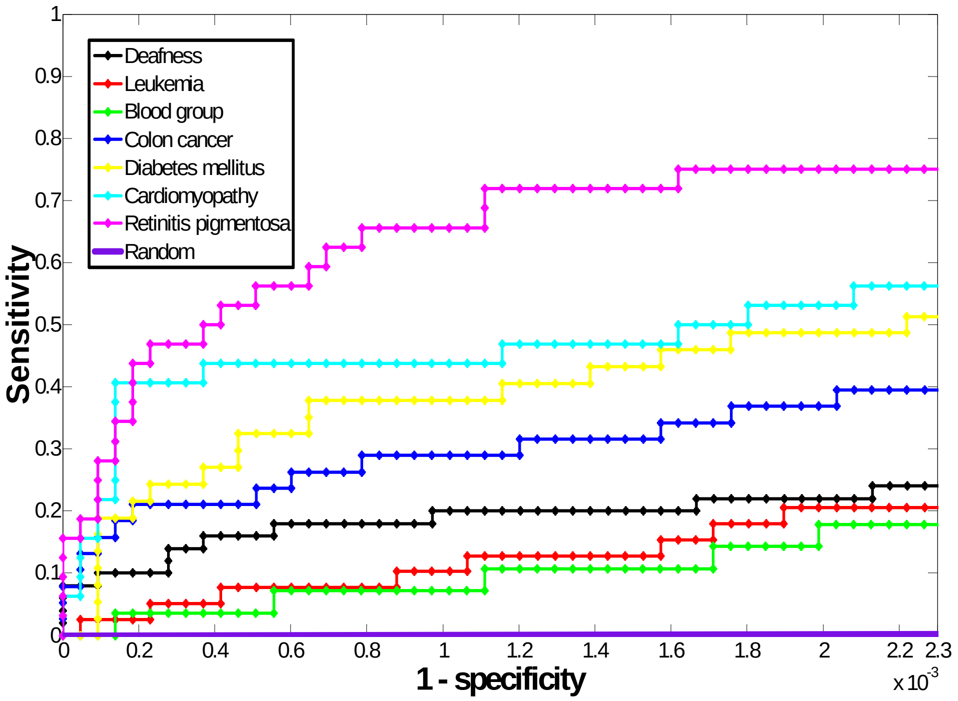

Figure S2: Predictability of 7 example diseases evaluated by ROC-50 curves in the disease-centric assessment. Prediction performance for individual diseases is measured by the true positive rate (sensitivity) versus false positive rate (1-specificity). In particular, for each given disease, each gene in the network is ranked based on the disease association score (S_i, Equation 4). The S_i for each known disease (seed) gene is computed using leave-one-out cross-validation, based on its connectivity to other seeds. Next the performance for each disease is assessed by calculating the sensitivity (TP/(TP +FN)) and 1-specificity (FP/(TN + FP)) at different S_i cut-offs. Here TP is the number of seed genes above the S_i cut-off, FP is the number of non-seed genes above the cut-off, TN is the number of non-seed genes below the cut-off, FN is the number of seed genes below the cut-off. Random prediction performance is indicated by the purple color. Only the partial regions (with up to the 50th false positives) of the ROC curves, the ROC-50s, are shown. Because the false positives range from 0 to 50 for ROC-50 and for each disease there are over 21,000 non seed genes in the FLN, the maximum false positive rate shown is less than 50/21,000 (about 0.0023). The true positive rate ranges between 0 and 1. Therefore, for ROC-50, 1-specificity (X axis) and sensitivity (Y axis) are in different scales.

Because of the scale difference between the X axis and Y axis, unlike the full ROC curve, the expected random control for ROC-50 is not the diagonal any more, but a line linking the data point of (0,0) and the data point of (0.0023, 0.0023), a line very close to X axis.

Figure S3

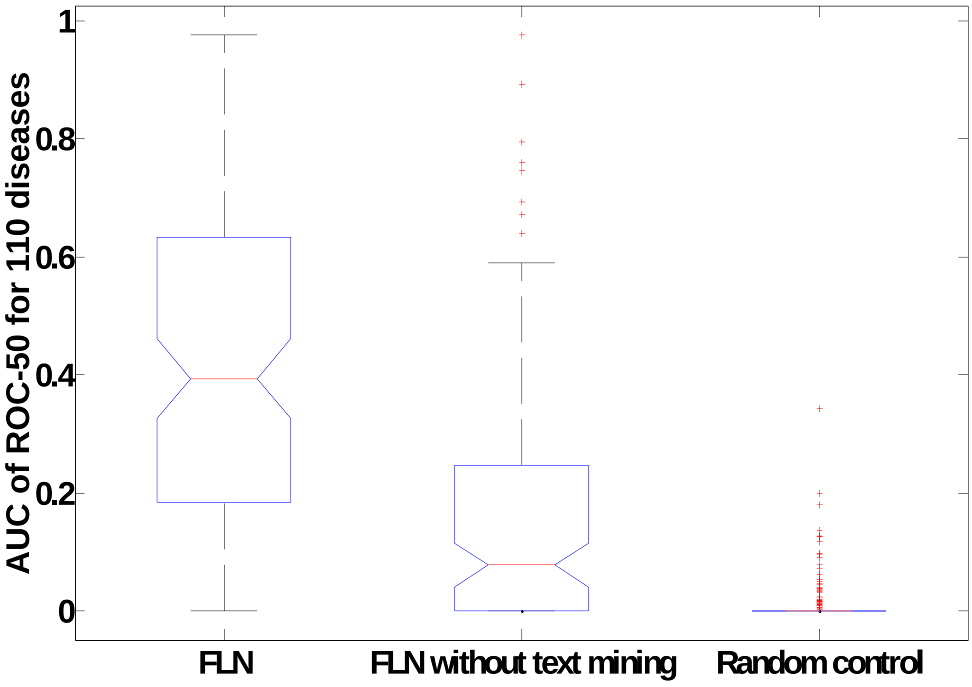

Figure S3: FLN-based disease gene prioritization significantly outperforms random control based on the disease centric evaluation (using the AUC of ROC-50). Performances are compared between FLN (inclusion or exclusion of text mining data) based disease-gene prioritization and the random control. The random control is generated using the FLN to prioritize randomly assembled disease gene sets (See Methods). Box plots of AUC of ROC-50 for 110 diseases, based on the disease-centric assessment (See Methods), are shown. For each box plot, the bottom, middle, and top lines of the box represent the first quartile, the median, and the third quartile, respectively; whiskers represent 1.5 times the inter-quartile range; red plus signs represent outliers.

Figure S4

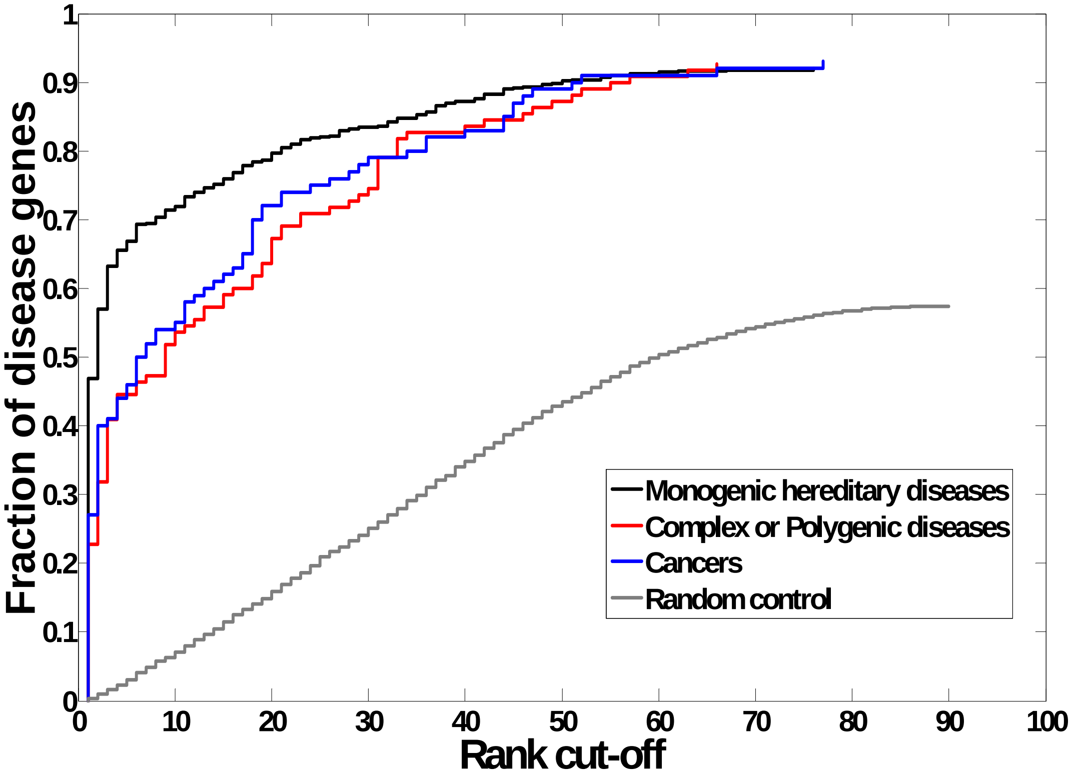

Figure S4: Performance comparison among monogenic diseases, complex diseases, and cancers. The classification of mongenic diseases, complex diseases, and cancers are based on Kohl et al's manual classification [36]. Gene-centric assessment is used for performance comparison (see Method). Gene-centric assessment treats each known gene-disease association as a test case. For each test case, the task is to assess how well the known disease (seed) gene ranks relative to a background gene set according to the disease-association score(S_i) (Equation 4 of the main manuscript). The S_i for each gene in each test case is calculated in leave-one-out setting based on the connectivity to the remaining seed genes. The background gene set used is referred as the artificial chromosomal interval, which is composed of a collection of 100 nearest genes flanking the tested disease gene physically on the chromosome. Finally, after the rank of each tested disease gene within each test case is determined, all the test cases are pooled together and the overall performance is assessed by evaluating the fraction of the tested disease genes ranked above various rank cut-offs. Note: FLN excluding text mining is used for all the comparisons.

Figure S5

S5A

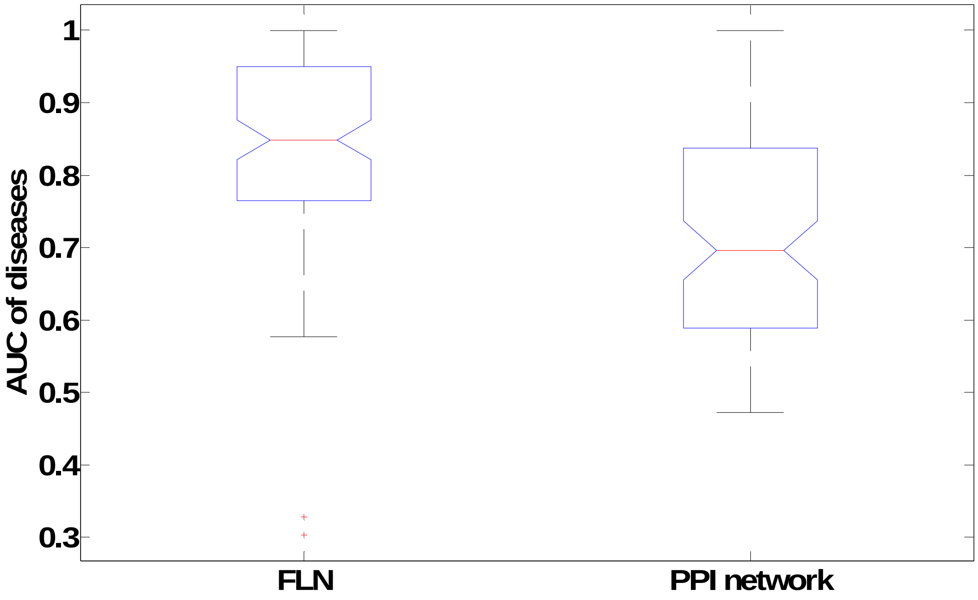

S5B

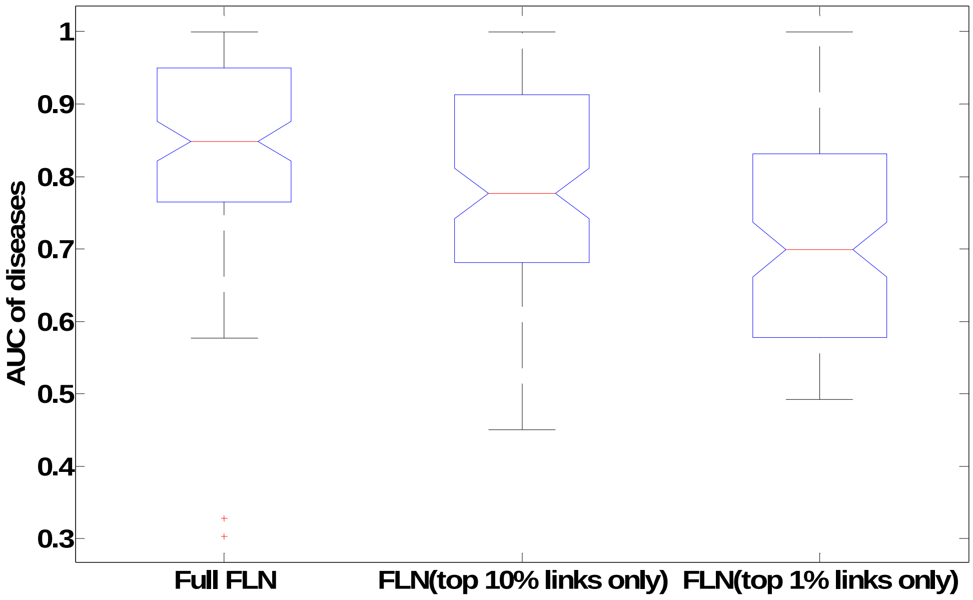

S5C

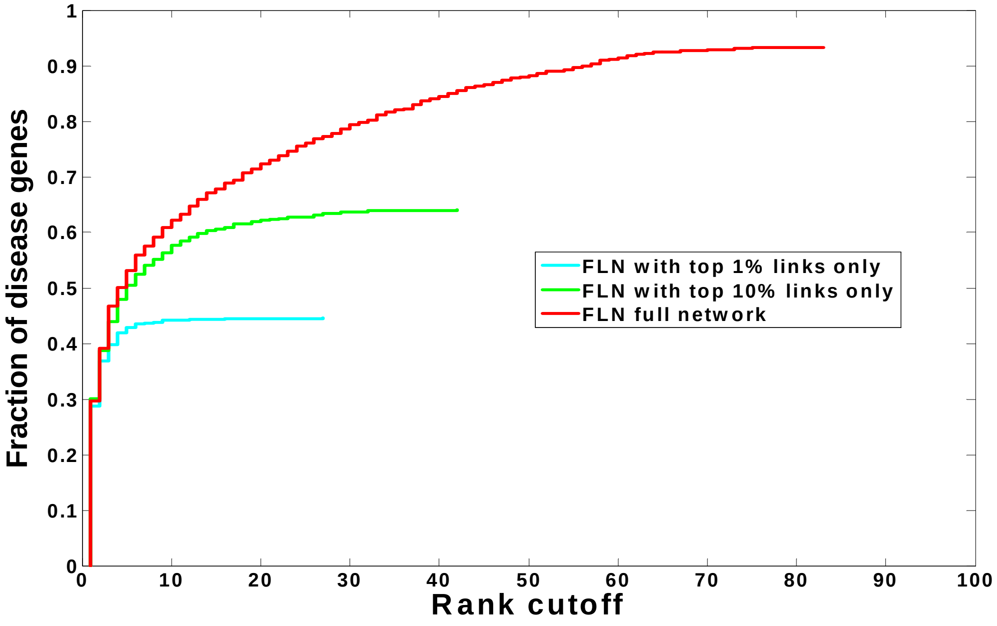

Figure S5: Data integration is important for FLN based disease gene prioritization. **(A)** FLN based disease prioritization shows improvement over the PPI network based on the disease centric assessment (see Methods). Box plots of AUCs of disease-gene-prioritization performances for 110 diseases, are compared between FLN based prioritization and PPI based prioritization. For each box plot, the bottom, middle, and top lines of the box represent the first quantile, the median, and the third quantile, respectively; whiskers represent 1.5 times the interquartile range; red plus signs represent outliers. **(B)** Lower weighted functional links also contribute to prioritization performance since the full FLN outperforms filtered networks. Links in the FLN are ranked in descending order based on linkage weight, FLNs composed of top 1%, 10% and 100% links are compared for prioritization performance. Disease centric assessment is used for comparison (see Methods). **(C)** Same comparison as (B) based on the gene centric assessment (see Method). Gene-centric assessment treats each known gene-disease association as a test case. For each test case, the task is to assess how well the known disease (seed) gene ranks relative to a background gene set according to the disease-association score(S_i) (Equation 4 of the main manuscript). The S_i for each gene in each test case is calculated in leave-one-out setting based on the connectivity to the

remaining seed genes. The background gene set used is referred as the artificial chromosomal interval, which is composed of a collection of 100 nearest genes flanking the tested disease gene physically on the chromosome. Finally, after the rank of each tested disease gene within each test case is determined, all the test cases are pooled together and the overall performance is assessed by evaluating the fraction of the tested disease genes ranked above various rank cut-offs.

Figure S6

S6A

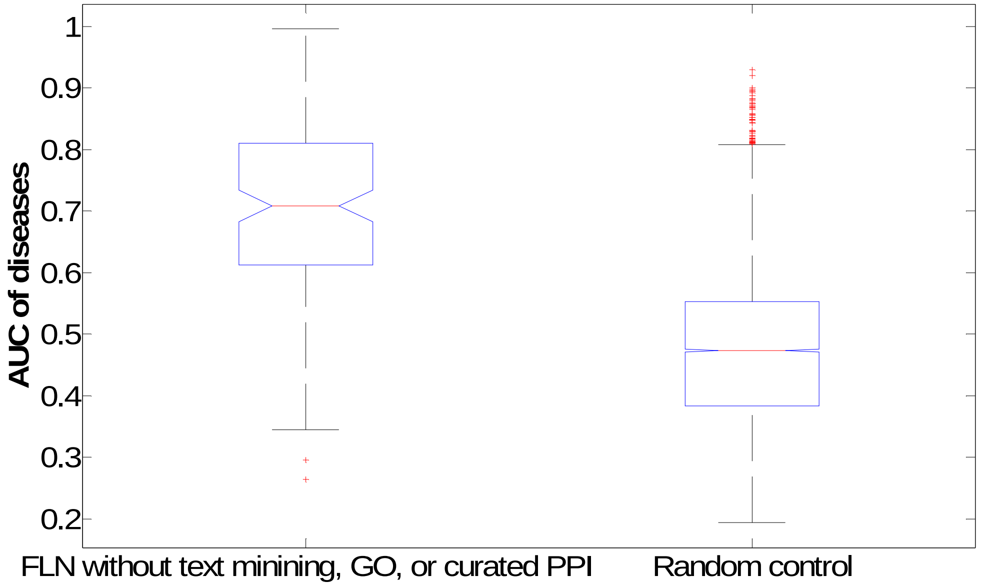

S6B

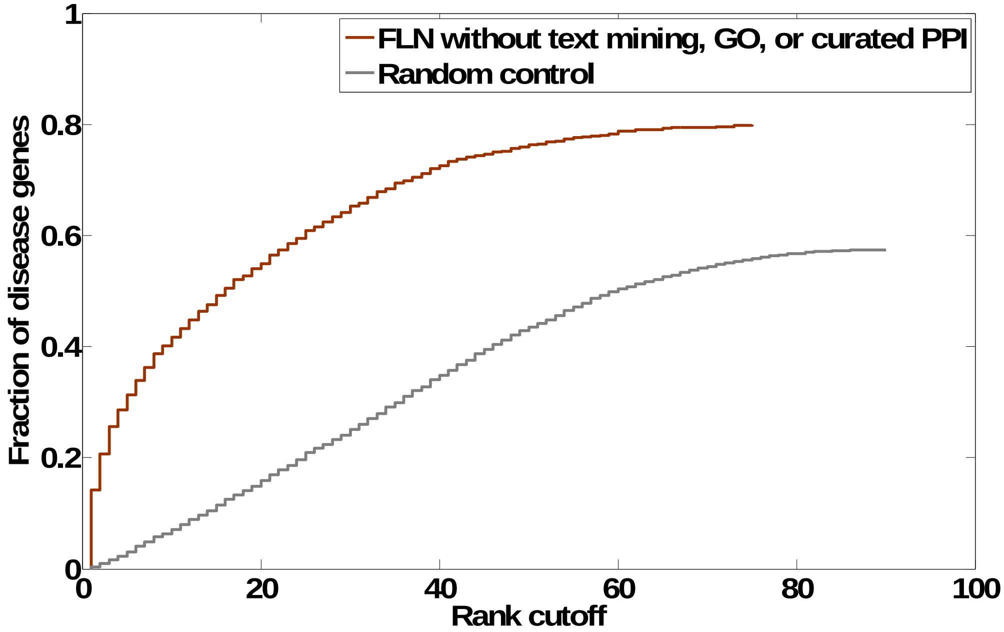

Figure S6: FLN-based disease gene prioritization does not rely on text mining, GO Molecular Function, GO Cellular Component, or curated PPI alone. FLN excluding text mining (human, mouse and rat), GO Molecular Function, GO Cellular Component, and curated PPI (human, mouse, and rat) is evaluated for disease gene prioritization in comparison with random control. **(A)** Disease centric assessment (see Methods). Box plots of AUCs of disease-gene-prioritization performance for 110 diseases, are compared between FLN-based prioritization and random control. For each box plot, the bottom, middle, and top lines of the box represent the first quantile, the median, and the third quantile, respectively; whiskers represent 1.5 times the interquartile range; red plus signs represent outliers. **(B)** Gene centric assessment (See Method). Gene-centric assessment treats each known gene-disease association as a test case. For each test case, the task is to assess how well the known disease (seed) gene ranks relative to a background gene set according to the disease-association score(S_i) (Equation 4 of the main manuscript). The S_i for each gene in each test case is calculated in leave-one-out setting based on the connectivity to the remaining seed genes. The background gene set used is referred as the artificial chromosomal interval, which is composed of a collection of 100 nearest genes flanking the tested disease gene physically on the chromosome. Finally, after the rank of each tested disease gene within each test case is determined, all the test cases are pooled together and the overall performance is assessed by evaluating the fraction of the tested disease genes ranked above various rank cut-offs.

Figure S7

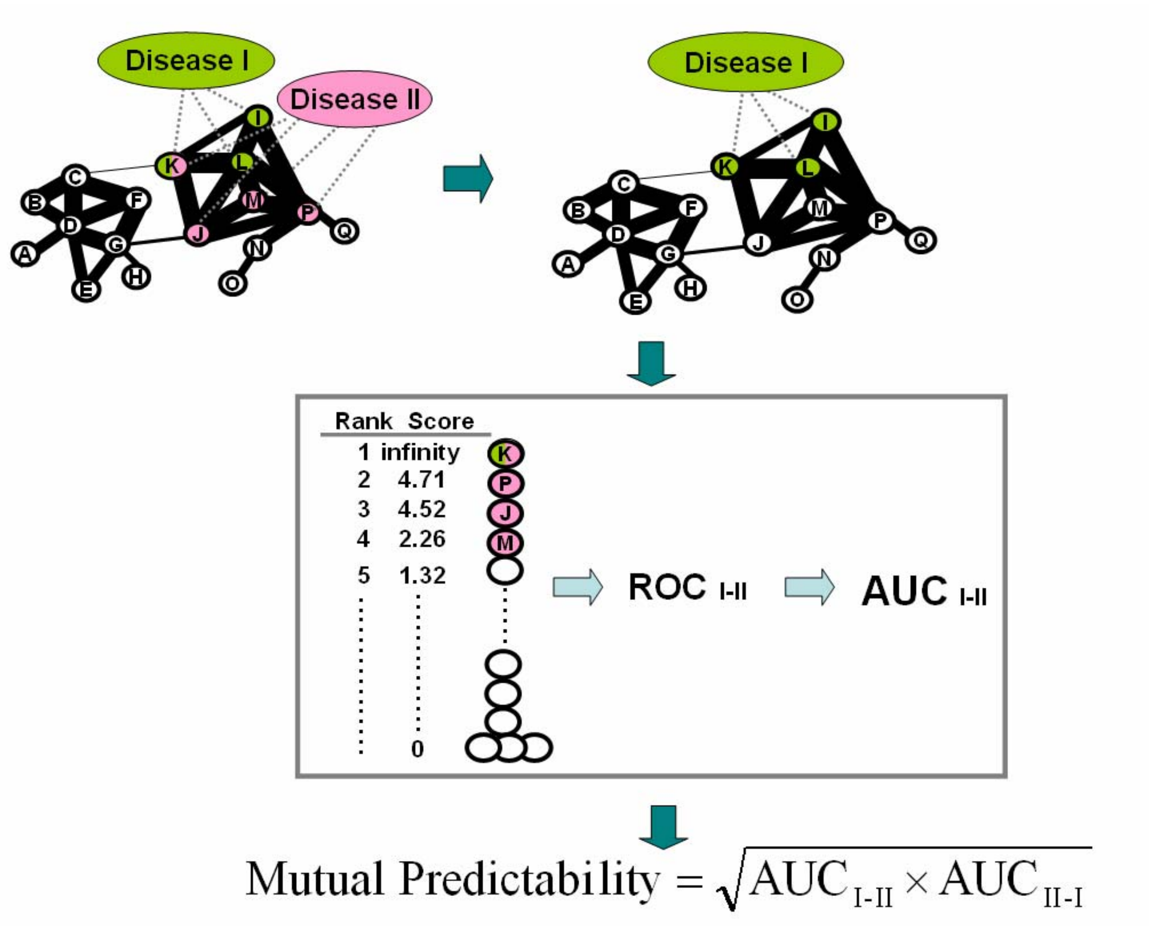

Figure S7: **Calculation of the mutual predictability score between disease I and disease II.** Disease I is associated with 3 known genes, K, I, and L, and disease II is associated with 4 known genes, K, M, P, and J. One of the disease genes, K, is shared. Mutual predictability evaluates how well the known disease genes in I can be used to identify the disease genes in II (predictability $_{I\text{-}II}$) and vice versa (predictability $_{II\text{-}I}$).To quantify predictability $_{I\text{-}II}$, we first use genes associated with disease I as seeds, and rank all other genes based on the S_i score (Equation 4 of the main manuscript). In addition, disease II genes that overlap with the seed genes from disease I, gene K, is given a S_i score of infinity and ranked on the top. Next, we plot a ROC curve of sensitivity *versus* 1-specificity on how well the disease II genes are ranked in the sorted gene list (see Methods of the main manuscript). Finally, we calculate the AUC of the ROC curve

(AUC_{I-II}) as a measure for predictability $_{I-II}$. Similarly, we can calculate AUC_{II-I} as a measure of predictability $_{II-I}$. The mutual predictability between disease I and II is defined as the geometric mean of AUC_{I-II} and AUC_{II-I}.

Figure S8

A

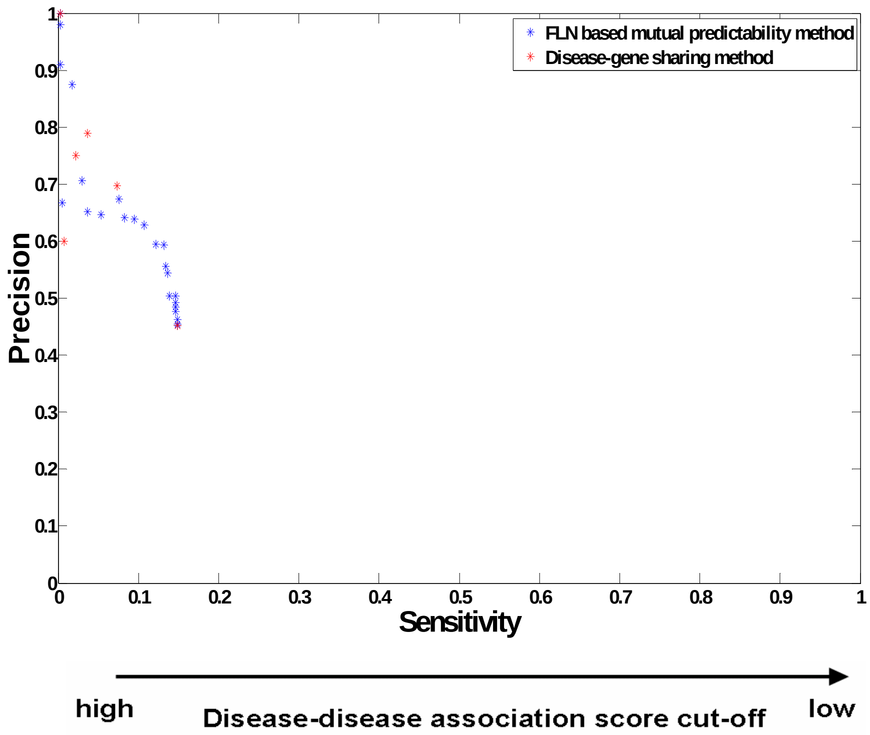

FLN based mutual predictability method
Disease-gene sharing method
Precision
Sensitivity
high
Disease-disease association score cut-off
low

B

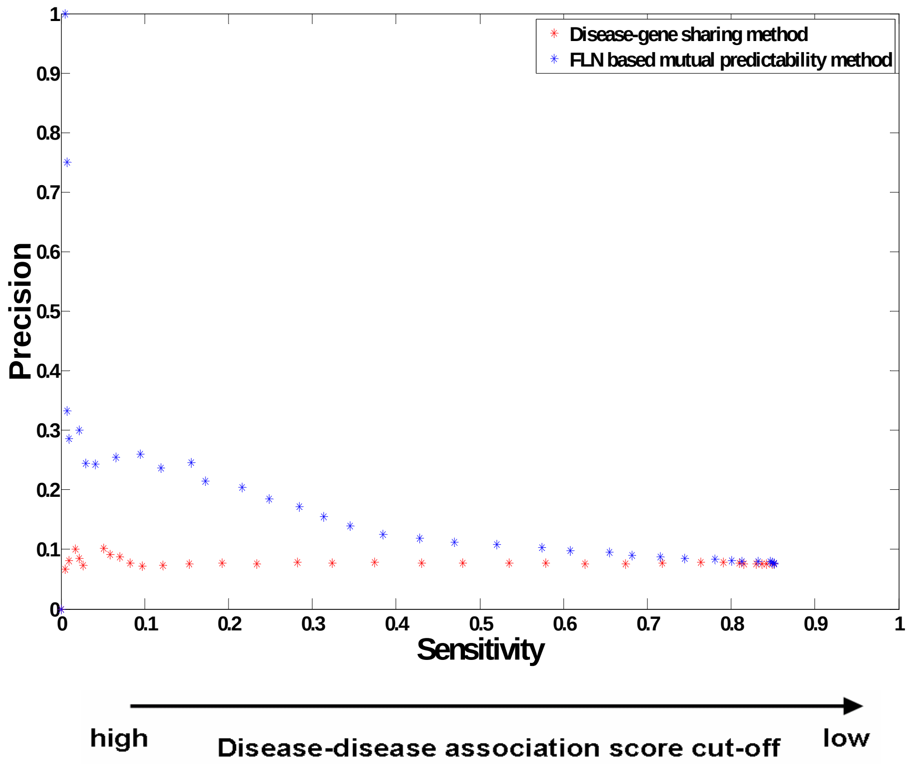

Disease-gene sharing method
FLN based mutual predictability method
Precision
Sensitivity
high
Disease-disease association score cut-off
low

Figure S8: Comparison of mutual predictability method and disease-gene sharing method. ***(A)*** **Comparison for the scenario where the evaluated disease pairs share one or more disease genes.** The comparison is performed at various disease-disease association score cut-offs. The disease-disease association score for mutual predictability method is the mutual predictability score; the disease-disease association score for disease-gene sharing method is the number of overlapping disease genes between a disease pair. The number of overlapping disease genes is a discrete value ranging from 1 to 6 (based on OMIM); the mutual predictability score is a continuous value ranging from 0 to 1 (see Methods in the main manuscript). X axis denotes *sensitivity*: fraction of co-class disease pairs above the threshold among all the co-class disease pairs; Y axis denotes *precision*: fraction of co-class disease pairs among the disease pairs above the threshold. The disease classification is based on Goh *et al* manual classification [4]. Since 15% of co-class disease pairs share disease genes, the maximum sensitivity is about 0.15. ***(B)*** **Comparison for the scenario where the evaluated disease pairs share no known disease genes. Disease-gene sharing method cannot differentiate these disease pairs, as they all have the same association score of 0.** To plot the data points for the disease-gene sharing method, we rank these disease pairs randomly and threshold the randomized rank score to plot the *sensitivity* and *precision*. Since 85% of co-class disease pairs share no disease genes, the maximum sensitivity is about 0.85. The sum of the maximum sensitivity in (A) and the maximum sensitivity in (B) is 1.

Figure S9

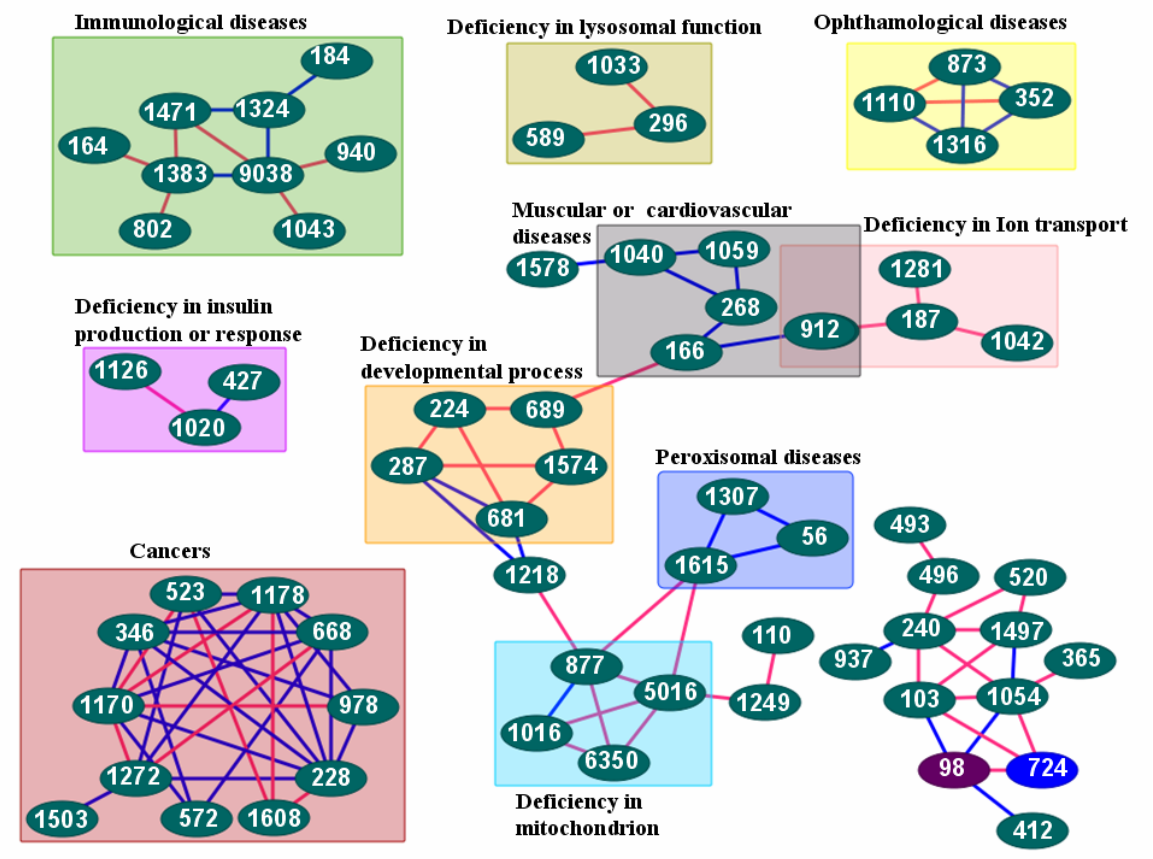

Figure S9: An example network of 66 diseases (nodes) linked by top 100 edges with the highest mutual predictability scores. The disease nodes are labeled with disease IDs (see Additional File 2). Many of these edges cannot be identified using simple disease-gene sharing method, and these are colored red. The rest of the edges are colored blue. These top 100 disease pairs, their mutual predictability scores, and the supporting evidence for association are listed in Additional file 9. This disease-disease association network has a modular topology, and diseases tend to form clusters (rectangles). The disease clusters are labeled based on enriched disease class or underlying molecular mechanisms (see Additional File 10).

Table S1: Number of gene pairs in each PPI source.

	**Curated PPI databases**	**Rual et al Y2H**	**Ewing et al MassSepct**
**Number of gene pairs**	90,352	2,611	2,046

Table S2: Number of overlapping gene pairs between different PPI sources.

	**Curated human PPI databases**	**Rual et al Y2H**	**Ewing et al MassSepct**
**Curated human PPI databases**	\	157	335
**Rual et al Y2H**	157	\	8
**Ewing et al MassSepct**	335	8	\

Table S3: Percentage of overlapping gene pairs between different PPI sources.

	**Curated human PPI databases**	**Rual et al Y2H**	**Ewing et al MassSepct**
**Curated human PPI databases**	\	157/90,352 = 0.17%	335/90,352 = 0.37%
**Rual et al Y2H**	157/2,611= 6.0%	\	8/2,611 = 0.3%
**Ewing et al MassSepct**	335/2,046 = 16.4%	8/2,046=0.39%	\

Table S4: Comparison of four disease gene prioritization methods to predict seven recently identified disease genes.

Diseases	Genes	Rankings			
		**Kohler et al**	**ENDEAVOUR**	**PROSPECTR**	**Our method**
**Nephronophthisis**	*GLIS237*	100	43	3*	100
**ARVD**	*JUP38*	1*	1*	67	1*
**RP**	*TOPORS39*	23*	69	56	23*
**RP**	*NR2E340*	2	2	1	1*
**Noonan Syndrome**	*RAF141*	1*	3	42	1*
**Brachydactyly**	*NOG42*	1*	5	34	1*
**CMT4H**	*FGD443*	13	2*	9	4
**Mean Fold Enrichment**		25.9	18.4	10.9	30.7*

Kohler's approach [36], ENDEAVOUR [30] , and PROSPECTR [29], and our method are compared for predicting 7 recently identified disease causing genes, using artificial chromosomal region containing 100 genes as the background set. The gene ranking and fold enrichments for the other 3 compared methods except our own were computed by Kohler et al (see the original Table 2 in Kohler et al [36]). Best performances are labeled with *.

ARVD: Arrhythmogenic right ventricular dysplasia

RP: retinitis pigmentosa

CMT4H: Charcot-Marie-Tooth type 4H

References

1. Zhou X, Kao MC, Wong WH: **Transitive functional annotation by shortest-path analysis of gene expression data**. *Proc Natl Acad Sci U S A* 2002, **99**(20):12783-12788.
2. Hughes TR, Roth FP: **A race through the maze of genomic evidence**. *Genome Biol* 2008, **9 Suppl 1**:S1.
3. Huang Y, Li H, Hu H, Yan X, Waterman MS, Huang H, Zhou XJ: **Systematic discovery of functional modules and context-specific functional annotation of human genome**. *Bioinformatics* 2007, **23**(13):i222-229.
4. Maglott D, Ostell J, Pruitt KD, Tatusova T: **Entrez Gene: gene-centered information at NCBI**. *Nucleic Acids Res* 2007, **35**(Database issue):D26-31.
5. Harris MA, Clark J, Ireland A, Lomax J, Ashburner M, Foulger R, Eilbeck K, Lewis S, Marshall B, Mungall C *et al*: **The Gene Ontology (GO) database and informatics resource**. *Nucleic Acids Res* 2004, **32**(Database issue):D258-261.
6. Lee I, Lehner B, Crombie C, Wong W, Fraser AG, Marcotte EM: **A single gene network accurately predicts phenotypic effects of gene perturbation in Caenorhabditis elegans**. *Nat Genet* 2008, **40**(2):181-188.
7. Mishra GR, Suresh M, Kumaran K, Kannabiran N, Suresh S, Bala P, Shivakumar K, Anuradha N, Reddy R, Raghavan TM *et al*: **Human protein reference database--2006 update**. *Nucleic Acids Res* 2006, **34**(Database issue):D411-414.
8. Bader GD, Betel D, Hogue CW: **BIND: the Biomolecular Interaction Network Database**. *Nucleic Acids Res* 2003, **31**(1):248-250.
9. Breitkreutz BJ, Stark C, Reguly T, Boucher L, Breitkreutz A, Livstone M, Oughtred R, Lackner DH, Bahler J, Wood V *et al*: **The BioGRID Interaction Database: 2008 update**. *Nucleic Acids Res* 2008, **36**(Database issue):D637-640.
10. Kerrien S, Alam-Faruque Y, Aranda B, Bancarz I, Bridge A, Derow C, Dimmer E, Feuermann M, Friedrichsen A, Huntley R *et al*: **IntAct--open source resource for molecular interaction data**. *Nucleic Acids Res* 2007, **35**(Database issue):D561-565.
11. Mewes HW, Dietmann S, Frishman D, Gregory R, Mannhaupt G, Mayer KF, Munsterkotter M, Ruepp A, Spannagl M, Stumpflen V *et al*: **MIPS: analysis and annotation of genome information in 2007**. *Nucleic Acids Res* 2008, **36**(Database issue):D196-201.
12. Salwinski L, Miller CS, Smith AJ, Pettit FK, Bowie JU, Eisenberg D: **The Database of Interacting Proteins: 2004 update**. *Nucleic Acids Res* 2004, **32**(Database issue):D449-451.
13. Chatr-Aryamontri A, Zanzoni A, Ceol A, Cesareni G: **Searching the Protein Interaction Space Through the MINT Database**. *Methods Mol Biol* 2008, **484**:305-317.
14. Rual JF, Venkatesan K, Hao T, Hirozane-Kishikawa T, Dricot A, Li N, Berriz GF, Gibbons FD, Dreze M, Ayivi-Guedehoussou N *et al*: **Towards a proteome-scale map of the human protein-protein interaction network**. *Nature* 2005, **437**(7062):1173-1178.

15. Ewing RM, Chu P, Elisma F, Li H, Taylor P, Climie S, McBroom-Cerajewski L, Robinson MD, O'Connor L, Li M *et al*: **Large-scale mapping of human protein-protein interactions by mass spectrometry**. *Mol Syst Biol* 2007, **3**:89.
16. Rhodes DR, Tomlins SA, Varambally S, Mahavisno V, Barrette T, Kalyana-Sundaram S, Ghosh D, Pandey A, Chinnaiyan AM: **Probabilistic model of the human protein-protein interaction network**. *Nat Biotechnol* 2005, **23**(8):951-959.
17. Scott MS, Barton GJ: **Probabilistic prediction and ranking of human protein-protein interactions**. *BMC Bioinformatics* 2007, **8**:239.
18. Mulder NJ, Apweiler R, Attwood TK, Bairoch A, Bateman A, Binns D, Bradley P, Bork P, Bucher P, Cerutti L *et al*: **InterPro, progress and status in 2005**. *Nucleic Acids Res* 2005, **33**(Database issue):D201-205.
19. von Mering C, Jensen LJ, Kuhn M, Chaffron S, Doerks T, Kruger B, Snel B, Bork P: **STRING 7--recent developments in the integration and prediction of protein interactions**. *Nucleic Acids Res* 2007, **35**(Database issue):D358-362.
20. Lee HK, Hsu AK, Sajdak J, Qin J, Pavlidis P: **Coexpression analysis of human genes across many microarray data sets**. *Genome Res* 2004, **14**(6):1085-1094.
21. Griffith OL, Pleasance ED, Fulton DL, Oveisi M, Ester M, Siddiqui AS, Jones SJ: **Assessment and integration of publicly available SAGE, cDNA microarray, and oligonucleotide microarray expression data for global coexpression analyses**. *Genomics* 2005, **86**(4):476-488.
22. Jensen LJ, Lagarde J, von Mering C, Bork P: **ArrayProspector: a web resource of functional associations inferred from microarray expression data**. *Nucleic Acids Res* 2004, **32**(Web Server issue):W445-448.
23. von Mering C, Jensen LJ, Snel B, Hooper SD, Krupp M, Foglierini M, Jouffre N, Huynen MA, Bork P: **STRING: known and predicted protein-protein associations, integrated and transferred across organisms**. *Nucleic Acids Res* 2005, **33**(Database issue):D433-437.
24. von Mering C, Huynen M, Jaeggi D, Schmidt S, Bork P, Snel B: **STRING: a database of predicted functional associations between proteins**. *Nucleic Acids Res* 2003, **31**(1):258-261.
25. Lee I, Li Z, Marcotte EM: **An Improved, Bias-Reduced Probabilistic Functional Gene Network of Baker's Yeast, Saccharomyces cerevisiae**. *PLoS ONE* 2007, **2**(10):e988.
26. Berglund AC, Sjolund E, Ostlund G, Sonnhammer EL: **InParanoid 6: eukaryotic ortholog clusters with inparalogs**. *Nucleic Acids Res* 2008, **36**(Database issue):D263-266.
27. **The Gene Ontology project in 2008**. *Nucleic Acids Res* 2007.
28. Franke L, Bakel H, Fokkens L, de Jong ED, Egmont-Petersen M, Wijmenga C: **Reconstruction of a functional human gene network, with an application for prioritizing positional candidate genes**. *Am J Hum Genet* 2006, **78**(6):1011-1025.
29. Adie EA, Adams RR, Evans KL, Porteous DJ, Pickard BS: **SUSPECTS: enabling fast and effective prioritization of positional candidates**. *Bioinformatics* 2006, **22**(6):773-774.

30. Aerts S, Lambrechts D, Maity S, Van Loo P, Coessens B, De Smet F, Tranchevent LC, De Moor B, Marynen P, Hassan B *et al*: **Gene prioritization through genomic data fusion**. *Nat Biotechnol* 2006, **24**(5):537-544.
31. Zhong W, Sternberg PW: **Genome-wide prediction of C. elegans genetic interactions**. *Science* 2006, **311**(5766):1481-1484.
32. Hamosh A, Scott AF, Amberger JS, Bocchini CA, McKusick VA: **Online Mendelian Inheritance in Man (OMIM), a knowledgebase of human genes and genetic disorders**. *Nucleic Acids Res* 2005, **33**(Database issue):D514-517.
33. Goh KI, Cusick ME, Valle D, Childs B, Vidal M, Barabasi AL: **The human disease network**. *Proc Natl Acad Sci U S A* 2007, **104**(21):8685-8690.
34. McGary KL, Lee I, Marcotte EM: **Broad network-based predictability of Saccharomyces cerevisiae gene loss-of-function phenotypes**. *Genome Biol* 2007, **8**(12):R258.
35. Wu X, Jiang R, Zhang MQ, Li S: **Network-based global inference of human disease genes**. *Mol Syst Biol* 2008, **4**:189.
36. Kohler S, Bauer S, Horn D, Robinson PN: **Walking the interactome for prioritization of candidate disease genes**. *Am J Hum Genet* 2008, **82**(4):949-958.
37. Lage K, Karlberg EO, Storling ZM, Olason PI, Pedersen AG, Rigina O, Hinsby AM, Tumer Z, Pociot F, Tommerup N *et al*: **A human phenome-interactome network of protein complexes implicated in genetic disorders**. *Nat Biotechnol* 2007, **25**(3):309-316.
38. Ala U, Piro RM, Grassi E, Damasco C, Silengo L, Oti M, Provero P, Di Cunto F: **Prediction of human disease genes by human-mouse conserved coexpression analysis**. *PLoS Comput Biol* 2008, **4**(3):e1000043.
39. Hancock AM, Witonsky DB, Gordon AS, Eshel G, Pritchard JK, Coop G, Di Rienzo A: **Adaptations to climate in candidate genes for common metabolic disorders**. *PLoS Genet* 2008, **4**(2):e32.
40. Oti M, Brunner HG: **The modular nature of genetic diseases**. *Clin Genet* 2007, **71**(1):1-11.
41. Oti M, Huynen MA, Brunner HG: **Phenome connections**. *Trends Genet* 2008, **24**(3):103-106.
