# Supplementary material for: Genome-wide prioritization of disease genes and identification of disease-disease associations from an integrated human functional linkage network
Source: Genome Biol. 2009 Sep 3;10(9):R91. doi: 10.1186/gb-2009-10-9-r91 (PMC2768980; doi:10.1186/gb-2009-10-9-r91)
Supplement: Additional data file 6 — Recently identified disease genes and landmark references dated after January 2007. [file gb-2009-10-9-r91-S6.pdf]

Recent identified disease genes with landmark references after January 2007.

Gene symbol	Disease ID	Disease name in OMIM	References
SORL1	98	{Alzheimer disease, pathogenesis, association with}, 104300 (3)	[1]
FAM83H	99	Amelogenesis imperfecta, type 3, 130900 (3)	[2]
IRAK3	153	{Asthma susceptibility 5}, 611064 (3)	[3]
CASP8	228	{Breast cancer, protection against}, 114480 (3)	[4]
AKT1	228	Breast cancer, somatic, 114480 (3)	[5]
HMMR	228	{Breast cancer, susceptibility to}, 114480 (3)	[6]
PALB2	228	{Breast cancer, susceptibility to}, 114480 (3)	[7]
BFSP1	277	Cataract, cortical, juvenile-onset, 611391 (3)	[8]
CHMP4B	277	Cataract, posterior polar, 3, 605387 (3)	[9]
FGD4	301	Charcot-Marie-Tooth disease, type 4H, 609311 (3)	[10]
FIG4	301	Charcot-Marie-Tooth disease, type 4J, 611228 (3)	[11]
PRPS1	301	Charcot-Marie-Tooth disease, X-linked recessive, 5, 311070 (3)	[12]
AKT1	346	Colorectal cancer, somatic, 114500 (3)	[5]
SMAD7	346	{Colorectal cancer, susceptiblity to}, 114500 (3)	[13]
COG8	354	Congenital disorder of glycosylation, type IIh, 611182 (3)	[14]
TMEM15	354	Congenital disorder of glycosylation, type Im, 610768 (3)	[15]
UBIAD1	362	Corneal dystrophy, crystalline, of Schnyder, 121800 (3)	[16]
LRP6	365	Coronary artery disease, autosomal dominant 2, 610947 (3)	[17]
CCDC50	406	Deafness, autosomal dominant 44, 607453 (3)	[18]
RDX	406	Deafness, autosomal recessive, 24, 611022 (3)	[19]
ESRRB	406	Deafness, autosomal recessive 35, 608565 (3)	[20]
FGF3	406	Deafness, congenital with inner ear agenesis, microtia, and microdontia, 610706 (3)	[21]
WFS1	427	{Diabetes mellitus, noninsulin-dependent, association with}, 125853 (3)	[22]
CDKAL1	427	{Diabetes mellitus, noninsulin-dependent, susceptibility to}, 125853 (3)	[23]

IGF2BP2	427	{Diabetes mellitus, noninsulin-dependent, susceptibility to}, 125853 (3)	[23]
SLC30A8	427	{Diabetes mellitus, noninsulin-dependent, susceptibility to}, 135853 (3)	[24]
TAF1	462	Dystonia-Parkinsonism, X-linked, 314250 (3)	[25]
KCTD7	495	Epilepsy, progressive myoclonic 3, 611726 (3)	[26]
LCA5	873	Leber congenital amaurosis, type V, 604537 (3)	[27]
SCN4B	912	Long QT syndrome-10, 611819 (3)	[28]
AKAP9	912	Long QT syndrome-11, 611820 (3)	[29]
C3	937	{Macular degeneration, age-related, 9}, 611378 (3)	[30]
CX3CR1	937	{Macular degeneration, age-related, susceptibility to}, 602075 (3)	[31]
NCR3	940	{Malaria, mild, susceptibility to}, 609148 (3)	[32]
TIRAP	940	{Malaria, protection against}, 611162 (3)	[33]
FCGR2B	940	{Malaria, resistance to}, 611162 (3)	[34]
MBD5	990	Mental retardation, autosomal dominant 1, 156200 (3)	[35]
DOCK8	990	Mental retardation, autosomal dominant 2 (3)	[36]
GRIK2	990	Mental retardation, autosomal recessive, 6, 611092 (3)	[37]
DIP2B	990	Mental retardation, FRA12A type, 136630 (3)	[38]
CUL4B	990	Mental retardation-hypotonic facies syndrome, X-linked, 2, 300639 (3)	[39]
BRWD3	990	Mental retardation, X-linked 93, 300659 (3)	[40]
GRIA3	990	Mental retardation, X-linked 94, 300699 (3)	[41]
UPF3B	990	Mental retardation, X-linked, syndromic 14, 300676 (3)	[42]
BMP4	1012	Microphthalmia, syndromic 6, 607932 (3)	[43]
STRA6	1012	Microphthalmia, syndromic 9, 601186 (3)	[44]
C6orf66	1016	Mitochondrial complex I deficiency, 252010 (3)	[45]
LRP8	1054	{Myocardial infarction, susceptibility to}, 608446 (3)	[46]
FTO	1126	{Obesity, associated with}, 601665 (3)	[47]
TNFSF11	1161	Osteopetrosis, autosomal recessive 2, 259710 (3)	[48]

PLEKHM1	1161	Osteopetrosis, autosomal recessive 6, 611497 (3)	[49]
AKT1	1170	Ovarian cancer, somatic, 604370 (3)	[5]
HERC2	1227	[Skin/hair/eye pigmentation 1, blond/brown hair], 227220 (3)	[50]
TYR	1227	[Skin/hair/eye pigmentation 3, freckling], 601800 (3)	[50]
SLC24A2	1227	[Skin/hair/eye pigmentation 6, blond/brown hair], 210750 (3)	[50]
KITLG	1227	[Skin/hair/eye pigmentation 7, blond/brown hair], 611664 (3)	[50]
HNF1B	1272	{Prostate cancer, susceptibility to}, 176807 (3)	[51]
TOPORS	1316	Retinitis pigmentosa-31, 609923 (3)	[52]
STAT4	1324	{Rheumatoid arthritis, association with}, 180300 (3)	[53]
TREX1	1471	{Systemic lupus erythematosus, susceptibility to}, 152700 (3)	[54]
CR2	1471	{Systemic lupus erythematosus, susceptibility to, 9}, 610927 (3)	[55]
STAT4	1471	{Systemic lupus erythematous, association with}, 152700 (3)	[53]
BANK1	1471	{Systemic lupus erythmatosus, association with}, 152700 (3)	[56]
LARGE	1578	Walker-Warburg syndrome, 236670 (3)	[57]

Reference:

1. Rogaeva E, Meng Y, Lee JH, Gu Y, Kawarai T, Zou F, Katayama T, Baldwin CT, Cheng R, Hasegawa H *et al*: **The neuronal sortilin-related receptor SORL1 is genetically associated with Alzheimer disease**. *Nat Genet* 2007, **39**(2):168-177.
2. Kim JW, Lee SK, Lee ZH, Park JC, Lee KE, Lee MH, Park JT, Seo BM, Hu JC, Simmer JP: **FAM83H mutations in families with autosomal-dominant hypocalcified amelogenesis imperfecta**. *Am J Hum Genet* 2008, **82**(2):489-494.
3. Balaci L, Spada MC, Olla N, Sole G, Loddo L, Anedda F, Naitza S, Zuncheddu MA, Maschio A, Altea D *et al*: **IRAK-M is involved in the pathogenesis of early-onset persistent asthma**. *Am J Hum Genet* 2007, **80**(6):1103-1114.
4. Cox A, Dunning AM, Garcia-Closas M, Balasubramanian S, Reed MW, Pooley KA, Scollen S, Baynes C, Ponder BA, Chanock S *et al*: **A common coding variant in CASP8 is associated with breast cancer risk**. *Nat Genet* 2007, **39**(3):352-358.
5. Carpten JD, Faber AL, Horn C, Donoho GP, Briggs SL, Robbins CM, Hostetter G, Boguslawski S, Moses TY, Savage S *et al*: **A transforming mutation in the**

pleckstrin homology domain of AKT1 in cancer. *Nature* 2007, **448**(7152):439-444.
6. Pujana MA, Han JD, Starita LM, Stevens KN, Tewari M, Ahn JS, Rennert G, Moreno V, Kirchhoff T, Gold B *et al*: **Network modeling links breast cancer susceptibility and centrosome dysfunction**. *Nat Genet* 2007, **39**(11):1338-1349.
7. Rahman N, Seal S, Thompson D, Kelly P, Renwick A, Elliott A, Reid S, Spanova K, Barfoot R, Chagtai T *et al*: **PALB2, which encodes a BRCA2-interacting protein, is a breast cancer susceptibility gene**. *Nat Genet* 2007, **39**(2):165-167.
8. Ramachandran RD, Perumalsamy V, Hejtmancik JF: **Autosomal recessive juvenile onset cataract associated with mutation in BFSP1**. *Hum Genet* 2007, **121**(3-4):475-482.
9. Shiels A, Bennett TM, Knopf HL, Yamada K, Yoshiura K, Niikawa N, Shim S, Hanson PI: **CHMP4B, a novel gene for autosomal dominant cataracts linked to chromosome 20q**. *Am J Hum Genet* 2007, **81**(3):596-606.
10. Delague V, Jacquier A, Hamadouche T, Poitelon Y, Baudot C, Boccaccio I, Chouery E, Chaouch M, Kassouri N, Jabbour R *et al*: **Mutations in FGD4 encoding the Rho GDP/GTP exchange factor FRABIN cause autosomal recessive Charcot-Marie-Tooth type 4H**. *Am J Hum Genet* 2007, **81**(1):1-16.
11. Chow CY, Zhang Y, Dowling JJ, Jin N, Adamska M, Shiga K, Szigeti K, Shy ME, Li J, Zhang X *et al*: **Mutation of FIG4 causes neurodegeneration in the pale tremor mouse and patients with CMT4J**. *Nature* 2007, **448**(7149):68-72.
12. Kim HJ, Sohn KM, Shy ME, Krajewski KM, Hwang M, Park JH, Jang SY, Won HH, Choi BO, Hong SH *et al*: **Mutations in PRPS1, which encodes the phosphoribosyl pyrophosphate synthetase enzyme critical for nucleotide biosynthesis, cause hereditary peripheral neuropathy with hearing loss and optic neuropathy (cmtx5)**. *Am J Hum Genet* 2007, **81**(3):552-558.
13. Broderick P, Carvajal-Carmona L, Pittman AM, Webb E, Howarth K, Rowan A, Lubbe S, Spain S, Sullivan K, Fielding S *et al*: **A genome-wide association study shows that common alleles of SMAD7 influence colorectal cancer risk**. *Nat Genet* 2007, **39**(11):1315-1317.
14. Kranz C, Ng BG, Sun L, Sharma V, Eklund EA, Miura Y, Ungar D, Lupashin V, Winkel RD, Cipollo JF *et al*: **COG8 deficiency causes new congenital disorder of glycosylation type IIh**. *Hum Mol Genet* 2007, **16**(7):731-741.
15. Kranz C, Jungeblut C, Denecke J, Erlekotte A, Sohlbach C, Debus V, Kehl HG, Harms E, Reith A, Reichel S *et al*: **A defect in dolichol phosphate biosynthesis causes a new inherited disorder with death in early infancy**. *Am J Hum Genet* 2007, **80**(3):433-440.
16. Weiss JS, Kruth HS, Kuivaniemi H, Tromp G, White PS, Winters RS, Lisch W, Henn W, Denninger E, Krause M *et al*: **Mutations in the UBIAD1 gene on chromosome short arm 1, region 36, cause Schnyder crystalline corneal dystrophy**. *Invest Ophthalmol Vis Sci* 2007, **48**(11):5007-5012.
17. Mani A, Radhakrishnan J, Wang H, Mani A, Mani MA, Nelson-Williams C, Carew KS, Mane S, Najmabadi H, Wu D *et al*: **LRP6 mutation in a family with early coronary disease and metabolic risk factors**. *Science* 2007, **315**(5816):1278-1282.

18. Modamio-Hoybjor S, Mencia A, Goodyear R, del Castillo I, Richardson G, Moreno F, Moreno-Pelayo MA: **A mutation in CCDC50, a gene encoding an effector of epidermal growth factor-mediated cell signaling, causes progressive hearing loss**. *Am J Hum Genet* 2007, **80**(6):1076-1089.
19. Khan SY, Ahmed ZM, Shabbir MI, Kitajiri S, Kalsoom S, Tasneem S, Shayiq S, Ramesh A, Srisailpathy S, Khan SN *et al*: **Mutations of the RDX gene cause nonsyndromic hearing loss at the DFNB24 locus**. *Hum Mutat* 2007, **28**(5):417-423.
20. Collin RW, Kalay E, Tariq M, Peters T, van der Zwaag B, Venselaar H, Oostrik J, Lee K, Ahmed ZM, Caylan R *et al*: **Mutations of ESRRB encoding estrogen-related receptor beta cause autosomal-recessive nonsyndromic hearing impairment DFNB35**. *Am J Hum Genet* 2008, **82**(1):125-138.
21. Tekin M, Hismi BO, Fitoz S, Ozdag H, Cengiz FB, Sirmaci A, Aslan I, Inceoglu B, Yuksel-Konuk EB, Yilmaz ST *et al*: **Homozygous mutations in fibroblast growth factor 3 are associated with a new form of syndromic deafness characterized by inner ear agenesis, microtia, and microdontia**. *Am J Hum Genet* 2007, **80**(2):338-344.
22. Sandhu MS, Weedon MN, Fawcett KA, Wasson J, Debenham SL, Daly A, Lango H, Frayling TM, Neumann RJ, Sherva R *et al*: **Common variants in WFS1 confer risk of type 2 diabetes**. *Nat Genet* 2007, **39**(8):951-953.
23. Scott LJ, Mohlke KL, Bonnycastle LL, Willer CJ, Li Y, Duren WL, Erdos MR, Stringham HM, Chines PS, Jackson AU *et al*: **A genome-wide association study of type 2 diabetes in Finns detects multiple susceptibility variants**. *Science* 2007, **316**(5829):1341-1345.
24. Sladek R, Rocheleau G, Rung J, Dina C, Shen L, Serre D, Boutin P, Vincent D, Belisle A, Hadjadj S *et al*: **A genome-wide association study identifies novel risk loci for type 2 diabetes**. *Nature* 2007, **445**(7130):881-885.
25. Makino S, Kaji R, Ando S, Tomizawa M, Yasuno K, Goto S, Matsumoto S, Tabuena MD, Maranon E, Dantes M *et al*: **Reduced neuron-specific expression of the TAF1 gene is associated with X-linked dystonia-parkinsonism**. *Am J Hum Genet* 2007, **80**(3):393-406.
26. Van Bogaert P, Azizieh R, Desir J, Aeby A, De Meirleir L, Laes JF, Christiaens F, Abramowicz MJ: **Mutation of a potassium channel-related gene in progressive myoclonic epilepsy**. *Ann Neurol* 2007, **61**(6):579-586.
27. den Hollander AI, Koenekoop RK, Mohamed MD, Arts HH, Boldt K, Towns KV, Sedmak T, Beer M, Nagel-Wolfrum K, McKibbin M *et al*: **Mutations in LCA5, encoding the ciliary protein lebercilin, cause Leber congenital amaurosis**. *Nat Genet* 2007, **39**(7):889-895.
28. Medeiros-Domingo A, Kaku T, Tester DJ, Iturralde-Torres P, Itty A, Ye B, Valdivia C, Ueda K, Canizales-Quinteros S, Tusie-Luna MT *et al*: **SCN4B-encoded sodium channel beta4 subunit in congenital long-QT syndrome**. *Circulation* 2007, **116**(2):134-142.
29. Chen L, Marquardt ML, Tester DJ, Sampson KJ, Ackerman MJ, Kass RS: **Mutation of an A-kinase-anchoring protein causes long-QT syndrome**. *Proc Natl Acad Sci U S A* 2007, **104**(52):20990-20995.

30. Yates JR, Sepp T, Matharu BK, Khan JC, Thurlby DA, Shahid H, Clayton DG, Hayward C, Morgan J, Wright AF *et al*: **Complement C3 variant and the risk of age-related macular degeneration**. *N Engl J Med* 2007, **357**(6):553-561.
31. Combadiere C, Feumi C, Raoul W, Keller N, Rodero M, Pezard A, Lavalette S, Houssier M, Jonet L, Picard E *et al*: **CX3CR1-dependent subretinal microglia cell accumulation is associated with cardinal features of age-related macular degeneration**. *J Clin Invest* 2007, **117**(10):2920-2928.
32. Delahaye NF, Barbier M, Fumoux F, Rihet P: **Association analyses of NCR3 polymorphisms with P. falciparum mild malaria**. *Microbes Infect* 2007, **9**(2):160-166.
33. Khor CC, Chapman SJ, Vannberg FO, Dunne A, Murphy C, Ling EY, Frodsham AJ, Walley AJ, Kyrieleis O, Khan A *et al*: **A Mal functional variant is associated with protection against invasive pneumococcal disease, bacteremia, malaria and tuberculosis**. *Nat Genet* 2007, **39**(4):523-528.
34. Clatworthy MR, Willcocks L, Urban B, Langhorne J, Williams TN, Peshu N, Watkins NA, Floto RA, Smith KG: **Systemic lupus erythematosus-associated defects in the inhibitory receptor FcgammaRIIb reduce susceptibility to malaria**. *Proc Natl Acad Sci U S A* 2007, **104**(17):7169-7174.
35. Wagenstaller J, Spranger S, Lorenz-Depiereux B, Kazmierczak B, Nathrath M, Wahl D, Heye B, Glaser D, Liebscher V, Meitinger T *et al*: **Copy-number variations measured by single-nucleotide-polymorphism oligonucleotide arrays in patients with mental retardation**. *Am J Hum Genet* 2007, **81**(4):768-779.
36. Griggs BL, Ladd S, Saul RA, DuPont BR, Srivastava AK: **Dedicator of cytokinesis 8 is disrupted in two patients with mental retardation and developmental disabilities**. *Genomics* 2008, **91**(2):195-202.
37. Motazacker MM, Rost BR, Hucho T, Garshasbi M, Kahrizi K, Ullmann R, Abedini SS, Nieh SE, Amini SH, Goswami C *et al*: **A defect in the ionotropic glutamate receptor 6 gene (GRIK2) is associated with autosomal recessive mental retardation**. *Am J Hum Genet* 2007, **81**(4):792-798.
38. Winnepenninckx B, Debacker K, Ramsay J, Smeets D, Smits A, FitzPatrick DR, Kooy RF: **CGG-repeat expansion in the DIP2B gene is associated with the fragile site FRA12A on chromosome 12q13.1**. *Am J Hum Genet* 2007, **80**(2):221-231.
39. Zou Y, Liu Q, Chen B, Zhang X, Guo C, Zhou H, Li J, Gao G, Guo Y, Yan C *et al*: **Mutation in CUL4B, which encodes a member of cullin-RING ubiquitin ligase complex, causes X-linked mental retardation**. *Am J Hum Genet* 2007, **80**(3):561-566.
40. Field M, Tarpey PS, Smith R, Edkins S, O'Meara S, Stevens C, Tofts C, Teague J, Butler A, Dicks E *et al*: **Mutations in the BRWD3 gene cause X-linked mental retardation associated with macrocephaly**. *Am J Hum Genet* 2007, **81**(2):367-374.
41. Wu Y, Arai AC, Rumbaugh G, Srivastava AK, Turner G, Hayashi T, Suzuki E, Jiang Y, Zhang L, Rodriguez J *et al*: **Mutations in ionotropic AMPA receptor 3 alter channel properties and are associated with moderate cognitive impairment in humans**. *Proc Natl Acad Sci U S A* 2007, **104**(46):18163-18168.

42. Tarpey PS, Raymond FL, Nguyen LS, Rodriguez J, Hackett A, Vandeleur L, Smith R, Shoubridge C, Edkins S, Stevens C *et al*: **Mutations in UPF3B, a member of the nonsense-mediated mRNA decay complex, cause syndromic and nonsyndromic mental retardation**. *Nat Genet* 2007, **39**(9):1127-1133.
43. Bakrania P, Efthymiou M, Klein JC, Salt A, Bunyan DJ, Wyatt A, Ponting CP, Martin A, Williams S, Lindley V *et al*: **Mutations in BMP4 cause eye, brain, and digit developmental anomalies: overlap between the BMP4 and hedgehog signaling pathways**. *Am J Hum Genet* 2008, **82**(2):304-319.
44. Pasutto F, Sticht H, Hammersen G, Gillessen-Kaesbach G, Fitzpatrick DR, Nurnberg G, Brasch F, Schirmer-Zimmermann H, Tolmie JL, Chitayat D *et al*: **Mutations in STRA6 cause a broad spectrum of malformations including anophthalmia, congenital heart defects, diaphragmatic hernia, alveolar capillary dysplasia, lung hypoplasia, and mental retardation**. *Am J Hum Genet* 2007, **80**(3):550-560.
45. Saada A, Edvardson S, Rapoport M, Shaag A, Amry K, Miller C, Lorberboum-Galski H, Elpeleg O: **C6ORF66 is an assembly factor of mitochondrial complex I**. *Am J Hum Genet* 2008, **82**(1):32-38.
46. Shen GQ, Li L, Girelli D, Seidelmann SB, Rao S, Fan C, Park JE, Xi Q, Li J, Hu Y *et al*: **An LRP8 variant is associated with familial and premature coronary artery disease and myocardial infarction**. *Am J Hum Genet* 2007, **81**(4):780-791.
47. Frayling TM, Timpson NJ, Weedon MN, Zeggini E, Freathy RM, Lindgren CM, Perry JR, Elliott KS, Lango H, Rayner NW *et al*: **A common variant in the FTO gene is associated with body mass index and predisposes to childhood and adult obesity**. *Science* 2007, **316**(5826):889-894.
48. Sobacchi C, Frattini A, Guerrini MM, Abinun M, Pangrazio A, Susani L, Bredius R, Mancini G, Cant A, Bishop N *et al*: **Osteoclast-poor human osteopetrosis due to mutations in the gene encoding RANKL**. *Nat Genet* 2007, **39**(8):960-962.
49. Van Wesenbeeck L, Odgren PR, Coxon FP, Frattini A, Moens P, Perdu B, MacKay CA, Van Hul E, Timmermans JP, Vanhoenacker F *et al*: **Involvement of PLEKHM1 in osteoclastic vesicular transport and osteopetrosis in incisors absent rats and humans**. *J Clin Invest* 2007, **117**(4):919-930.
50. Sulem P, Gudbjartsson DF, Stacey SN, Helgason A, Rafnar T, Magnusson KP, Manolescu A, Karason A, Palsson A, Thorleifsson G *et al*: **Genetic determinants of hair, eye and skin pigmentation in Europeans**. *Nat Genet* 2007, **39**(12):1443-1452.
51. Gudmundsson J, Sulem P, Steinthorsdottir V, Bergthorsson JT, Thorleifsson G, Manolescu A, Rafnar T, Gudbjartsson D, Agnarsson BA, Baker A *et al*: **Two variants on chromosome 17 confer prostate cancer risk, and the one in TCF2 protects against type 2 diabetes**. *Nat Genet* 2007, **39**(8):977-983.
52. Chakarova CF, Papaioannou MG, Khanna H, Lopez I, Waseem N, Shah A, Theis T, Friedman J, Maubaret C, Bujakowska K *et al*: **Mutations in TOPORS cause autosomal dominant retinitis pigmentosa with perivascular retinal pigment epithelium atrophy**. *Am J Hum Genet* 2007, **81**(5):1098-1103.

53. Remmers EF, Plenge RM, Lee AT, Graham RR, Hom G, Behrens TW, de Bakker PI, Le JM, Lee HS, Batliwalla F *et al*: **STAT4 and the risk of rheumatoid arthritis and systemic lupus erythematosus**. *N Engl J Med* 2007, **357**(10):977-986.
54. Lee-Kirsch MA, Gong M, Chowdhury D, Senenko L, Engel K, Lee YA, de Silva U, Bailey SL, Witte T, Vyse TJ *et al*: **Mutations in the gene encoding the 3'-5' DNA exonuclease TREX1 are associated with systemic lupus erythematosus**. *Nat Genet* 2007, **39**(9):1065-1067.
55. Wu H, Boackle SA, Hanvivadhanakul P, Ulgiati D, Grossman JM, Lee Y, Shen N, Abraham LJ, Mercer TR, Park E *et al*: **Association of a common complement receptor 2 haplotype with increased risk of systemic lupus erythematosus**. *Proc Natl Acad Sci U S A* 2007, **104**(10):3961-3966.
56. Kozyrev SV, Abelson AK, Wojcik J, Zaghlool A, Linga Reddy MV, Sanchez E, Gunnarsson I, Svenungsson E, Sturfelt G, Jonsen A *et al*: **Functional variants in the B-cell gene BANK1 are associated with systemic lupus erythematosus**. *Nat Genet* 2008, **40**(2):211-216.
57. van Reeuwijk J, Grewal PK, Salih MA, Beltran-Valero de Bernabe D, McLaughlan JM, Michielse CB, Herrmann R, Hewitt JE, Steinbrecher A, Seidahmed MZ *et al*: **Intragenic deletion in the LARGE gene causes Walker-Warburg syndrome**. *Hum Genet* 2007, **121**(6):685-690.
