# Supplementary material for: Genome-wide prioritization of disease genes and identification of disease-disease associations from an integrated human functional linkage network
Source: Genome Biol. 2009 Sep 3;10(9):R91. doi: 10.1186/gb-2009-10-9-r91 (PMC2768980; doi:10.1186/gb-2009-10-9-r91)
Supplement: Additional data file 7 — The 22 predicted obesity genes among the top 100 predicted gene list that overlap with the obesity genes collected from literature by Hanock et al. [58]. [file gb-2009-10-9-r91-S7.pdf]

List of the 22 predicted obesity genes among the top 100 predicted gene list that overlap with the obesity genes collected from literature by Hanock *et al* [17].

Among the top 100 predicted obesity genes based on the functional linkage network (text mining data is excluded), 22 genes overlap with the obesity genes collected by Hancock *et al.* from literature [17]. These genes and the evidence supporting their association with obesity are listed below. The supporting evidences are based on table S3 of Hancock *et al.*'s paper [17].

Gene Symbol	Entrez Gene ID	Rank	Supporting evidence for association with obesity
EP3	5733	2	[2]
INSR	3643	3	[1] [5]
HTR2A	3356	11	[3] [8]
ALPHA1BAR	147	15	[1]
PYY	5697	18	[6]
PYY4	4852	20	[1] [3] [8]
RDC8	135	21	[2]
RXRB	6257	25	[2]
LOC654136	1131	31	[1][8]
NR3A1	2099	33	[2] [3] [12]
DRD4	1815	44	[3] [8]
MGC104991	3570	53	[3] [8]
CCKRA	886	55	[3] [8] [12]
HTR2C	3358	58	[1] [3] [8]
PPARGC1A	10891	64	[3] [16]
HRH1	3269	66	[1]
RHR	153	70	[1] [2] [3]
NPY1R	4886	75	[1] [6]
SLC1	2847	83	[1]
PPY	5539	84	[1]
RXRA	6256	89	[2]
HCRTR2	3062	94	[8]

REFERENCES:

1. Perusse, L., et al., *The human obesity gene map: the 2004 update.* Obes Res, 2005. 13(3): p. 381-490.

2. Ailhaud, G. and H. Hauner, *Development of White Adipose Tissue*, in *Handbook of Obesity: etiology and pathophysiology*, G.A. Bray and C. Bouchard, Editors. 2004, Marcel Dekker, Inc.: New York. p. 481-514.
3. Bouchard, C., et al., *Obesity Gene Map Database*. 2006.
4. Shklyaev, S., et al., *Sustained peripheral expression of transgene adiponectin offsets the development of diet-induced obesity in rats.* Proc Natl Acad Sci U S A, 2003. 100(24): p. 14217-22.
5. Fajas, L. and J. Auwerx, *Peroxisome proliferator-activated receptor gamma and the transcriptional control of adipogenesis and metabolism*, in *Handbook of Obesity: etiology and pathophysiology*, G.A. Bray and C. Bouchard, Editors. 2004, Marcel Dekker, Inc.: New York. p. 559-587.
6. Langin, D. and M. Lafontan, *Lipolysis and lipid mobilization in human adipose tissue*, in *Handbook of Obesity: etiology and pathophysiology*, G.A. Bray and C. Bouchard, Editors. 2004, Marcel Dekker, Inc.: New York. p. 515-532.
7. Kurokawa, N., et al., *Association of BMI with the beta3-adrenergic receptor gene polymorphism in Japanese: meta-analysis.* Obes Res, 2001. 9(12): p. 741-5.
8. Leibowitz, S.F. and B.G. Hoebel, *Behavioral Neuroscience and Obesity*, in *Handbook of Obesity: etiology and pathophysiology*, G.A. Bray and C. Bouchard, Editors. 2004, Marcel Dekker, Inc.: New York. p. 301-371.
9. Collin, G.B., et al., *Alms1-disrupted mice recapitulate human Alstrom syndrome.* Hum Mol Genet, 2005. 14(16): p. 2323-33.
10. Hara-Chikuma, M., et al., *Progressive adipocyte hypertrophy in aquaporin-7-deficient mice: adipocyte glycerol permeability as a novel regulator of fat accumulation.* J Biol Chem, 2005. 280(16): p. 15493-6.
11. Hibuse, T., et al., *Aquaporin 7 deficiency is associated with development of obesity through activation of adipose glycerol kinase.* Proc Natl Acad Sci U S A, 2005. 102(31): p. 10993-8.
12. Allison, D.B., et al., *Genetic Influences on Obesity*, in *Obesity: Mechanisms and Clinical Management*, R. Eckel, Editor. 2003, Elsevier: New York. p. 31-74.
13. He, L., et al., *A biochemical function for attractin in agouti-induced pigmentation and obesity.* Nat Genet, 2001. 27(1): p. 40-7.
14. Turek, F.W., et al., *Obesity and metabolic syndrome in circadian Clock mutant mice.* Science, 2005. 308(5724): p. 1043-5.
15. Gloaguen, I., et al., *Ciliary neurotrophic factor corrects obesity and diabetes associated with leptin deficiency and resistance.* Proc Natl Acad Sci U S A, 1997. 94(12): p. 6456-61.
16. Lowell, B.B. and B.M. Spiegelman, *Towards a molecular understanding of adaptive thermogenesis.* Nature, 2000. 404(6778): p. 652-60.
17. Hancock, A., et al, *Adaptations to Climate in Candidate Genes for Common Metabolic Disorders*. Plos Genetics, 2008 4(2): p. 1-13.
