# Supplementary material for: Genome-wide prioritization of disease genes and identification of disease-disease associations from an integrated human functional linkage network
Source: Genome Biol. 2009 Sep 3;10(9):R91. doi: 10.1186/gb-2009-10-9-r91 (PMC2768980; doi:10.1186/gb-2009-10-9-r91)
Supplement: Additional data file 9 — The top 100 disease pairs with the highest mutual predictability scores and the supporting evidence for the association. [file gb-2009-10-9-r91-S9.pdf]

Top 100 disease pairs with the highest mutual predictability scores and the supporting evidence for the association. The disease pairs are listed in descending order based on the mutual predictability scores. The red rows are disease pairs without sharing disease genes.

Disease ID		Disease name		Mutual predictability	Supporting evidence for association
A	B	A	B		
1615	1307	Zellweger syndrome	Refsum disease	0.9998	Both are peroxisome biogenesis disorders [1]
1615	56	Zellweger syndrome	Adrenoleukodystrophy	0.9997	Both are peroxisome biogenesis disorders [1]
56	1307	Adrenoleukodystrophy	Refsum disease	0.9989	Both are in neurological class based on Goh *et al.*'s disease classification [2]. Both are peroxisome biogenesis disorders [1]
287	681	Central hypoventilation syndrome	Hirschsprung disease	0.9974	Both are caused by deficiency in development [3].
**877**	**6350**	**Leigh syndrome**	**Combined oxidative phosphorylation deficiency**	**0.9726**	**Both are involved in deficiency occurring in mitochondria (OMIM record: 256000, 609060, 610498, 610678, 611719, 610505)**
**877**	**5016**	**Leigh syndrome**	**Mitochondrial DNA depletion syndrome**	**0.9640**	**Both are involved in deficiency occurring in mitochondria (OMIM record: 256000, 251880, 609560)**
**1281**	**187**	**Pseudohypoaldosteronism**	**Bartter syndrome**	**0.9613**	**Both are involved in ion-transport deficiency [4-6].**
877	1016	Leigh syndrome	Mitochondrial complex I/II/III deficiency	0.9526	Both are involved in deficiency occurring in mitochondria (OMIM record: 256000, 252010, 124000, 252011)
**689**	**1574**	**Holoprosencephaly**	**Waardenburg syndrome**	**0.9511**	**Both are caused by deficiency in development [3] (OMIM record: 236100).**
**681**	**1574**	**Hirschsprung disease**	**Waardenburg syndrome**	**0.9468**	**Both are caused by deficiency in development [3]**
**5016**	**6350**	**Mitochondrial DNA depletion syndrome**	**Combined oxidative phosphorylation deficiency**	**0.9410**	**Both are involved in deficiency occurring in mitochondria (OMIM record: 609060, 610498, 610678, 611719, 610505, 251880, 609560).**
1316	352	Retinitis pigmentosa	Cone or cone-rod dystrophy	0.9405	Both are in ophthamological class based on Goh *et al.*'s disease classification [2].

**1178**	**1170**	**Pancreatic cancer or carcinoma**	**Ovarian cancer or carinoma**	**0.9402**	**Both are in cancer class based on Goh *et al*.'s disease classification [2]**
**352**	**1110**	**Cone or cone-rod dystrophy**	**Night blindness**	**0.9400**	**Both are in ophthamological class based on Goh *et al*.'s disease classification [2].**
**287**	**1574**	**Central hypoventilation syndrome**	**Waardenburg syndrome**	**0.9397**	**Both are caused by deficiency in development [3].**
**1016**	**6350**	**Mitochondrial complex I II/III deficiency**	**Combined oxidative phosphorylation deficiency**	**0.9269**	**Both are involved in deficiency occurring in mitochondria (OMIM record: 609060, 610498, 610678, 611719, 610505, 252010, 124000, 252011).**
**1497**	**520**	**Thrombophilia**	**Factor H V X XI XII XIIIA XIIIB deficiency**	**0.9268**	**Both are in hematological class based on Goh *et al*.'s disease classification [2]**
228	523	Breast cancer	Fanconi anemia	0.9266	Fanconi anemia has strong predisposition to cancer [2].
668	1170	Hepatocellular carcinoma	Ovarian cancer or carinoma	0.9247	Both are in cancer class based on Goh *et al*.'s disease classification [2]
873	352	Leber congenital amaurosis	Cone or cone-rod dystrophy	0.9241	Both are in ophthamological class based on Goh *et al*.'s disease classification [2].
**523**	**1608**	**Fanconi anemia**	**Xeroderma pigmentosum**	**0.9240**	**Xeroderma pigmentosum leads to skin cancer and Fanconi anemia has strong predisposition to cancer [2, 7].**
**1016**	**5016**	**Mitochondrial complex I /II/III deficiency**	**Mitochondrial DNA depletion syndrome**	**0.9232**	**Both are involved in deficiency occurring in mitochondria (OMIM record: 252010, 124000, 252011, 251880, 609560).**
228	1178	Breast cancer	Pancreatic cancer or carcinoma	0.9231	Both are in cancer class based on Goh *et al.*'s disease classification [2].
**724**	**103**	**Hypercholesterolemia**	**Amyloidosis**	**0.9228**	**Primary amyloidosis was reported to be associated with hypercholesterolemia [8].**
**346**	**1170**	**Colon cancer**	**Ovarian cancer or carinoma**	**0.9210**	**Both are in cancer class based on Goh *et al*.'s disease classification [2].**

427	1020	Diabetes mellitus	Maturity onset diabetes of the young	0.9205	Both are involved in diminished production of insulin or diminished response to insulin [9, 10].
**240**	**1497**	**Complement system deficiency**	**Thrombophilia**	**0.9193**	**Complement activation is indicated to mediate the induction of thrombosis [11-13].**
268	1040	Cardiomyopathy	Muscular dystrophy	0.9164	Cardiomyopathy is a frequent occurrence in muscular dystrophy [14].
98	103	Alzheimer disease	Amyloidosis	0.9161	Both are in neurological class based on Goh *et al*.'s disease classification [2].
**240**	**520**	**Complement system deficiency**	**Factor V X XI XII XIIIA XIIIB deficiency**	**0.9141**	**"Factor V X XI XII XIIIA XIIIB deficiency" involves in deficiency in coagulation factors [15]. Evidence shows that there is crosstalk between the complement system and coagulation System [15, 16].**
268	1059	Cardiomyopathy	Myopathy	0.91266	Both are involved in muscle contraction deficiency (OMIM record: 115200, 500000, 192600, 601154, 600884, 600858, 161800, 605355, 608810, 160150, 601419, 606768, 255110, 160500).
346	668	Colon cancer	Hepatocellular carcinoma	0.9124	Both are in cancer class based on Goh *et al*.'s disease classification [2].
1383	9038	Severe combined immunodeficiency	Multiple sclerosis	0.9122	Multiple sclerosis is an autoimmune disease that affects the central nervous system [17].
**689**	**224**	**Holoprosencephaly**	**Brachydactyly**	**0.9108**	**Both are involved in deficiency in development [18] (OMIM record: 236100).**
98	1054	Alzheimer disease	Myocardial infarction	0.9094	Vascular disorders have been indicated as risk factors for Alzheimer disease [19].
523	1178	Fanconi anemia	Pancreatic cancer or carcinoma	0.9094	Fanconi anemia has strong predisposition to cancer [2].
**296**	**1033**	**Ceroid-lipofuscinosis**	**Mucopolysaccharidosis**	**0.9083**	**Both are involved in abnormalities of lysosomal function [20].**

**1497**	**103**	**Thrombophilia**	**Amyloidosis**	**0.9076**	**Thrombotic and hemorrhagic complications frequently have been observed in patients with amyloidosis [21].**
228	1170	Breast cancer	Ovarian cancer or carinoma	0.9075	Both are in cancer class based on Goh *et al.*'s disease classification [2]
**912**	**187**	**Long QT syndrome**	**Bartter syndrome**	**0.9055**	**Both are involved in ion-transport deficiency [6, 22].**
572	668	Gastric cancer	Hepatocellular carcinoma	0.9012	Both are in cancer class based on Goh *et al.*'s disease classification [2].
1316	873	Retinitis pigmentosa	Leber congenital amaurosis	0.9011	Both are in ophthamological class based on Goh et al.'s disease classification [2]
1272	1178	Prostate cancer	Pancreatic cancer or carcinoma	0.8976	Both are in cancer class based on Goh *et al.*'s disease classification [2].
**240**	**496**	**Complement system deficiency**	**Epiphyseal dysplasia**	**0.8958**	**Not available**
912	166	Long QT syndrome	Atrial fibrillation or Atrial septal defect	0.8932	Both are in cardiovascular class based on Goh *et al.*'s disease classification [2]
1324	9038	Rheumatoid arthritis	Multiple sclerosis	0.8932	Both are disorders with autoimmune features [17] (OMIM record: 180300).
**1054**	**724**	**Myocardial infarction**	**Hypercholesterolemia**	**0.8919**	**Hypercholesterolemic mice could develop myocardial infarction [23].**
**1170**	**978**	**Ovarian cancer or carinoma**	**Melanoma**	**0.8894**	**Both are in cancer class based on Goh *et al.*'s disease classification [2]**
228	1272	Breast cancer	Prostate cancer	0.8882	Both are in cancer class based on Goh *et al.*'s disease classification [2]
1471	1324	Systemic lupus erythematosus	Rheumatoid arthritis	0.8819	Rheumatoid arthritis has autoimmune features (OMIM record: 180300)and systemic lupus erythematosus is classified as immunological diseases by Goh *et al*. [2].
287	1218	Central hypoventilation syndrome	Pheochromocytoma	0.8811	Both are involved in malfunction of neural crest cells [24, 25].

**1178**	**1608**	**Pancreatic cancer or carcinoma**	**Xeroderma pigmentosum**	**0.8809**	**Xeroderma pigmentosum leads to skin cancer [7].**
**493**	**496**	**Epidermolysis bullosa**	**Epiphyseal dysplasia**	**0.8804**	**Not available**
**1249**	**5016**	**Porphyria**	**Mitochondrial DNA depletion syndrome**	**0.8798**	**Porphyria is caused by the dysfunction of the heme biosynthetic pathway, some enzyme members of this pathway are located in mitochondria. Mitochondrial DNA depletion syndrome are also involved in dysfunction of enzymes in mitochondria [26] (OMIM record: 609560, 251880).**
**1383**	**802**	**Severe combined immunodeficiency**	**Immunodeficiency due to defect in CD3 or with hyper-IgM**	**0.8763**	**Both are in Immunological class based on Goh *et al*.'s disease classification [2].**
681	1218	Hirschsprung disease	Pheochromocytoma	0.8755	Both are involved in malfunction of neural crest cells [24, 25].
**1471**	**9038**	**Systemic lupus erythematosus**	**Multiple sclerosis**	**0.8741**	**Multiple sclerosis is an autoimmune disease that affects the central nervous system [17]. Systemic lupus erythematosus is classified as a Immunological disease by Goh *et al*. [2].**
**240**	**1054**	**Complement system deficiency**	**Myocardial infarction**	**0.8732**	**There is a pathogenetic role for complement activation in the development of myocardial damage after infarction [27].**
**940**	**9038**	**Malaria**	**Multiple sclerosis**	**0.8730**	**Multiple sclerosis is an autoimmune disease that affects the central nervous system [17]. Malaria is defined as a immunological disease by Goh *et al*. [2].**
1040	1578	Muscular dystrophy	Walker-Warburg syndrome	0.8723	Congenital muscular dystrophy is a feature of the Walker-Warburg syndrome [28].
**240**	**103**	**Complement system deficiency**	**Amyloidosis**	**0.8721**	**There was evidence of complement activation in acquired and hereditary amyloid neuropathy [29].**

1178	978	Pancreatic cancer or carcinoma	Melanoma	0.8715	Both are in cancer class based on Goh *et al.*'s disease classification [2].
572	1170	Gastric cancer	Ovarian cancer or carinoma	0.8697	Both are in cancer class based on Goh *et al.*'s disease classification [2].
**1615**	**5016**	**Zellweger syndrome**	**Mitochondrial DNA depletion syndrome**	**0.8688**	**Zellweger syndrome could be coupled with mitochondrial myopathy [30, 31]; Mitochondrial DNA depletion syndrome involves in deficiency in mitochondria (OMIM record: 251880, 609560).**
1324	184	Rheumatoid arthritis	Bare lymphocyte syndrome	0.8683	Rheumatoid arthritis has autoimmune features (OMIM record: 180300) and bare lymphocyte syndrome is classified as a immunological disease by Goh *et al*. [2].
228	978	Breast cancer	Melanoma	0.8682	Both are in cancer class based on Goh *et a*l.'s disease classification [2].
**110**	**1249**	**Anemia**	**Porphyria**	**0.8679**	**Deficiency in heme biosynthesis could cause both porphyrias and for X-linked sideroblastic anemia [32].**
**681**	**224**	**Hirschsprung disease**	**Brachydactyly**	**0.8675**	**Both are involved in deficiency in development [3, 18].**
**287**	**224**	**Central hypoventilation syndrome**	**Brachydactyly**	**0.8673**	**Both are involved in deficiency in development [3] .**
**689**	**166**	**Holoprosencephaly**	**Atrial fibrillation or Atrial septal defect**	**0.8673**	**Not available.**
1040	1059	Muscular dystrophy	Myopathy	0.8663	Both are in muscular class based on Goh *et al.*'s disease classification [2].
**1054**	**103**	**Myocardial infarction**	**Amyloidosis**	**0.8659**	**Approximately 40% of patients with primary amyloidosis have had cardiac amyloidosis [33]. Patients with cardiac amyloidosis have the risk of fatal myocardial infarction [33].**
**1272**	**1170**	**Prostate cancer**	**Ovarian cancer or carinoma**	**0.8655**	**Both are in cancer class based on Goh *et al.*'s disease classification [2].**

346	1178	Colon cancer	Pancreatic cancer or carcinoma	0.8650	Both are in cancer class based on Goh *et al*.'s disease classification [2].
**1383**	**164**	**Severe combined immunodeficiency**	**Atopy**	**0.8646**	**Both are in immunological class based on Goh *et al*.'s disease classification [2].**
**1043**	**9038**	**Mycobacterial infection or mycobacterium tuberculosis**	**Multiple sclerosis**	**0.8645**	**Multiple sclerosis is an autoimmune disease that affects the central nervous system [17]. Mycobacterial infection or mycobacterium tuberculosis is in immunological class based on Goh *et al*.'s disease classification [2].**
228	668	Breast cancer	Hepatocellular carcinoma	0.8640	Both are in cancer class based on Goh *et al*.'s disease classification [2].
**877**	**1615**	**Leigh syndrome**	**Zellweger syndrome**	**0.8640**	**Zellweger syndrome could have coupled Mitochondrial myopathy [30, 31]; Leigh syndrome is involved in deficiency in mitochondria (OMIM record: 256000).**
**873**	**1110**	**Leber congenital amaurosis**	**Night blindness**	**0.8631**	**Both are in ophthamological class based on Goh *et al*.'s disease classification [2].**
**1126**	**1020**	**Obesity**	**Maturity onset diabetes of the young**	**0.8622**	**Increases in body fat alter the body's response to insulin and lead to insulin resistance [34]. Maturity onset diabetes of the young involves in the defect in insulin production or response [10].**
346	978	Colon cancer	Melanoma	0.8612	Both are in cancer class based on Goh *et al*.'s disease classification [2].
**1272**	**668**	**Prostate cancer**	**Hepatocellular carcinoma**	**0.8607**	**Both are in cancer class based on Goh *et al*.'s disease classification [2].**
937	240	Macular degeneration	Complement system deficiency	0.8607	Complement C3 variant and complement factor H polymorphisms have been associated with age-related macular degeneration [35, 36].

1054	1497	Myocardial infarction	Thrombophilia	0.8602	Thrombophilia may contribute to the development of myocardial infarction in some young patients [37].
668	1178	Hepatocellular carcinoma	Pancreatic cancer or carcinoma	0.8596	Both are in cancer class based on Goh *et al*.'s disease classification [2].
**365**	**1054**	**Coronary artery or coronary heart disease**	**Myocardial infarction**	**0.8594**	**Both are in cardiovascular class based on Goh *et al*.'s disease classification [2].**
**98**	**724**	**Alzheimer disease**	**Hypercholesterolemia**	**0.8594**	**High cholesterol may play important roles in the development of Alzheimer's disease [19, 38, 39].**
1272	523	Prostate cancer	Fanconi anemia	0.8592	Fanconi anemia has strong predisposition to cancer [2].
1272	1503	Prostate cancer	Thyroid adenoma or carcinoma	0.8589	Both are in cancer class based on Goh *et al*.'s disease classification [2].
268	166	Cardiomyopathy	Atrial fibrillation or Atrial septal defect	0.8589	Both are in cardiovascular class based on Goh *et al*.'s disease classification [2].
98	412	Alzheimer disease	Dementia	0.8559	Both are in neurological class based on Goh *et al*.'s disease classification [2].
1316	1110	Retinitis pigmentosa	Night blindness	0.8545	Both are in ophthamological class based on Goh *et al*.'s disease classification [2].
**296**	**589**	**Ceroid-lipofuscinosis**	**Glaucoma**	**0.8535**	**Both are involved in abnormalities in lysosomal function [20, 40].**
346	572	Colon cancer	Gastric cancer	0.8533	Both are in cancer class based on Goh *et al*.'s disease classification [2].
**228**	**1608**	**Breast cancer**	**Xeroderma pigmentosum**	**0.8525**	**Xeroderma pigmentosum leads to skin cancer [7].**
**877**	**1218**	**Leigh syndrome**	**Pheochromocytoma**	**0.8518**	**Leigh syndrome is a mitochondrial disease (OMIM record: 256000). Mitochondrial deficiency might also play a role in Pheochromocytoma [41, 42].**

**1471**	**1383**	**Systemic lupus erythematosus**	**Severe combined immunodeficiency**	**0.8515**	**Both are in immunological class based on Goh *et al*.'s disease classification [2].**
523	1170	Fanconi anemia	Ovarian cancer or carinoma	0.8510	Fanconi anemia has strong predisposition to cancer [2].
**1042**	**187**	**Myasthenic syndrome**	**Bartter syndrome**	**0.8506**	**Both are involved in ion-transport deficiency [6, 43, 44].**
346	228	Colon cancer	Breast cancer	0.8505	Both are in cancer class based on Goh *et al*.'s disease classification [2].

Note: in the column of "Supporting evidence for association", evidence based on the descriptions of OMIM records is listed with corresponding OMIM record IDs.

References:

1. Steinberg SJ, Dodt G, Raymond GV, Braverman NE, Moser AB, Moser HW: **Peroxisome biogenesis disorders**. *Biochim Biophys Acta* 2006, **1763**(12):1733-1748.
2. Goh KI, Cusick ME, Valle D, Childs B, Vidal M, Barabasi AL: **The human disease network**. *Proc Natl Acad Sci U S A* 2007, **104**(21):8685-8690.
3. Oti M, Brunner HG: **The modular nature of genetic diseases**. *Clin Genet* 2007, **71**(1):1-11.
4. Mayan H, Vered I, Mouallem M, Tzadok-Witkon M, Pauzner R, Farfel Z: **Pseudohypoaldosteronism type II: marked sensitivity to thiazides, hypercalciuria, normomagnesemia, and low bone mineral density**. *J Clin Endocrinol Metab* 2002, **87**(7):3248-3254.
5. Pradervand S, Barker PM, Wang Q, Ernst SA, Beermann F, Grubb BR, Burnier M, Schmidt A, Bindels RJ, Gatzy JT *et al*: **Salt restriction induces pseudohypoaldosteronism type 1 in mice expressing low levels of the beta-subunit of the amiloride-sensitive epithelial sodium channel**. *Proc Natl Acad Sci U S A* 1999, **96**(4):1732-1737.

6. Koren W, Peleg E, Rosenthal T, Postnov YV: **Membrane ion transport in Bartter's syndrome: evidence for a new syndrome subtype**. *Hypertension* 1997, **30**(6):1338-1341.
7. Chen LlQWDJ: **DNA REPAIR, GENETIC INSTABILITY, AND CANCER**: World Scientific Publishing; 2007.
8. Atlas DH, Gaberman P, Stern K: **Primary amyloidosis associated with hyperlipemia and hypercholesterolemia; report of case**. *Am J Clin Pathol* 1950, **20**(4):371-376.
9. Rother KI: **Diabetes treatment--bridging the divide**. *N Engl J Med* 2007, **356**(15):1499-1501.
10. Fajans SS, Bell GI, Polonsky KS: **Molecular mechanisms and clinical pathophysiology of maturity-onset diabetes of the young**. *N Engl J Med* 2001, **345**(13):971-980.
11. Pierangeli SS, Girardi G, Vega-Ostertag M, Liu X, Espinola RG, Salmon J: **Requirement of activation of complement C3 and C5 for antiphospholipid antibody-mediated thrombophilia**. *Arthritis Rheum* 2005, **52**(7):2120-2124.
12. Pierangeli SS, Vega-Ostertag M, Liu X, Girardi G: **Complement activation: a novel pathogenic mechanism in the antiphospholipid syndrome**. *Ann N Y Acad Sci* 2005, **1051**:413-420.
13. Khan S, Dickerman JD: **Hereditary thrombophilia**. *Thromb J* 2006, **4**:15.
14. McNally EM: **New approaches in the therapy of cardiomyopathy in muscular dystrophy**. *Annu Rev Med* 2007, **58**:75-88.
15. Markiewski MM, Nilsson B, Ekdahl KN, Mollnes TE, Lambris JD: **Complement and coagulation: strangers or partners in crime?** *Trends Immunol* 2007, **28**(4):184-192.
16. Esmon CT: **Interactions between the innate immune and blood coagulation systems**. *Trends Immunol* 2004, **25**(10):536-542.
17. Goetz CG: **Textbook of Clinical Neurology**, 2 edn. Philadelphia: Saunders; 2003.
18. Temtamy SA, Aglan MS: **Brachydactyly**. *Orphanet J Rare Dis* 2008, **3**:15.
19. Hooijmans CR, Kiliaan AJ: **Fatty acids, lipid metabolism and Alzheimer pathology**. *Eur J Pharmacol* 2008, **585**(1):176-196.
20. Sedel F, Turpin JC, Baumann N: **[Neurological presentations of lysosomal diseases in adult patients]**. *Rev Neurol (Paris)* 2007, **163**(10):919-929.
21. Zangari M, Elice F, Fink L, Tricot G: **Hemostatic dysfunction in paraproteinemias and amyloidosis**. *Semin Thromb Hemost* 2007, **33**(4):339-349.
22. Vohra J: **The Long QT Syndrome**. *Heart Lung Circ* 2007, **16 Suppl 3**:S5-12.
23. Caligiuri G, Levy B, Pernow J, Thoren P, Hansson GK: **Myocardial infarction mediated by endothelin receptor signaling in hypercholesterolemic mice**. *Proc Natl Acad Sci U S A* 1999, **96**(12):6920-6924.
24. Lai D, Schroer B: **Haddad syndrome: a case of an infant with central congenital hypoventilation syndrome and Hirschsprung disease**. *J Child Neurol* 2008, **23**(3):341-343.

25. Mahmoud M, Bryan Y, Gunter J, Kreeger RN, Sadhasivam S: **Anesthetic implications of undiagnosed late onset central hypoventilation syndrome in a child: from elective tonsillectomy to tracheostomy**. *Paediatr Anaesth* 2007, **17**(10):1001-1005.
26. Anderson KE, Bloomer JR, Bonkovsky HL, Kushner JP, Pierach CA, Pimstone NR, Desnick RJ: **Recommendations for the diagnosis and treatment of the acute porphyrias**. *Ann Intern Med* 2005, **142**(6):439-450.
27. Yasuda M, Kawarabayashi T, Akioka K, Teragaki M, Oku H, Kanayama Y, Takeuchi K, Takeda T, Kawase Y, Ikuno Y: **The complement system in the acute phase of myocardial infarction**. *Jpn Circ J* 1989, **53**(9):1017-1022.
28. Dobyns WB, Pagon RA, Armstrong D, Curry CJ, Greenberg F, Grix A, Holmes LB, Laxova R, Michels VV, Robinow M *et al*: **Diagnostic criteria for Walker-Warburg syndrome**. *Am J Med Genet* 1989, **32**(2):195-210.
29. Hafer-Macko CE, Dyck PJ, Koski CL: **Complement activation in acquired and hereditary amyloid neuropathy**. *J Peripher Nerv Syst* 2000, **5**(3):131-139.
30. Sarnat HB, Machin G, Darwish HZ, Rubin SZ: **Mitochondrial myopathy of cerebro-hepato-renal (Zellweger) syndrome**. *Can J Neurol Sci* 1983, **10**(3):170-177.
31. Muller-Hocker J, Walther JU, Bise K, Pongratz D, Hubner G: **Mitochondrial myopathy with loosely coupled oxidative phosphorylation in a case of Zellweger syndrome. A cytochemical-ultrastructural study**. *Virchows Arch B Cell Pathol Incl Mol Pathol* 1984, **45**(2):125-138.
32. Ajioka RS, Phillips JD, Kushner JP: **Biosynthesis of heme in mammals**. *Biochim Biophys Acta* 2006, **1763**(7):723-736.
33. Kotani N, Hashimoto H, Muraoka M, Kabara S, Okawa H, Matsuki A: **Fatal perioperative myocardial infarction in four patients with cardiac amyloidosis**. *Anesthesiology* 2000, **92**(3):873-875.
34. Keller U: **From obesity to diabetes**. *Int J Vitam Nutr Res* 2006, **76**(4):172-177.
35. Thakkinstian A, Han P, McEvoy M, Smith W, Hoh J, Magnusson K, Zhang K, Attia J: **Systematic review and meta-analysis of the association between complement factor H Y402H polymorphisms and age-related macular degeneration**. *Hum Mol Genet* 2006, **15**(18):2784-2790.
36. Yates JR, Sepp T, Matharu BK, Khan JC, Thurlby DA, Shahid H, Clayton DG, Hayward C, Morgan J, Wright AF *et al*: **Complement C3 variant and the risk of age-related macular degeneration**. *N Engl J Med* 2007, **357**(6):553-561.
37. Segev A, Ellis MH, Segev F, Friedman Z, Reshef T, Sparkes JD, Tetro J, Pauzner H, David D: **High prevalence of thrombophilia among young patients with myocardial infarction and few conventional risk factors**. *Int J Cardiol* 2005, **98**(3):421-424.
38. Anstey KJ, Lipnicki DM, Low LF: **Cholesterol as a risk factor for dementia and cognitive decline: a systematic review of prospective studies with meta-analysis**. *Am J Geriatr Psychiatry* 2008, **16**(5):343-354.

39. Xiong H, Callaghan D, Jones A, Walker DG, Lue LF, Beach TG, Sue LI, Woulfe J, Xu H, Stanimirovic DB *et al*: **Cholesterol retention in Alzheimer's brain is responsible for high beta- and gamma-secretase activities and Abeta production**. *Neurobiol Dis* 2008, **29**(3):422-437.
40. Hayasaka S: **Lysosomal enzymes in ocular tissues and diseases**. *Surv Ophthalmol* 1983, **27**(4):245-258.
41. Favier J, Briere JJ, Strompf L, Amar L, Filali M, Jeunemaitre X, Rustin P, Gimenez-Roqueplo AP: **Hereditary paraganglioma/pheochromocytoma and inherited succinate dehydrogenase deficiency**. *Horm Res* 2005, **63**(4):171-179.
42. Dahia PL, Ross KN, Wright ME, Hayashida CY, Santagata S, Barontini M, Kung AL, Sanso G, Powers JF, Tischler AS *et al*: **A HIF1alpha regulatory loop links hypoxia and mitochondrial signals in pheochromocytomas**. *PLoS Genet* 2005, **1**(1):72-80.
43. Croxen R, Hatton C, Shelley C, Brydson M, Chauplannaz G, Oosterhuis H, Vincent A, Newsom-Davis J, Colquhoun D, Beeson D: **Recessive inheritance and variable penetrance of slow-channel congenital myasthenic syndromes**. *Neurology* 2002, **59**(2):162-168.
44. Tsujino A, Maertens C, Ohno K, Shen XM, Fukuda T, Harper CM, Cannon SC, Engel AG: **Myasthenic syndrome caused by mutation of the SCN4A sodium channel**. *Proc Natl Acad Sci U S A* 2003, **100**(12):7377-7382.
