# Supplementary material for: Genome-wide prioritization of disease genes and identification of disease-disease associations from an integrated human functional linkage network
Source: Genome Biol. 2009 Sep 3;10(9):R91. doi: 10.1186/gb-2009-10-9-r91 (PMC2768980; doi:10.1186/gb-2009-10-9-r91)
Supplement: Additional data file 10 — Disease clusters, their disease members, and evidence supporting the associations in Figure S9 of Additional data file 5 and Figure 8a. [file gb-2009-10-9-r91-S10.pdf]

List of the disease clusters, their disease members, and evidence supporting the association for Figure 8A of the main manuscript and Figure S7 of Additional File 5.

Cluster name	Disease names of members	Disease ID of members	Supporting evidence
**Cancer**	Thyroid adenoma or carcinoma	1503	in cancer class based on Goh *et al*.'s disease classification [1].
	Prostate cancer	1272	in cancer class based on Goh *et al*.'s disease classification [1].
	Hepatocellular carcinoma	668	in cancer class based on Goh *et al*.'s disease classification [1].
	Gastric cancer	572	in cancer class based on Goh *et al*.'s disease classification [1].
	Pancreatic cancer or carcinoma	1178	in cancer class based on Goh *et al*.'s disease classification [1].
	Ovarian cancer or carinoma	1170	in cancer class based on Goh *et al*.'s disease classification [1].
	Colon cancer	346	in cancer class based on Goh *et al*.'s disease classification [1].
	Melanoma	978	in cancer class based on Goh et al.'s disease classification [1].
	Breast cancer	228	in cancer class based on Goh *et al*.'s disease classification [1].
	Fanconi anemia	523	Fanconi anemia has strong predisposition to cancer [1].
	Xeroderma pigmentosum	1608	Xeroderma pigmentosum leads to skin cancer [2].
**Deficiency in insulin production or response**	Diabetes mellitus	427	involved in diminished production of insulin or diminished response to insulin [3, 4].
	Maturity onset diabetes of the young	1020	involved in diminished production of insulin or diminished response to insulin [3, 4].
	Obesity	1126	Increases in body fat alter the body's response to insulin leading to insulin resistance [5].

Cluster name	Disease names of members	Disease ID of members	Supporting evidence
**Immunological diseases**	Atopy	164	in immunological class based on Goh *et al*.'s disease classification [1]
	Severe combined immunodeficiency	1383	in immunological class based on Goh *et al*.'s disease classification [1]
	Systemic lupus erythematosus	1471	in immunological class based on Goh *et al*.'s disease classification [1]
	Multiple sclerosis	9038	Multiple sclerosis is an autoimmune disease that affects the central nervous system [6].
	Malaria	940	in immunological class based on Goh *et al*.'s disease classification [1]
	Mycobacterial infection or mycobacterium tuberculosis	1043	in immunological class based on Goh *et al*.'s disease classification [1]
	Immunodeficiency due to defect in CD3 or with hyper-IgM	802	in immunological class based on Goh *et al*.'s disease classification [1]
	Rheumatoid arthritis	1324	Rheumatoid arthritis has autoimmune features (OMIM record: 180300).
	Bare lymphocyte syndrome	184	in immunological class based on Goh *et al*.'s disease classification [1]
**Deficiency in lysosomal function**	Mucopolysaccharidosis	1033	involved in abnormalities of lysosomal function [7].
	Ceroid-lipofuscinosis	296	involved in abnormalities of lysosomal function [7].
	Glaucoma	589	involved in abnormalities in lysosomal function [7, 8].
**Peroxisome biogenesis disorders**	Zellweger syndrome	1615	peroxisome biogenesis disorder [9]
	Refsum disease	1307	peroxisome biogenesis disorder [9]
	Adrenoleukodystrophy	56	peroxisome biogenesis disorder [9]

Cluster name	Disease names of members	Disease ID of members	Supporting evidence
**Deficiency in development**	Waardenburg syndrome	1574	Involved in deficiency in developmental process [10].
	Central hypoventilation syndrome	287	Involved in deficiency in developmental process [10].
	Holoprosencephaly	689	Involved in deficiency in development (OMIM record: 236100).
	Hirschsprung disease	681	Involved in deficiency in developmental process [10].
	Brachydactyly	224	Involved in deficiency in development [11]
**Deficiency in mitochondria**	Leigh syndrome	877	Deficiency in mitochondria (OMIM record: 256000)
	Combined oxidative phosphorylation deficiency	6350	Deficiency in mitochondria (OMIM record: 609060, 610498, 610678, 611719, 610505).
	Mitochondrial DNA depletion syndrome	5016	Deficiency in mitochondria (OMIM record: 251880, 609560).
	Mitochondrial complex I/II/III deficiency	1016	Deficiency in mitochondria (OMIM record: 252010, 124000, 252011)
**Deficiency in ion transport**	Long QT syndrome	912	Involved in ion-transport deficiency [12, 13].
	Bartter syndrome	187	Involved in ion-transport deficiency [12, 13].
	Pseudohypoaldosteronism	1281	Involved in ion-transport deficiency [13-15].
	Myasthenic syndrome	1042	Involved in ion-transport deficiency [13, 16, 17].

Cluster name	Disease names of members	Disease ID of members	Supporting evidence
**Cardiovascular or muscular diseases**	Cardiomyopathy	268	In cardiovascular class based on Goh *et al.*'s disease classification [1].
	Atrial fibrillation or Atrial septal defect	166	In cardiovascular class based on Goh *et al.*'s disease classification [1].
	Long QT syndrome	912	In cardiovascular class based on *Goh et al.*'s disease classification [1].
	Muscular dystrophy	1040	Muscular dystrophy in muscular class based on Goh *et al.*'s disease classification [1]. Some diseases in cardiovascular class such as "long QT syndrome " [18-20] and cardiomyopathy involve in muscle contraction deficiency (OMIM record: 115200, 500000, 192600, 601154, 600884, 600858).
	Myopathy	1059	Myopathy is in muscular class based on Goh *et al.*'s disease classification [1]. Some diseases in cardiovascular class such as "long QT syndrome " [18-20] and cardiomyopathy involve in muscle contraction deficiency (OMIM record: 115200, 500000, 192600, 601154, 600884, 600858).
**Ophthamological diseases**	Night blindness	1110	In ophthamological class based on Goh *et al.*'s disease classification [1].
	Leber congenital amaurosis	873	In ophthamological class based on Goh *et al.*'s disease classification [1].
	Retinitis pigmentosa	1316	In ophthamological class based on Goh *et al.*'s disease classification [1].
	Cone or cone-rod dystrophy	352	In ophthamological class based on Goh *et al.*'s disease classification [1].

References:

1. Goh KI, Cusick ME, Valle D, Childs B, Vidal M, Barabasi AL: **The human disease network**. *Proc Natl Acad Sci U S A* 2007, **104**(21):8685-8690.
2. Chen LlQWDJ: **DNA REPAIR, GENETIC INSTABILITY, AND CANCER**: World Scientific Publishing; 2007.
3. Rother KI: **Diabetes treatment--bridging the divide**. *N Engl J Med* 2007, **356**(15):1499-1501.
4. Fajans SS, Bell GI, Polonsky KS: **Molecular mechanisms and clinical pathophysiology of maturity-onset diabetes of the young**. *N Engl J Med* 2001, **345**(13):971-980.
5. Keller U: **From obesity to diabetes**. *Int J Vitam Nutr Res* 2006, **76**(4):172-177.
6. Goetz CG: **Textbook of Clinical Neurology**, 2 edn. Philadelphia: Saunders; 2003.
7. Sedel F, Turpin JC, Baumann N: **[Neurological presentations of lysosomal diseases in adult patients]**. *Rev Neurol (Paris)* 2007, **163**(10):919-929.
8. Hayasaka S: **Lysosomal enzymes in ocular tissues and diseases**. *Surv Ophthalmol* 1983, **27**(4):245-258.
9. Steinberg SJ, Dodt G, Raymond GV, Braverman NE, Moser AB, Moser HW: **Peroxisome biogenesis disorders**. *Biochim Biophys Acta* 2006, **1763**(12):1733-1748.
10. Oti M, Brunner HG: **The modular nature of genetic diseases**. *Clin Genet* 2007, **71**(1):1-11.
11. Temtamy SA, Aglan MS: **Brachydactyly**. *Orphanet J Rare Dis* 2008, **3**:15.
12. Vohra J: **The Long QT Syndrome**. *Heart Lung Circ* 2007, **16 Suppl 3**:S5-12.
13. Koren W, Peleg E, Rosenthal T, Postnov YV: **Membrane ion transport in Bartter's syndrome: evidence for a new syndrome subtype**. *Hypertension* 1997, **30**(6):1338-1341.
14. Mayan H, Vered I, Mouallem M, Tzadok-Witkon M, Pauzner R, Farfel Z: **Pseudohypoaldosteronism type II: marked sensitivity to thiazides, hypercalciuria, normomagnesemia, and low bone mineral density**. *J Clin Endocrinol Metab* 2002, **87**(7):3248-3254.
15. Pradervand S, Barker PM, Wang Q, Ernst SA, Beermann F, Grubb BR, Burnier M, Schmidt A, Bindels RJ, Gatzy JT *et al.*: **Salt restriction induces pseudohypoaldosteronism type 1 in mice expressing low levels of the beta-subunit of the amiloride-sensitive epithelial sodium channel**. *Proc Natl Acad Sci U S A* 1999, **96**(4):1732-1737.

16. Croxen R, Hatton C, Shelley C, Brydson M, Chauplannaz G, Oosterhuis H, Vincent A, Newsom-Davis J, Colquhoun D, Beeson D: **Recessive inheritance and variable penetrance of slow-channel congenital myasthenic syndromes**. *Neurology* 2002, **59**(2):162-168.
17. Tsujino A, Maertens C, Ohno K, Shen XM, Fukuda T, Harper CM, Cannon SC, Engel AG: **Myasthenic syndrome caused by mutation of the SCN4A sodium channel**. *Proc Natl Acad Sci U S A* 2003, **100**(12):7377-7382.
18. Goldenberg I, Moss AJ: **Long QT syndrome**. *J Am Coll Cardiol* 2008, **51**(24):2291-2300.
19. Sauer AJ, Moss AJ, McNitt S, Peterson DR, Zareba W, Robinson JL, Qi M, Goldenberg I, Hobbs JB, Ackerman MJ *et al.*: **Long QT syndrome in adults**. *J Am Coll Cardiol* 2007, **49**(3):329-337.
20. Crotti L, Celano G, Dagradi F, Schwartz PJ: **Congenital long QT syndrome**. *Orphanet J Rare Dis* 2008, **3**:18.
